# Global variability of the human IgG glycome

**DOI:** 10.18632/aging.103884

**Published:** 2020-08-12

**Authors:** Jerko Štambuk, Natali Nakić, Frano Vučković, Maja Pučić-Baković, Genadij Razdorov, Irena Trbojević-Akmačić, Mislav Novokmet, Toma Keser, Marija Vilaj, Tamara Štambuk, Ivan Gudelj, Mirna Šimurina, Manshu Song, Hao Wang, Marijana Peričić Salihović, Harry Campbell, Igor Rudan, Ivana Kolčić, Leigh Anne Eller, Paul McKeigue, Merlin L. Robb, Jonas Halfvarson, Metin Kurtoglu, Vito Annese, Tatjana Škarić-Jurić, Mariam Molokhia, Ozren Polašek, Caroline Hayward, Hannah Kibuuka, Kujtim Thaqi, Dragan Primorac, Christian Gieger, Sorachai Nitayaphan, Tim Spector, Youxin Wang, Therese Tillin, Nish Chaturvedi, James F. Wilson, Moses Schanfield, Maxim Filipenko, Wei Wang, Gordan Lauc

**Affiliations:** 1 Genos Glycoscience Research Laboratory, Zagreb, Croatia; 2 Department of Neuroscience, Scuola Internazionale Superiore di Studi Avanzati (SISSA), Trieste, Italy; 3 Faculty of Pharmacy and Biochemistry, University of Zagreb, Zagreb, Croatia; 4 Beijing Key Laboratory of Clinical Epidemiology, School of Public Health, Capital Medical University, Beijing, China; 5 School of Medical and Health Sciences, Edith Cowan University, Perth, Australia; 6 Institute for Anthropological Research, Zagreb, Croatia; 7 Centre for Global Health Research, Usher Institute of Population Health Sciences and Informatics, The University of Edinburgh, Edinburgh, United Kingdom; 8 School of Medicine, University of Split, Split, Croatia; 9 Walter Reed Army Institute of Research, Silver Spring, MD 20910, USA; 10 Henry M. Jackson Foundation for the Advancement of Military Medicine, Bethesda, MD 20817, USA; 11 Department of Gastroenterology, Faculty of Medicine and Health, Örebro University, Örebro, Sweden; 12 Department of Oncology, Koç University School of Medicine, Istanbul, Turkey; 13 Careggi University Hospital, Florence, Italy; 14 School of Population Health and Environmental Sciences, King's College London, London, United Kingdom; 15 MRC Human Genetics Unit, MRC Institute for Genetics and Molecular Medicine, University of Edinburgh, Edinburgh, United Kingdom; 16 Makerere University Walter Reed Project, Kampala, Uganda; 17 Institute of Clinical Biochemistry, Priština, Kosovo; 18 St. Catherine Hospital, Zagreb, Croatia; 19 Helmholtz Zentrum München - German Research Center for Environmental Health, Neuherberg, Germany; 20 Armed Forces Research Institute of Medical Sciences, Bangkok, Thailand; 21 Department of Twin Research and Genetic Epidemiology, King's College London, London, United Kingdom; 22 Institute of Cardiovascular Science, Faculty of Population Health Sciences, London, United Kingdom; 23 Department of Forensic Sciences, George Washington University, Washington, DC 20007, USA; 24 Institute of Chemical Biology and Fundamental Medicine, Novosibirsk, Russia

**Keywords:** glycans, aging, immunoglobulin G, Fc glycosylation, mass spectrometry

## Abstract

Immunoglobulin G (IgG) is the most abundant serum antibody which structural characteristics and effector functions are modulated through the attachment of various sugar moieties called glycans. Composition of the IgG N-glycome changes with age of an individual and in different diseases. Variability of IgG glycosylation within a population is well studied and is known to be affected by both genetic and environmental factors. However, global inter-population differences in IgG glycosylation have never been properly addressed. Here we present population-specific N-glycosylation patterns of IgG, analyzed in 5 different populations totaling 10,482 IgG glycomes, and of IgG’s fragment crystallizable region (Fc), analyzed in 2,579 samples from 27 populations sampled across the world. Country of residence associated with many N-glycan features and the strongest association was with monogalactosylation where it explained 38% of variability. IgG monogalactosylation strongly correlated with the development level of a country, defined by United Nations health and socioeconomic development indicators, and with the expected lifespan. Subjects from developing countries had low levels of IgG galactosylation, characteristic for inflammation and ageing. Our results suggest that citizens of developing countries may be exposed to environmental factors that can cause low-grade chronic inflammation and the apparent increase in biological age.

## INTRODUCTION

Immunoglobulin G is the most common antibody class circulating in human blood [1]. It mediates interactions between antigens and the immune system [2]. There are four IgG subclasses present in plasma, which differ in the constant region of the molecule: IgG1, IgG2, IgG3 and IgG4 [3]. Each subclass has distinctive features and functions, such as pronounced affinity for certain types of antigens, formation of immune complexes, complement activation, interactions with effector cells, half-life and placental transport [2]. Every IgG molecule contains covalently attached N-linked glycans which are essential for some of its functions [4].

Glycosylation is a co- and post-translational modification which is orchestrated by a complex biosynthetic pathway [5]. IgG contains a conserved N-glycosylation site on Asn^297^ residue within its fragment crystallizable (Fc) region, on each of the two identical heavy chains [6]. Glycans attached to IgG are mainly of a complex biantennary type, with the core structure consisting of four *N*-acetylglucosamines (GlcNAc) and three mannoses. Different glycan moieties such as bisecting GlcNAc, galactose, sialic acid and fucose can be attached to this core [4]. IgG shows a high degree of diversity in glycosylation, with each of the four IgG subclasses displaying a distinctive glycome composition [7]. Also, each of the heavy chains of the same molecule can carry different glycans, creating a large repertoire of possible IgG glycoforms [8]. Finally, in 15-20% of cases, an additional N-glycosylation site appears within variable region of antigen-binding fragment (Fab), as a result of somatic hypermutation events during affinity maturation [9].

Many IgG functions are achieved through interactions with receptors on immune cells and complement proteins. Fc glycans affect immunoglobulin conformation, which, in turn, defines binding affinity for Fc gamma receptors (FcγRs) on effector cells and complement, leading to alterations in effector functions [1, 10, 11]. Furthermore, IgG galactosylation level has an extensive effect on its inflammatory potential [12]. Namely, agalactosylated IgG has increased inflammatory potential through activation of alternative complement pathway, while on the other hand, high level of galactose is necessary for activation of anti-inflammatory cascade through interactions with FcγRIIB and inhibition of the inflammatory activity of C5a complement component [13–16]. However, there are also reports suggesting pro-inflammatory action of highly galactosylated IgG. Terminal IgG galactosylation is required for increased binding to activating Fc gamma receptors and therefore activation of antibody-dependent cellular cytotoxicity (ADCC) [17]. Also, terminal galactoses are necessary for C1q complement component binding and activation of complement-dependent cytotoxicity (CDC) [18]. Attachment of other sugar moieties affects antibody properties as well. Namely, presence of fucose attached to the first N-acetylglucosamine, i.e. core fucose, decreases ADCC activity, while the presence of bisecting GlcNAc increases binding affinity for activating Fcγ receptors [19]. Terminal sialic acids appear to contribute to enhanced anti-inflammatory activity of intravenous immunoglobulin (IVIg) [20]. Although a subject to debate, proposed mechanisms include reduced affinity of sialylated IgG for activating FcγRs, and increased recognition by lectin receptors and complement component C1q [21, 22].

There is a prominent inter-individual variability of the total IgG N-glycome, which is under strong influence of numerous genes and environmental factors [23]. Average IgG glycome heritability is estimated to approximately 50%, while the remaining variability can be mostly attributed to environmental factors [23–25]. Prominent changes in the IgG N-glycome composition were found in several diseases. In different autoimmune and alloimmune disorders, cancers and infectious diseases, changes in IgG glycosylation reflect the increased inflammation which usually accompanies these conditions [12]. The impact of IgG glycosylation on its ability to modulate inflammation has been extensively studied as a potential biomarker for disease prognosis and treatment response, as well as for monoclonal antibody development [26, 27].

The composition of IgG N-glycome is also strongly influenced by sex hormones, age, and lifestyle such as smoking and body mass index [12, 28, 29]. Functional relevance of the impact of sex hormones on IgG glycosylation is notable through pregnancy-related remission in rheumatoid arthritis patients. Namely, the third trimester of pregnancy is characterized by anti-inflammatory IgG glycan profile and disease remission, due to high estradiol levels, while in post-partum period hormone levels decrease and IgG glycan profile changes back to pro-inflammatory, with a high risk of disease resurgence [30]. Since estrogen levels change during lifetime in women, sex-specific changes in glycosylation patterns can be observed, especially in levels of IgG galactosylation [31].

Ageing is a process of damage aggregation in an organism, characterized by increase of inflammation and decline in health, leading to disease and death [32]. It is influenced by both genetic factors and environment. Complex changes in IgG N-glycome have been reported during ageing, with the most extensive changes being related to the level of galactosylation. Namely, digalactosylated structures decrease, while agalactosylation increases with age [28]. Level of bisecting GlcNAc also increases with age, while changes in level of sialylation and core fucosylation displayed inconsistent trends in different studies [28]. IgG glycans have been shown to be more reliable estimators of age compared to other biomarkers, explaining up to 64% of the variation in chronological age [12]. Mechanisms underlying age-specific changes in galactosylation levels remain mostly unknown. Since ageing is an inflammation-related process, it has been proposed that chronic low-grade inflammation in older individuals decreases IgG galactosylation. On the other hand, undergalactosylated IgG exerts pro-inflammatory potential and by this positive feedback loop contributes to biological ageing [33, 34].

Despite the fact that structural and functional aspects of IgG glycosylation are intensively studied and associated with predisposition and course of different diseases, little is known about the regulation of IgG glycosylation or mechanisms that lead to extensive changes in glycome composition after environmental challenge [12, 35, 36]. Therefore, the focus of this study was to analyze and compare IgG N-glycosylation patterns in various populations and communities across the world, marked by their different genetic background and socioeconomic factors.

## RESULTS

In this study, we analyzed total IgG glycans and subclass specific Fc glycopeptides from various countries and communities. In both cases we observed significant changes in IgG glycosylation associated with age, sex and country of residence, where age and country of residence were able to explain a significant portion of glycosylation variability. IgG glycans also strongly correlated with the development level of a country and with specific development indicators as well.

### Total IgG glycans change with chronological age and sex

In the initial analysis, samples originating from 10,482 individuals and 5 different general populations (Supplementary Table 1) were analyzed. Fluorescently labelled N-glycans released from IgG were chromatographically profiled and separated into 24 chromatographic peaks (Supplementary Figure 1, Supplementary Table 2). This approach enables analysis of total IgG N-glycans (i.e., from both Fab and Fc parts of the molecule). Additionally, derived glycan traits (agalactosylation, monogalactosylation, digalactosylation, core fucosylation, sialylation and presence of bisecting GlcNAc) were calculated, based on the initial 24 glycan measures (calculation of derived glycan traits can be found in Supplementary Table 3) [23]. In general, agalactosylation levels showed the highest dispersion of all tested glycan traits (Q1=20%, Q3=32%; Supplementary Table 4), which coincides with the previous studies.

**Figure 1:**
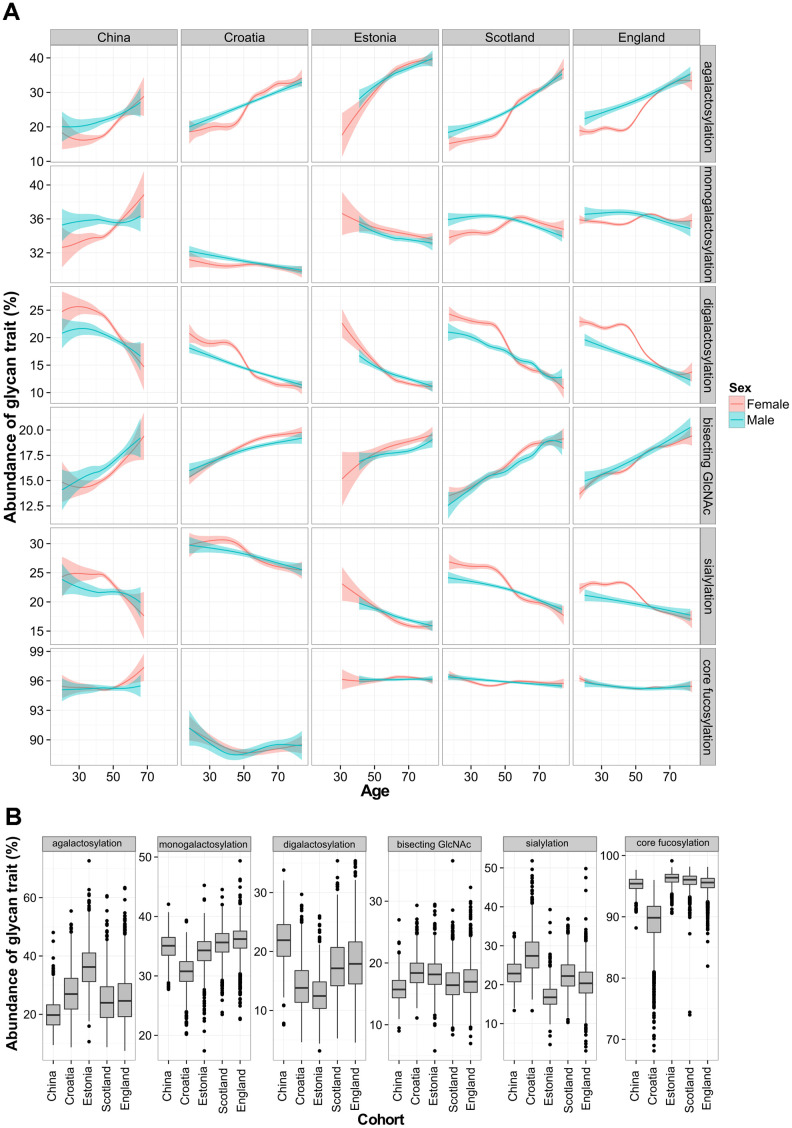
**Total IgG glycan levels in five different populations.** Relationship between age and derived glycan trait (**A**). Plots describe associations between each of the five glycan traits and chronological age of participant. Blue and red curves represent fitted linear regression models. The shaded region is the 95 % confidence interval on the fitted values. Differences in total IgG glycosylation between participants from five different populations (**B**). Each box represents interquartile range (25^th^ to 75^th^ percentiles). Lines inside the boxes represent the median values, while lines outside the boxes represent the 10^th^ and 90^th^ percentiles. Dots indicate outliers.

It is known that the chronological age of a subject affects IgG glycosylation [28]. Age-related changes were observed in the levels of various IgG glycan traits in all studied populations. Agalactosylated species and glycans containing bisecting GlcNAc increased with the chronological age of the participant. The opposite trend was observed in the levels of digalactosylated and sialylated glycans, which were decreasing with chronological age. On the other hand, core fucosylation and monogalactosylation levels did not change consistently with age. Furthermore, age-related changes in glycosylation displayed sex-specific patterns. Namely, female participants displayed characteristic increase in agalactosylated glycan species at the age of 50, which was not observed in the male population (Figure 1A).

### Age and country of residence explain most of variability in IgG glycosylation

Although total IgG N-glycans showed similar age-related changes within each of the studied cohorts, every population displayed particular glycan patterns. Again, the most pronounced differences between populations were observed in the levels of agalactosylated glycans, which increased with a median age of the analyzed population (Figure 1B). This glycan trait had the lowest median value in young Chinese cohort (20%), while the highest value was observed in Estonian cohort (36%), which was the oldest. Besides agalactosylation, pronounced differences between populations were also observed in the levels of digalactosylated and sialylated glycans (Supplementary Table 4). A linear mixed model was used to further elucidate changes in IgG glycan traits in different populations. Relations of age, country of residence and sex with the total IgG glycans were evaluated. Chronological age was able to moderately explain variability of total IgG glycan traits – for digalactosylation and agalactosylation it explained up to 31% of their variability. Contrary to age, participant’s country of residence was able to explain larger portion of variability of core fucose levels (*P*<6×10^-350^, n=5), with 57 % of the variability in this glycan trait explained. It was also able to account for the portion of monogalactosylation and sialylation variability. On the other hand, sex was able to explain less than 1% of the variability of any tested glycan trait (Supplementary Table 5).

### Fragment crystallizable glycan patterns of 27 different populations

**Figure 2:**
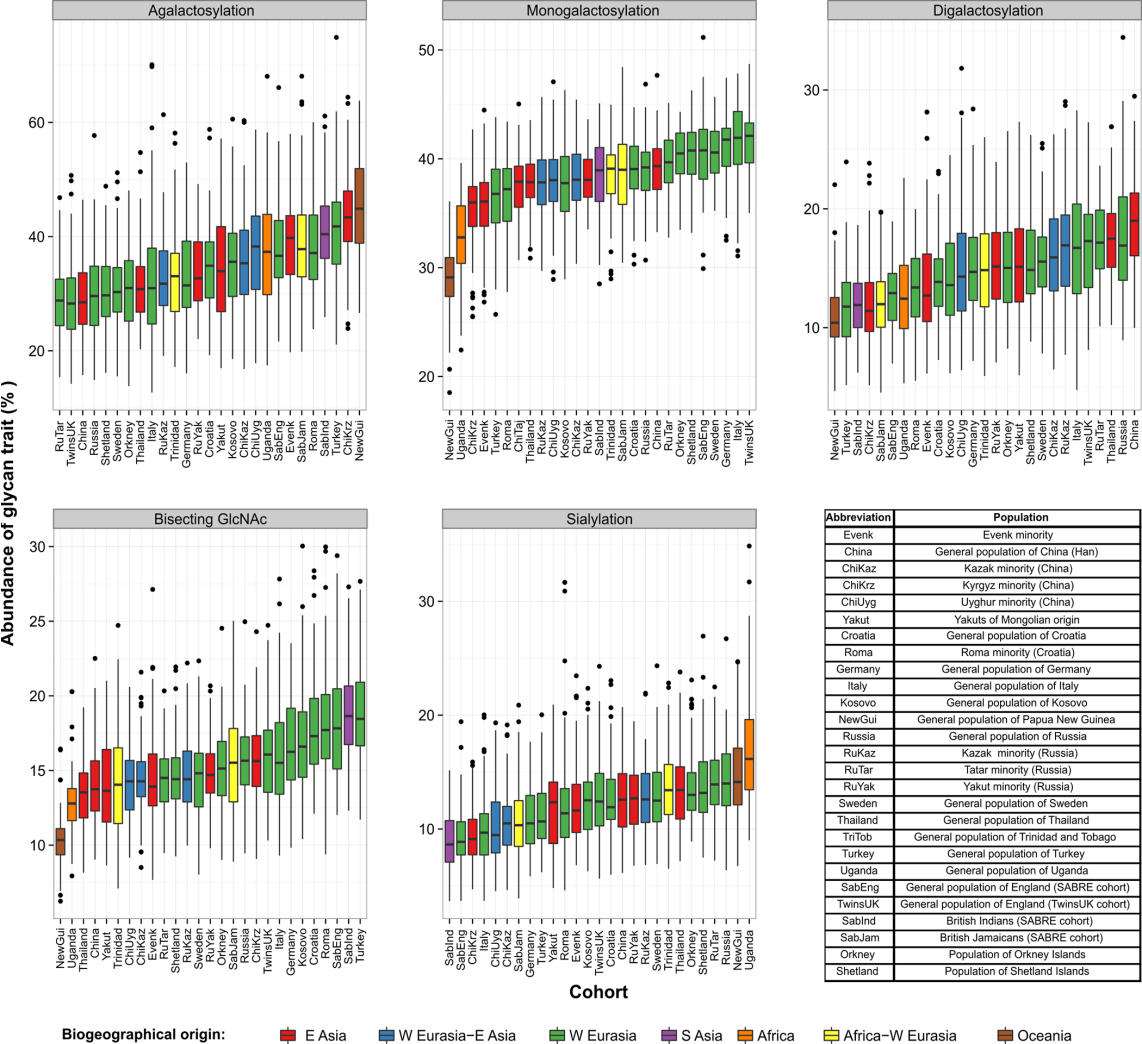
**Levels of derived IgG1 Fc glycan traits across 27 different populations collected worldwide.** Each box represents interquartile range (25^th^ to 75^th^ percentiles) with median values drawn as the middle line. Whiskers outside the boxes represent the 10^th^ and 90^th^ percentiles, while dots indicate outliers.

To validate observed diversity and unambiguously determine IgG N-glycosylation patterns in different populations (Supplementary Table 6), while eliminating potential batch effects, we compared glycan features derived from IgG subclass-specific Fc glycopeptides from 2,579 individuals (Supplementary Table 7, Supplementary Figure 2). This part of the study included 27 populations collected in 14 different countries. Subclass-specific glycopeptides were chromatographically separated and accurate masses were measured for each glycoform. Structures of IgG glycopeptides were confirmed using tandem mass spectrometry (MS/MS) analysis of a pooled sample (Supplementary Figures 3–5). Calculated IgG Fc N-glycan derived traits displayed considerable dispersion between analyzed populations (Figure 2). The Fc N-glycome composition is known to differ from the total IgG N-glycome, as a result of Fab N-glycome contribution to the total IgG glycome [37]. Again, the most prominent variation appeared to be in the level of galactosylation related traits, especially agalactosylation (Supplementary Tables 7–9). On the other hand, expected decrease in digalactosylation levels with the population’s age was not observed. On the contrary, some populations appeared to have lower than expected monogalactosylation and digalactosylation levels for the given chronological age. Population from Papua New Guinea, as the youngest one, surprisingly had the highest median level of agalactosylation (45 %), while the subjects from England exhibited the lowest levels of this glycan trait (28 %) on IgG1 subclass. The opposite effect was observed for monogalactosylation levels - the subjects from Papua New Guinea had the lowest median value of this glycan trait, while the highest levels were observed for the participants from England. In a similar manner, participants from countries such as Germany and Italy had higher monogalactosylation levels (comparable to subjects from England) than the ones from countries such as Uganda (similar to subjects from Papua New Guinea; Supplementary Table 7).

In the case of IgG2 and IgG4 subclasses, galactosylation related glycan traits displayed similar variation as for IgG1 subclass, although the observed glycosylation patterns appeared to be somewhat subclass-specific, especially in case of IgG4, which is the least abundant subclass in the human plasma (Supplementary Tables 8 and 9).

### Age and country of residence can explain IgG Fc glycosylation variability

To determine the relationship between IgG Fc glycan traits and sex, chronological age and country of residence, linear mixed model was used. The same as in the case of total IgG glycans, chronological age was able to explain a considerable portion of agalactosylation and digalactosylation variability. It was able to explain 28 % of IgG2 agalactosylation variability compared to 22 % for IgG1 subclass. Country of residence was able to explain the highest portion of IgG Fc monogalactosylation variability (Table 1). Namely, 38 % of IgG1 Fc monogalactosylation variability could be explained with the subject’s country of residence. Here as well, sex was able to explain up to 1% of the IgG Fc glycan variability. Glycan patterns similar to IgG1 subclass were observed for IgG2 and IgG4.

**Table 1:** Proportion of glycan feature variability in 14 countries explained by linear mixed model, with age and sex defined as fixed effects and country of residence as a random effect.

**IgG subclass**	**Glycan feature**	**Percentage of glycan trait variability explained by country of residence (%)**	**Percentage of glycan trait variability explained by age (%)**	**Percentage of glycan trait variability explained by sex (%)**	**Country of residence *P* value**
	Agalactosylation	21.4	18.3	0.9	1.03 × 10^-111^
	Monogalactosylation	38.0	2.6	0.1	7.69 × 10^-193^
IgG1	Digalactosylation	18.6	21.7	1.1	3.51 × 10^-98^
	Sialylation	10.8	13.2	0.6	1.27 × 10^-54^
	Bisecting GlcNAc	18.6	17.5	0.0	9.28 × 10^-110^
	Agalactosylation	12.8	23.9	0.9	7.18 × 10^-62^
	Monogalactosylation	20.8	7.6	0.1	3.34 × 10^-94^
IgG2	Digalactosylation	10.4	27.5	1.1	2.70 × 10^-54^
	Sialylation	6.5	18.2	0.5	1.22 × 10^-27^
	Bisecting GlcNAc	12.4	11.9	0.1	3.66 × 10^-64^
	Agalactosylation	20.5	13.7	0.4	1.32 × 10^-93^
	Monogalactosylation	20.8	2.6	0.1	1.30 × 10^-78^
IgG4	Digalactosylation	18.2	14.1	0.8	1.28 × 10^-86^
	Sialylation	15.4	9.7	0.5	6.44 × 10^-75^
	Bisecting GlcNAc	7.6	15.7	0.6	2.01 × 10^-37^

Displayed values represent percentage (%) of glycan trait variability explained by country, age and sex.

### IgG Fc galactosylation features correlate with the development level of a country

In order to resolve relations between country of residence and studied IgG Fc glycan traits, we analyzed correlations between 45 development indicators and 5 derived glycan traits of each analyzed IgG subclass (Supplementary Table 10). Development indicators are standardized statistical measures which quantify the quality of life across nations and communities (Supplementary Tables 11 and 12). Our analysis resulted with 44 statistically significant correlations of IgG Fc monogalactosylation, digalactosylation and agalactosylation with 23 different development indicators. The strongest correlation was observed between IgG1 monogalactosylation and Millennium Development Goals (MDG), Human Development Index (HDI) and stunting. On the other hand, we did not observe any significant correlations between any of the development indicators and sialylation or the incidence of bisecting GlcNAc on any of the IgG subclasses.

**Figure 3:**
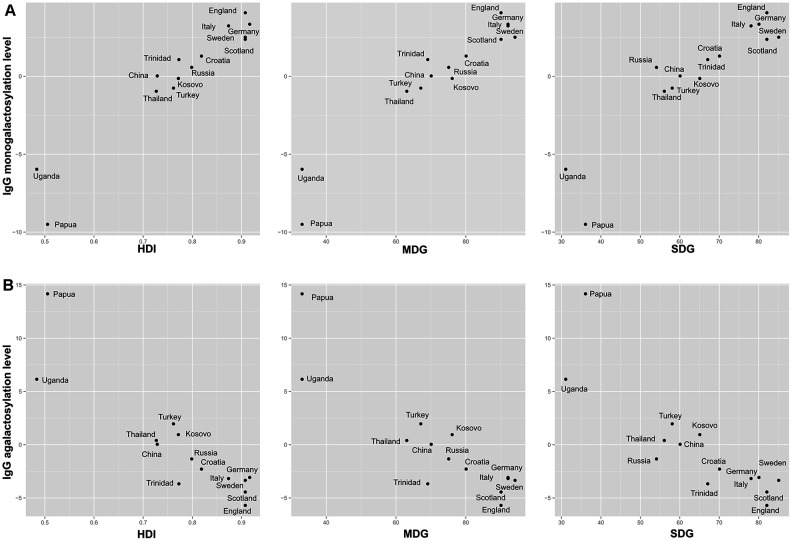
**Relationship between IgG1 Fc galactosylation levels with development indices.** Relationship between IgG1 Fc monogalactosylation (**A**) and relationship between IgG1 Fc agalactosylation (**B**) with United Nations’ development indices for a specific country of residence. HDI = Human Development Index; SDG = health-related Sustainable Development Goals index; MDG = health-related Millennium Development Goals index.

We found a positive correlation between United Nation’s Human development index (HDI) and IgG1 Fc monogalactosylation, while HDI negatively correlated with IgG1 agalactosylation (Figure 3). These findings were replicated for IgG2 subclass as well, where HDI positively correlated with monogalactosylation levels. Therefore, participants from developing countries appear to have lower levels of IgG Fc monogalactosylation and digalactosylation when compared to their counterparts from developed countries.

### IgG Fc monogalactosylation correlates with population’s health status

To determine the relationship between health quality and IgG glycans, correlations between the two were calculated. Population’s health quality was expressed through general health indices (HDI, SDG, MDG) and specific health indicators such as stunting, mortality and life expectancy. Countries with lower development level, in general, had also lower health-related indicators (Supplementary Table 13). The majority of health-related indicators appeared to be correlated with IgG Fc monogalactosylation (Supplementary Table 10). Millennium Development Goals (MDG) index, which describes health-related indicators in MDG system, was positively correlated with IgG1 and IgG2 monogalactosylation (*r*=0.97, *P*=7.44×10^-6^ and *r*=0.86, *P*=4.59×10^-2^ respectively) and negatively correlated with IgG1 agalactosylation (*r*=-0.90, *P*=8.16×10^-3^). In a similar fashion, a positive correlation was observed between IgG1 monogalactosylation and Sustainable Development Goals (SDG) index, non-MDG index (health-related SDG indicators not included in MDG) and Health index, which, like MDG index, display overall health quality of a specific country. SDG index was also negatively correlated with IgG1 agalactosylation (Supplementary Table 10, Figure 3).

Besides general health-related indices, specific health-related indicators also correlated with IgG Fc agalactosylation, monogalactosylation and digalactosylation. Among all studied specific indicators, the decline in stunted growth prevalence demonstrated the strongest positive correlation with IgG1 monogalactosylation (*r*=0.97, *P*=1.16×10^-5^; *n*=14). Among other studied indicators, universal health coverage and the decrease in occupational risk burden displayed substantial correlations with IgG Fc agalactosylation and monogalactosylation levels. Life expectancy is also one of the most important indicators, used to describe life quality. Both female and male life expectancies were correlated with IgG Fc monogalactosylation. Individual’s exposure to various antigens was presented through indicators such as hygiene, water availability, WaSH mortality and sanitation, which also showed significant correlations with IgG Fc galactosylation levels. Of the various infectious diseases, only hepatitis B showed a significant correlation with IgG Fc monogalactosylation (Supplementary Table 10).

Moreover, digalactosylation of IgG1 demonstrated five positive correlations with health-related development indicators, where skilled birth attendance and again, stunted growth, had the strongest associations with this glycan trait (Supplementary Table 10).

Although IgG1 Fc glycans showed the strongest correlation with the health-related indicators, significant correlations between IgG1 Fc galactosylation related glycan features and the socioeconomic indicators, such as education and economic development, have also been identified. Education index was significantly correlated with both Fc monogalactosylation (*r*=0.94, *P*=5.14×10^-4^, *n*=14) and agalactosylation (*r*=-0.90, *P*=1.02×10^-2^, *n*=14), while Gross Domestic Product (GDP) was significantly correlated only with monogalactosylation (*r*=0.89, *P*=1.46×10^-2^, *n*=14; Supplementary Table 10). Correlation analysis between development indicators used in this study displayed high dependencies between used variables (Supplementary Table 14).

## DISCUSSION

This is the largest study to date that has analyzed IgG glycosylation in various populations, encompassing 10,482 of total IgG N-glycomes originating from 5 different populations and 2,579 Fc IgG glycopeptide profiles of subjects from 27 populations. As suggested by previously published data, we observed considerable variation of IgG N-glycan profiles between individuals within the same population, as well as between different populations [23, 38].

Within five populations, where glycans from the whole IgG molecule were analyzed, we observed changes in glycan patterns depending on age and sex. Sex-specific changes are thought to be caused by differences in hormone composition between sexes, especially estrogen that can vary due to pregnancy, menopause or hormonal therapy [23]. This is clearly visible in female N- glycan profiles, where around the age of 50, a decrease of galactosylated IgG glycoforms can be detected. This decrease in galactosylation is associated with alterations and finally decrease of estrogen levels during menopausal transition and post-menopause [39]. Despite the fact that the exact mechanism remains unknown, it has been proposed that high levels of estrogen and progesterone decrease inflammation through reduced production of inflammatory cytokines and induction of helper T lymphocytes [40].

IgG glycosylation is also known to change with chronological and biological age of an individual [28]. We observed an expected decrease in levels of digalactosylation and increase in bisecting GlcNAc levels with the age of participant. Age-related decline in IgG galactosylation is associated with the increase in systemic low-grade inflammation which can be observed in older people [28, 41]. This increase in inflammation is usually explained by higher levels of pro-inflammatory cytokines, such as tumor necrosis factor-α and interleukin-6, found in aged individuals [42]. In accordance, decreased galactosylation also enhances the pro-inflammatory potential of IgG. The decrease in IgG galactosylation has also been observed in premature ageing syndromes, which are also accompanied by inflammation, as in numerous inflammatory and autoimmune diseases, such as inflammatory bowel disease (IBD), rheumatoid arthritis and systemic lupus erythematosus [26, 36, 41, 43]. Although exact mechanisms underlying age-related changes in IgG glycan profile remain unknown, there are several proposed pathways which could explain them. Possible mechanisms include various expression or activity of enzymes involved in glycosylation processes in B-cells and clonal selection of B-cells with specific glycosylation patterns [44, 45]. Therefore, through modulation of inflammation, IgG galactosylation, or more precisely agalactosylation, is proposed to contribute to a process of biological ageing [46].

We have demonstrated differences in glycan profiles between analyzed populations with both analytical approaches – with total IgG N-glycans and Fc glycopeptides, which suggests population-specificity of IgG glycosylation. The highest differences in Fc glycopeptides between analyzed countries were observed in their galactosylation levels. Furthermore, we found that the indices describing country’s development level, expected lifespan and numerous health related indicators were positively correlated with IgG galactosylation levels, especially with monogalactosylation. We have also observed significant correlations between IgG Fc galactosylation-related glycan traits and the socioeconomic indicators. Economy and education quality are tightly connected with the development level and quality of the healthcare system [47]. As a matter of fact, all analyzed development indicators appeared to be highly co-dependent, making it impossible to pinpoint specific indicator associated to a distinct galactosylation change (Supplementary Table 14). It is known that besides genetics, environment also plays a crucial role in the regulation of IgG glycosylation [25, 36]. Pathogens, stress, certain medications and nutrition are the most probable players orchestrating non-genomic component of IgG glycosylation [24, 48, 49]. Low galactosylation levels suggest higher IgG inflammatory potential in some of the analyzed populations. Therefore, it would be interesting to check if participants from populations with lower IgG galactosylation had an underlying inflammatory condition at the time of sampling or if measured IgG galactosylation level represents a baseline for the studied population. However, clinical data on inflammation markers were not available for this study, warranting further research with improved study design to resolve the aforementioned predicament. Nevertheless, it was reassuring to see that our results replicate previous findings in a recent study on 773 children from Gabon, Ghana, Ecuador, the Netherlands and Germany, where the increase in agalactosylated species was observed in individuals from Gabon, Ghana and Ecuador, compared to participants from the Netherlands and Germany. The study suggested that higher exposures to antigens in developing countries may drive pro-inflammatory IgG glycan profile and activate immune system against pathogens [38]. Significantly lower IgG Fc galactosylation levels were also observed in two African populations in comparison to US participants, suggesting associations with inflammatory glycosylation as well [50].

As already mentioned, IgG glycosylation is responsive to various environmental factors, including nutrition and use of certain medications. For instance, dietary habits vary substantially between countries, especially if we compare countries on different continents. Various diets in different regions could also be a possible source of variation in glycan profiles. It was demonstrated in mouse models that high fat diet alters IgG glycosylation, consequently activating signaling pathways that induce insulin resistance and hypertension, directly impacting cardiometabolic health [51, 52]. Additionally, use of certain medications, such as immune modifying analgesics, causes changes in immune activation [53]. The use and availability of these drugs differs significantly between the countries and could potentially represent a confounding factor to the observed changes in IgG galactosylation levels between the studied populations. However, clinical data on the use of immunomodulating drugs at the time of sampling were not available, disabling further investigation of this matter.

Our study shows differences in glycan profiles between analyzed cohorts and intriguing associations with various country development indicators, but has several limitations. Although most of our cohorts are representative of their base populations, we cannot exclude the possible existence of sampling bias in some cohorts, due to inconsistencies in inclusion criteria and lack of information on sampling methods. Example of this limitation is a cohort of Thailand sex-workers, where participants with a high risk of HIV infection were enrolled. Although all included participants were HIV negative, the group included sex workers and hepatitis positive individuals and therefore in not a true representative of Thailand’s base population. We did not observe substantial differences in monogalactosylation levels for this cohort when compared to other cohorts in region (China Han). However, further studies are needed to properly check if there are differences in glycan profiles between a base population and a population with high risk of HIV infections in Thailand. Additionally, although European countries are well represented, the rest of the world is still underrepresented. For example, Uganda is the only population from African continent and this representation of genetical, socio-economical, cultural and behavioral differences cannot be generalized to the whole continent.

Additional studies with adequate number of representative participants from each of the included base populations are needed to obtain baseline populational IgG glycosylation profiles. To tackle the source of variation in glycan profiles in various populations, information on participant’s health status, detailed medical history and biochemical markers would have to be collated. Unfortunately, sensibility of existing analytical methods to experimental variation and cofounding effect of age on glycan profile limits the availability of adequate sample sets for such analysis. Nevertheless, possible skewness of cohort participants in some populations should not affect and invalidate presented results and conclusions.

In summary, this observational cohort study revealed that immunoglobulin G glycosylation patterns vary within and between different populations. In addition to corroborating the previous findings on age- and sex-related IgG glycosylation changes, we also observed associations related to participants' country of residence. Moreover, certain IgG patterns could be associated with pronounced inflammatory potential in some populations. We also obtained intriguing correlations between IgG galactosylation and various development indicators describing populational health, education and income, leaving cause of these changes as an open question to be answered by further studies.

## MATERIALS AND METHODS

### Study participants

Total IgG glycome analysis was based on 10,482 human participants from China [36, 54], Croatia [55], Estonia and two cohorts from the United Kingdom (population of Scotland from Orkney Islands [23] and England from the TwinsUK cohort [56]; Supplementary Table 1).

Subclass specific analysis of IgG Fc glycosylation included 2,579 individuals. Volunteers originated from 14 different countries and 25 different ethnic groups (Supplementary Table 6). For Kazak and English cohorts, we had two populations obtained from different medical centers. In general, inclusion criteria required participants not to have any physiological or pathological conditions that are known to affect IgG glycosylation profile. Detailed descriptions of analyzed populations are below:

### Trinidad and Tobago

Controls from the Latin American Genoma de Lupus Eritematoso Sistemico Network (GENLES) study were selected for analysis. Controls were selected from the same underlying population as the SLE cases of African/European genetic admixture. For each SLE case, two controls, matched for sex and for 20-year age group, were randomly chosen from the neighborhood [36]. Sampling may underrepresent predominantly Chinese and Indian populations, but 5-way admixture in samples (African, European, Indian, Chinese, Native American) reflects the diverse ancestry.

### Han Chinese; Kazak, Kyrgyz and Uyghur minorities

Han Chinese samples were collected in Beijing (northern China) and Tangshan (eastern China) to represent the majority of Han Chinese.

Kazak, Kyrgyz and Uyghur participants were recruited from Xinjiang Autonomous Region to represent the Chinese minority ethnics. Kyrgyz samples originated from Halajun Town, Atushi City, Kizilsu Kirghiz Autonomous Prefecture. Kazak samples originated from Qapqal county, Ili Kazak Autonomous Prefecture. Uyghur samples originated from Minfeng County, Hotan Prefecture. All the participants had to meet the following inclusion criteria: signed informed consents before participation, aged more than 18 years, self-reported Kyrgyz / Kazakh/ Uyghur ethnicity without intermarriage history with other ethnic groups within at least the past three generations and no documented clinical diagnosis of specific diseases. Individuals who met the diagnostic criteria for the specific cardiovascular, respiratory, genitourinary, gastrointestinal or hematological disease were excluded from the study [29, 34, 57, 58].

### India, England and Jamaica

Representatives for India, England and Jamaica were selected from a SABRE study. First-generation migrants from India and Jamaica were initially recruited as a population-based sample aged 40–70 years and randomly selected from ethnicity and sex-stratified primary care practitioners’ lists. Only participants with no coronary heart disease, stroke and diabetes were selected for analysis. This population has the same health status as the older general population in the UK. Ethnicity was described by the interviewer based on appearance and parental origin [59].

### Roma population

The Croatian Roma samples used in this study were selected from the database collected during multidisciplinary anthropological and epidemiological community-based investigations of adult Roma individuals living in Croatia. The collected samples represent a general population of adult Roma living in Croatia. The subsample presented in this cohort is created in order to equally represent both sexes and both main linguistic subgroups of the Roma: Bayash (Vlax) and Balkan Roma. Furthermore, this sample equalizes the number of individuals in 10-years age groups that are approximately the same in men and women, and in the Bayash and Balkan Roma [60].

### Sweden

Participants were blood donors which were recruited within the Örebro region (the primary catchment area of the Örebro University Hospital). In addition to the normal requirements for blood donors, i.e. age 18–60 years, not being diagnosed with any transmittable disease or chronic disease that may impact on the composition of the blood, not having done any tattoo/piercing within the last 6 months, not undergone surgery within the last 1-6 months (depending on type of surgery) and not having any ongoing infections. Any chronic gastrointestinal symptoms were used as an exclusion criterion. The age profile of the inhabitants in the region is like that of Sweden.

### Thailand

Participants were recruited from bars, clubs, and other locations associated with transactional sex. Men and women, 18 to 50 years of age, who were at high risk for HIV-1 infection were identified with the use of an audio computer-assisted self-interview. To be eligible for study entry, participants had to meet at least one of the following four criteria within the previous 3 months: had exchanged goods for sex, had unprotected sex with a known HIV-positive partner, had unprotected sex with three or more partners, and had symptoms of a sexually transmitted infection. Participants who were HIV -negative were analyzed in this study [61].

### Uganda

Residents of Kayunga District, Uganda aged 15 to 49 years were enrolled. Contact information was obtained, a blood sample collected, and a questionnaire administered. Participants also provided a medical history and received a physical examination that included observations for weight, temperature, blood pressure, pulse and presence of lymphadenopathy. A blood sample was then obtained for HIV-1 testing. Samples from HIV-negative participants were included in this study [62].

### Evenks

Evenki samples were studied for genetic markers and biochemical traits. They are representatives of the Evenki populations of Siberia. The people studied were healthy as that population is and have minimum modern medical care.

### Yakuts

Yakut samples were part of genetic markers study of horse ranchers of Mongolian origin. They are a more recent migrant population to Siberia.

### Papua New Guinea

The Watut population, which is a representative of the transitional Highland populations of New Guinea, was a part of a Tropical Splenomegaly syndrome study. Analyzed samples in this study were controls that did not have the disease.

### Slavs, Kazakhs, Tatars and Yakuts

Slavs, Kazakhs, Tatars and Yakuts were collected as part of local projects for population genetic studies. The goal was to collect conditionally healthy population controls.

Slavs were blood donors who have clinically shown no infectious and chronic diseases selected for Slavic ethnic origin. Ethnicity was evaluated in the interview process, as well as visually where people with Asian features were excluded.

Kazakhs were collected in National Center for Biotechnology, Astana, Kazakhstan. It is a conditionally healthy population control group to evaluate HLA alleles distribution in Kazakhs. Ethnicity was evaluated in the interview process.

Tatars were collected at Kazan Federal University, Kazan, Republic of Tatarstan for forensic purposes. Blood donors have clinically shown no infectious and chronic diseases. Ethnicity was evaluated in the interview process.

Yakut samples were collected in the Institute of Health, North-Eastern Federal University, Yakutsk, Russia. Samples were collected in the areas of native habitats of the Yakuts containing a pure ethnic group.

### Orkney and Shetland islanders

Participants were selected among controls for patients with multiple sclerosis (MS) living on the islands or in the Grampian or Highland regions of mainland Scotland were identified by contacting general practices on the islands and reviewing MS databases held in secondary care in Aberdeen, Inverness, Orkney and Shetland. Recruitment of cases to the study was conducted through letters forwarded by general practitioners inviting those of Orcadian or Shetlandic descent to participate. [63].

### TwinsUK population

The UK's largest registry of adult twins, or TwinsUK Registry, encompasses about 12,000 volunteer twins from all over the United Kingdom. More than 70 % of the registered twins have filled at least one detailed health questionnaire and about half of them have undergone a baseline comprehensive assessment and two follow-up clinical evaluations [56].

### Croatia

The participants originated from the City of Split (2012-2013). The participants were recruited in the study following general practitioner’s advice, newspaper and radio announcements, or distribution of posters and leaflets. In order to participate, the participants had to be of age (18 or more years) and had to sign the informed consent prior to the enrolment [64].

### Germany

Samples representing Germany were collected in a genetic epidemiological research, based on the KORA platform (Cooperative Health Research in the Region of Augsburg). Biosamples and phenotypic characteristics, as well as environmental parameters of 18,000 adults from Augsburg and the surrounding counties are available [49].

### Kosovo

Samples were collected from Kosovars of Albanian ethnicity in the area of Podujevo city and neighboring villages.

### Italy

Used samples were a subgroup of 427 controls for IBD glycome project that did not have the disease. Participants were enrolled at Careggi University Hospital in Florence, Italy [26].

### Turkey

Samples of healthy participants were collected at Koc University, Istanbul, Turkey.

Samples were randomized across 96-well plates (31 in total), with five technical replicates of a standard sample and one blank, serving as a negative control. The development level of a country was assessed using three development indices: health-related Sustainable Development Goal index (SDG) [65], health-related Millennium Development Goals index (MDG) and United Nation Human Development Index (HDI) which is a summary measure of the development level of a certain country [65, 66] HDI represents three dimensions of life: economy, education and health quality. Specific aspects of human life were assessed using other development indicators (Supplementary Table 10). Blocking was performed by equally distributing subjects of the same sex and similar age from all the cohorts across used plates. Plasma samples used as standards were obtained from Croatian National Institute of Transfusion Medicine. The study was performed in compliance with the Helsinki declaration and all participants gave written informed consent. Ethical approvals were obtained by relevant ethics committees.

### Immunoglobulin G isolation

For all samples, the initial material for IgG isolation was human blood plasma. Protein G affinity chromatography was used to isolate immunoglobulin G from plasma as described previously [23]. In short, the maximum volume of 100 μL of human peripheral blood plasma or serum was diluted with 1X phosphate buffer saline (PBS) and loaded onto protein G monolithic plate (BIA Separations, Ajovščina, Slovenia). Samples were washed three times with 1X PBS and IgG was eluted using 0.1M formic acid (Merck, Darmstadt, Germany) followed by immediate neutralization with 1M ammonium bicarbonate (Acros Organics, Pittsburgh, PA).

### Immunoglobulin G trypsin digestion and purification

Subclass specific analysis of IgG Fc glycosylation included 2,579 in dividuals from 14 different countries and 25 different ethnic groups (Supplementary Table 6). We studied glycopeptides from three IgG sublessees; IgG1, IgG2 and IgG4 and each subclass was studied for various glycan features (Supplementary Tables 15, 16).

IgG glycopeptides were obtained and purified as described before [49]. Approximately 15 μg of isolated IgG was treated with 0.1 μg of sequencing grade trypsin (Promega, Fitchburg, WI) and incubated overnight at 37 °C. The reaction was stopped by dilution with 0.1 % trifluoroacetic acid (TFA; Sigma-Aldrich, St. Louis, MI). Glycopeptides were purified using a solid-phase extraction on Chromabond C-18 sorbent (Macherey-Nagel, Düren, Germany). Samples were loaded onto beads in 0.1 % TFA and washed three times using the same solvent. Glycopeptides were eluted from the phase with 20 % LC-MS grade acetonitrile (ACN; Honeywell, Morris Plains, NJ). Eluted glycopeptides were vacuum-dried and reconstituted in 20 μL of ultrapure water prior to LC-MS analysis. All glycan analyses were performed at Genos laboratory.

### Release and labelling of the total IgG N-glycans

Total IgG glycome was analyzed in participants from China [36, 54], Croatia [55], Estonia, Orkney Islands [23] and the TwinsUK cohort [56] (Supplementary Table 1). Glycan release and labelling of Croatian samples were performed as previously described [28]. Briefly, IgG was incorporated into sodium dodecyl sulphate polyacrylamide gel and glycans were released from protein using an overnight incubation with PNGase F (ProZyme, Hayward, CA). Released glycans were labelled with 2-aminobenzamide (2-AB; Sigma-Aldrich) and purified on Whatman 3 mm chromatography paper. For cohorts from Scotland, England, China and Estonia, glycans were released as previously described [28, 48]. Briefly, IgG was denatured using 1.33 % (w/v) sodium dodecyl sulphate (Invitrogen, Carlsbad, CA) and samples were incubated at 65 °C for 10 minutes. Subsequently, 4 % (v/v) Igepal CA-630 (Sigma–Aldrich) and 1.25 mU of PNGase F (ProZyme) were added to each sample and incubated overnight at 37 °C. For glycan labelling, 48 mg/mL of 2-AB in dimethyl sulfoxide (Sigma–Aldrich) and glacial acetic acid (Merck) (v/v 85:15) was mixed with reducing agent (106.96 mg/mL of 2-picoline borane (Sigma–Aldrich) in dimethyl sulfoxide). Labelling mixture was added to samples, followed by 2-hour incubation at 65 °C.

After incubation, Estonian and Chinese samples were brought to 96 % ACN (J.T. Baker, Phillipsburg, NJ) and applied to each well of a 0.2 μm GHP filter plate (Pall Corporation, Ann Arbor, MI). Samples were subsequently washed five times using acetonitrile/water (96:4, v/v). Glycans were eluted with water and stored at -20 °C until usage. Samples from England and Scotland were purified using a solid-phase extraction on 200 μL of 0.1 g/L microcrystalline cellulose suspension (Merck) in a 0.45 μm GHP filter plate (Pall Corporation). Deglycosylation reaction was diluted four times with ACN loaded to cellulose. Samples were washed three times with 80 % ACN and eluted with ultrapure water.

### HILIC-UPLC analysis of fluorescently labelled N-glycans

Fluorescently labelled N-glycans were separated by hydrophilic interaction liquid chromatography (HILIC) on a Waters Acquity UPLC H-class instrument (Waters, Milford, MA) equipped with FLR fluorescence detector set to 330 nm for excitation and 420 nm for emission wavelength. Separation was achieved on a Waters bridged ethylene hybrid (BEH) Glycan chromatography column, 100 × 2.1 mm i.d., 1.7 μm BEH particles with 100 mM ammonium formate (pH 4.4) as a solvent A and ACN as a solvent B. Separation method used linear gradient from 75 % to 62 % solvent B (v/v) at a flow rate of 0.4 mL/min in a 25-minute analytical run. Column temperature was maintained at 60 °C. Obtained chromatograms were manually separated into 24 peaks using Empower 3 software, from which, using the total area normalization, relative abundances of 24 directly measured glycan traits were obtained (Supplementary Table 2). In-depth characterization of each of 24 chromatographic peaks was performed as previously described [23]. The most abundant glycan structure in each peak was chosen to represent that glycan peak. An example of chromatogram integration with the most abundant glycan structures in each peak of IgG glycome is shown in Supplementary Figure 1.

### LC-MS analysis of IgG Fc glycopeptides

Trypsin-digested, subclass-specific glycopeptides were separated and measured on nanoAcquity chromatographic system (Waters, Milford, MA) coupled to Compact mass spectrometer (Bruker, Bremen, Germany), equipped with Apollo II source as described previously with minor changes [67]. Samples (9 μL) were loaded onto PepMap 100 C8 trap column (5 mm × 300 μm i.d.; Thermo Fisher Scientific, Waltham, MA) at a flow rate of 40 μL/min of solvent A (0.1 % TFA) and washed of salts and impurities for one minute. Subclass-specific glycopeptides were separated on C18 analytical column (150 mm × 100 μm i.d., 100 Å; Advanced Materials Technology, Wilmington, DE) in a gradient from 18 % to 25 % of solvent B (80 % ACN) in solvent A. Column temperature was set to 30 °C and flow rate was 1 μl/min. NanoAcquity was coupled to mass spectrometer via capillary electrophoresis sprayer interface (Agilent, Santa Clara, CA), which allows mixing of analytical flow with sheath liquid (50 % isopropanol, 20 % propionic acid; Honeywell, Morris Plains, NJ).

Mass spectrometer was operated in a positive ion mode, with capillary voltage set to 4500 V, nebulizer pressure set to 0.4 bar and drying gas set to 4 L/min at 180 °C. Spectra were recorded in a *m/z* range of 600 - 1800. Collision energy was 4 eV.

IgG glycopeptides were confirmed by tandem mass spectrometry (MS/MS) analysis of a pool of 90 randomly chosen samples. MS/MS analysis was performed on Compact instrument using CaptiveSpray interface. Gaseous acetonitrile was introduced into nitrogen flow using nanoBooster. Capillary voltage was set to 1500 V with nitrogen pressure set to 0.2 bar and a temperature of 150 °C. AutoMS/MS method was used with selection of three precursor ions and exclusion criteria after one MS/MS spectrum. Mass range was set from 150 *m/z* to 3400 *m/z* and spectra rate of 1 Hz. Transfer time was set to 100 μs and pre-pulse storage was 12 μs. Used separation method was the same as for the analyzed samples, except there was no sheathing-liquid flow applied to the source. Fragment spectra were manually searched for diagnostic peptide y-ion series and glycopeptide fragments specific for IgG glycopeptides (Supplementary Figures 3–5).

Obtained raw data was converted to centroid mzXML files using ProteoWizard version 3.0.1. software. Samples were internally calibrated using a defined list of IgG glycopeptides with highest signal-to-noise ratios and required isotopic patterns. After calibration, signals matching IgG Fc glycopeptides were extracted from data using 10 *m/z* extraction window. First four isotopic peaks of doubly and triply charged signals, belonging to the same glycopeptide species, were summed together, resulting in 20 glycopeptides per IgG subclass. Predominant allotype variant of IgG3 tryptic peptide carrying N-glycans in Caucasian population has the same amino acid sequence as IgG2. On the other hand, in Asian and African populations predominant variant of the same peptide has the same amino acid composition as IgG4 making the separation of IgG3 from other subclasses impossible using given separation methods [68]. Therefore, IgG glycopeptides were separated into three chromatographic peaks labelled IgG1, IgG2 and IgG4. Signals of interest were normalized to the total area of each IgG subclass.

### Statistical analysis

Data analysis was performed using program R, version 3.0.1. with a ggplot2 package for creation of visualizations. Since obtained globally normalized abundances of glycan structures show the right-skewness of their distributions, data were log-transformed. To remove experimental and batch biases, all measurements were batch-corrected using ComBat R package. Derived glycan traits representing levels of galactosylation (agalactosylation, monogalactosylation and digalactosylation), sialylation, core fucosylation and incidence of bisecting GlcNAc were calculated from obtained data as described before [23, 49]. Derived glycan traits represent a portion of structurally similar glycan species which share common biosynthetic pathways. Level of IgG glycans containing galactose was represented by agalactosylation (no galactoses), monogalactosylation (one galactose) or digalactosylation (two galactoses attached to antenna). In short, total IgG derived glycan traits were calculated as portion of glycans (%) containing common structural features (e.g. number of galactoses) in a total IgG glycome (Supplementary Table 3). In case of subclass specific IgG Fc glycopeptide analysis, derived glycan traits were calculated as a portion of glycopeptides containing common structural features within a specific IgG subclass (Supplementary Table 16). Correlations between derived glycan traits are defined in Supplementary Tables 17 and 18. Core fucosylation was excluded from IgG Fc specific glycopeptide analysis due to low data quality of non-fucosylated species.

Linear mixed model was used to analyze associations between glycan traits and the subject’s country of residence (R package “lmer”). Analysis was performed using linear mixed model framework since it allows to explicitly model the hierarchical structure of our data (geographical clustering of measured samples). In the model, sex and age were described as fixed effects, while the country of residence was described as a random effect. For each variable of interest (country of residence, age and sex), *R*^2^ (variance explained) was calculated as described in Nakagawa et al. [69]. The likelihood ratio test was used to determine the significance of country of residence variability in glycan trait variability. Pearson’s correlation coefficient was used to express relationships between country-specific development indicators and levels of glycan traits in participants from the same country. *P* values were adjusted for multiple testing using Bonferroni correction.

### Data availability

The data that support the findings of this study are available from the corresponding author upon reasonable request. Extracted raw data from LCMS analysis is available on https://www.synapse.org/ under ID syn21559596.

## Supplementary Material

Supplementary Figures

Supplementary Tables 1-6

Supplementary Table 7

Supplementary Table 8

Supplementary Table 9

Supplementary Table 10

Supplementary Tables 11-13

Supplementary Table 14

Supplementary Tables 15-18
