# Supplementary material for: Global variability of the human IgG glycome
Source: Aging (Albany NY). 2020 Aug 12;12(15):15222–59. doi: 10.18632/aging.103884 (PMC7467356; doi:10.18632/aging.103884)
Supplement: Supplementary Figures [file aging-12-103884-s009..pdf]

SUPPLEMENTARY FIGURES

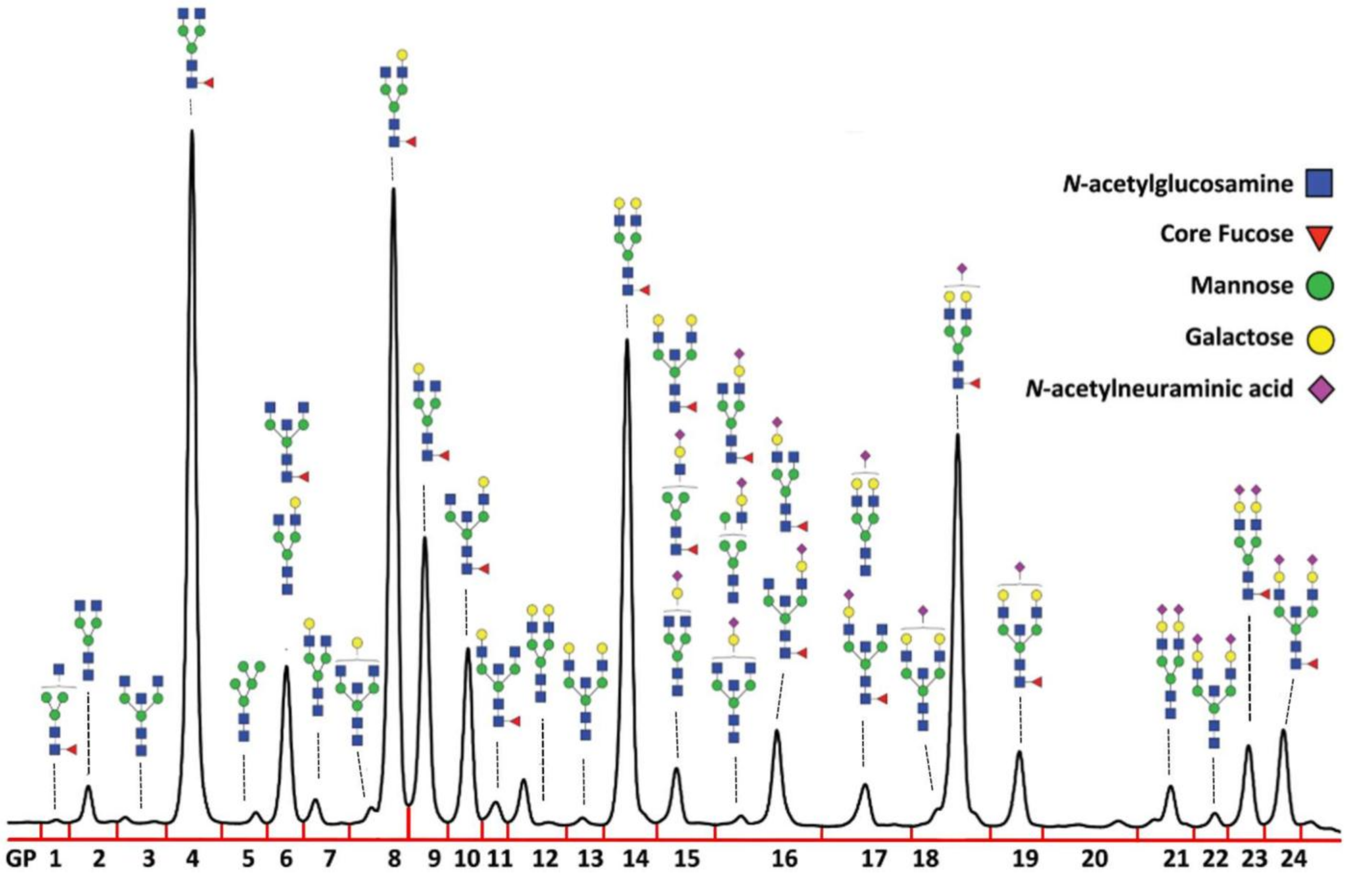

Supplementary Figure 1. HILIC-UPLC chromatogram of total IgG released glycans labelled with 2-AB. Dominant structures are indicated above each of 24 glycan peaks (GP 1 - 24).

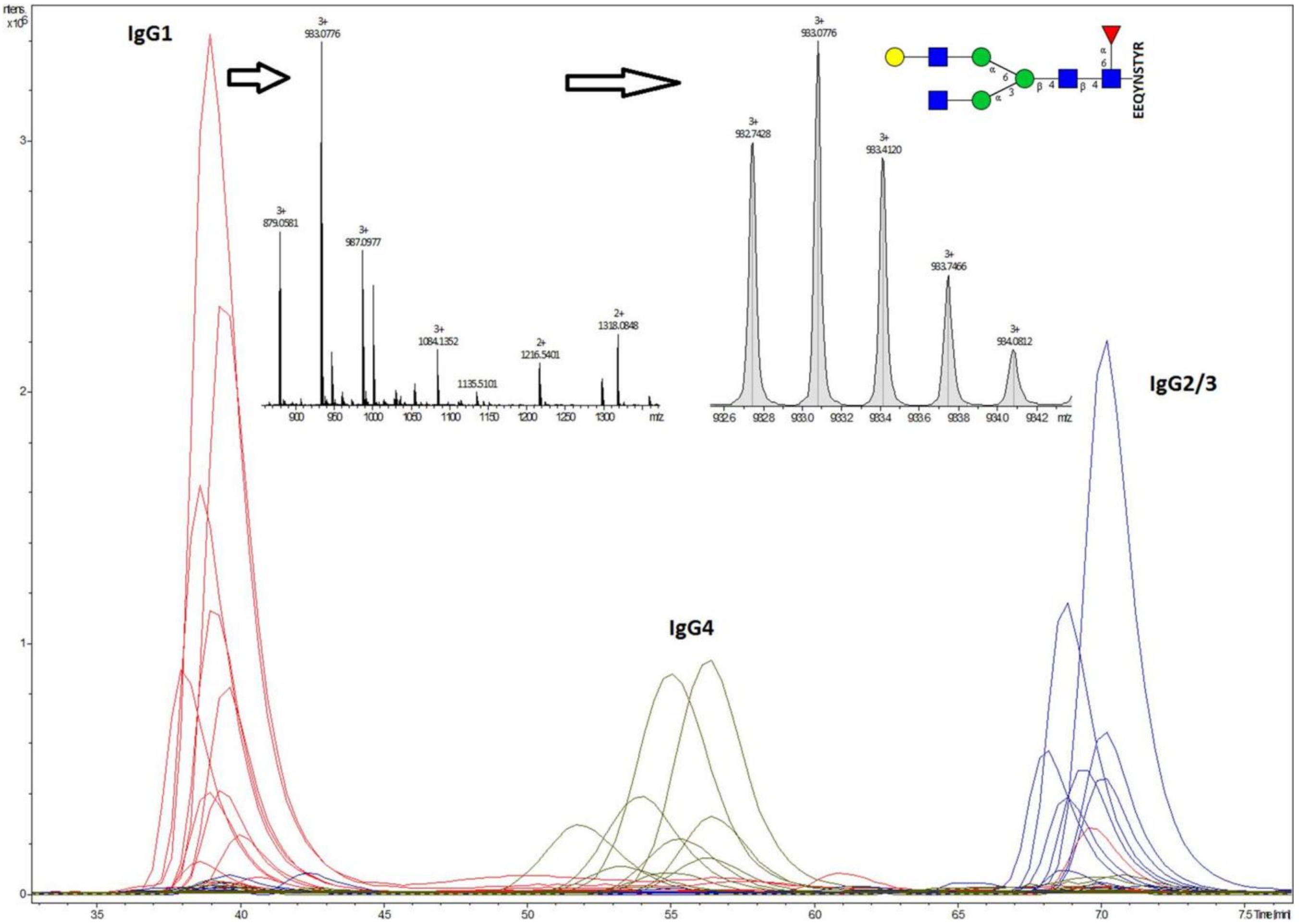

Supplementary Figure 2. Extracted ion chromatograms of IgG Fc glycopeptides. IgG1 subclass is shown in red, IgG4 in green and IgG2 in blue. Mass spectra containing masses that correspond to IgG1 glycopeptides and isotopic distribution of the most abundant glycopeptide (G1F) are shown.

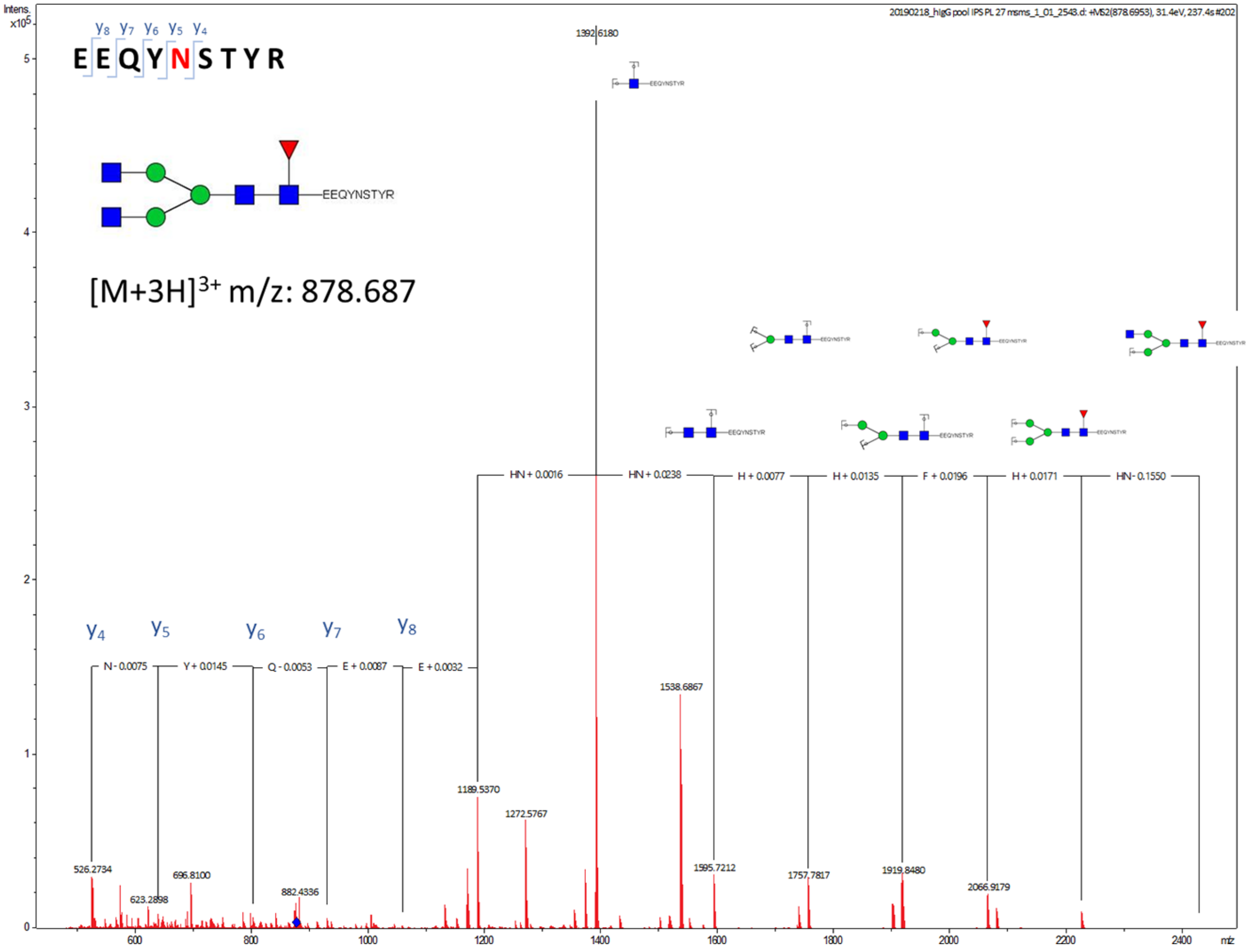

Supplementary Figure 3. Fragment spectra of G0F glycan attached to IgG1 tryptic fragment. Glycan composition: N (N-acetylglucosamine), F (fucose), G (galactose) and S (N-acetylneuraminic acid) followed by a number represents number and type of monosaccharides attached to A2 glycan. Glycan structures are drawn in GlycoWorkbench version 2. Blue square = N-acetylglucosamine, red triangle = fucose, green circle = mannose, yellow circle = galactose, purple diamond = N-acetylneuraminic acid. IgG1 tryptic peptide sequence carrying glycan: E293EQYNSTYR301.

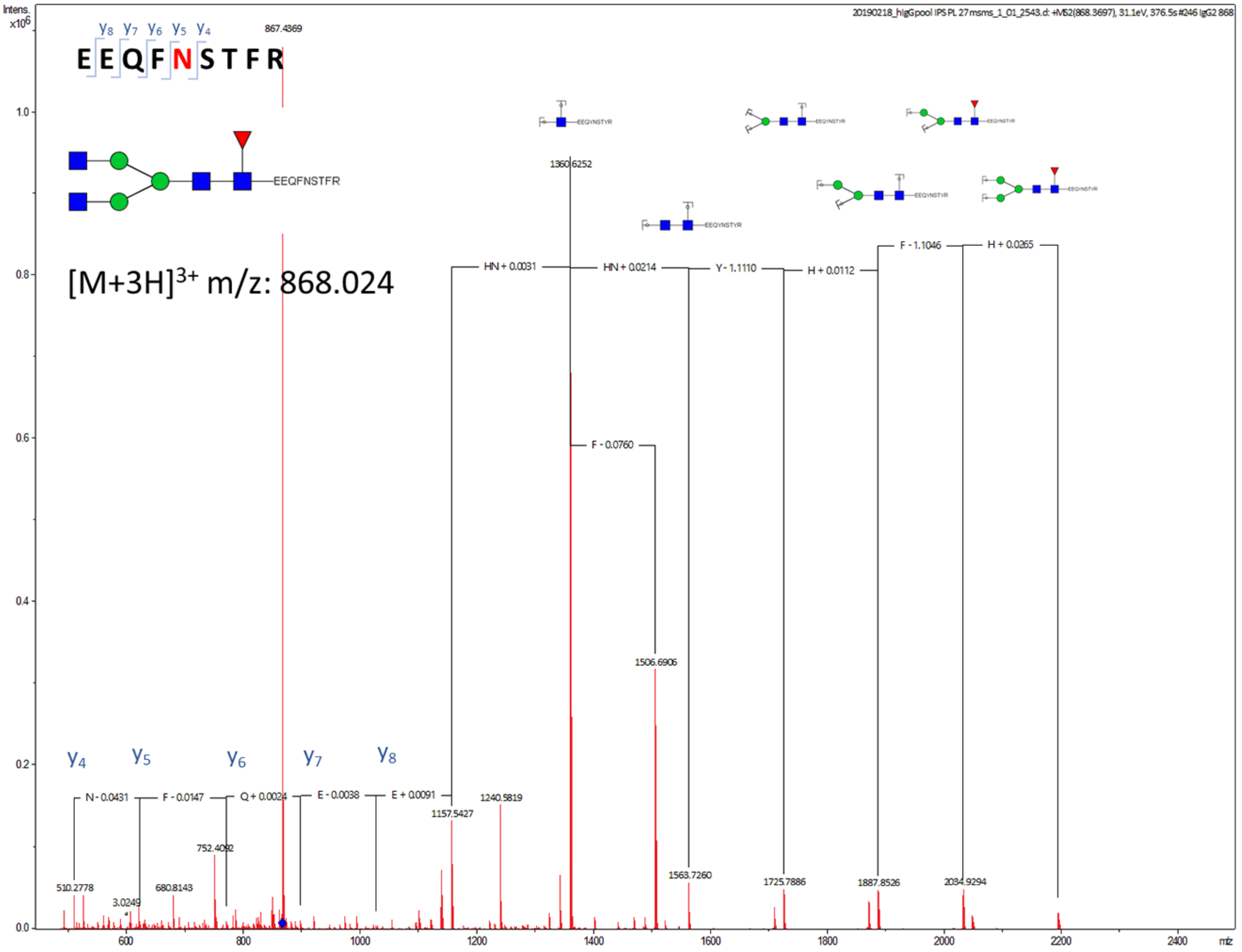

Supplementary Figure 4. Fragment spectra of G0F glycan attached to IgG2&3 tryptic fragment. Glycan composition: N (N-acetylglucosamine), F (fucose), G (galactose) and S (N-acetylneuraminic acid) followed by a number represents number and type of monosaccharides attached to A2 glycan. Glycan structures are drawn in GlycoWorkbench version 2. Blue square = N-acetylglucosamine, red triangle = fucose, green circle = mannose, yellow circle = galactose, purple diamond = N-acetylneuraminic acid. IgG2&3 tryptic peptide sequence carrying glycan: E293EQFNSTYR301.

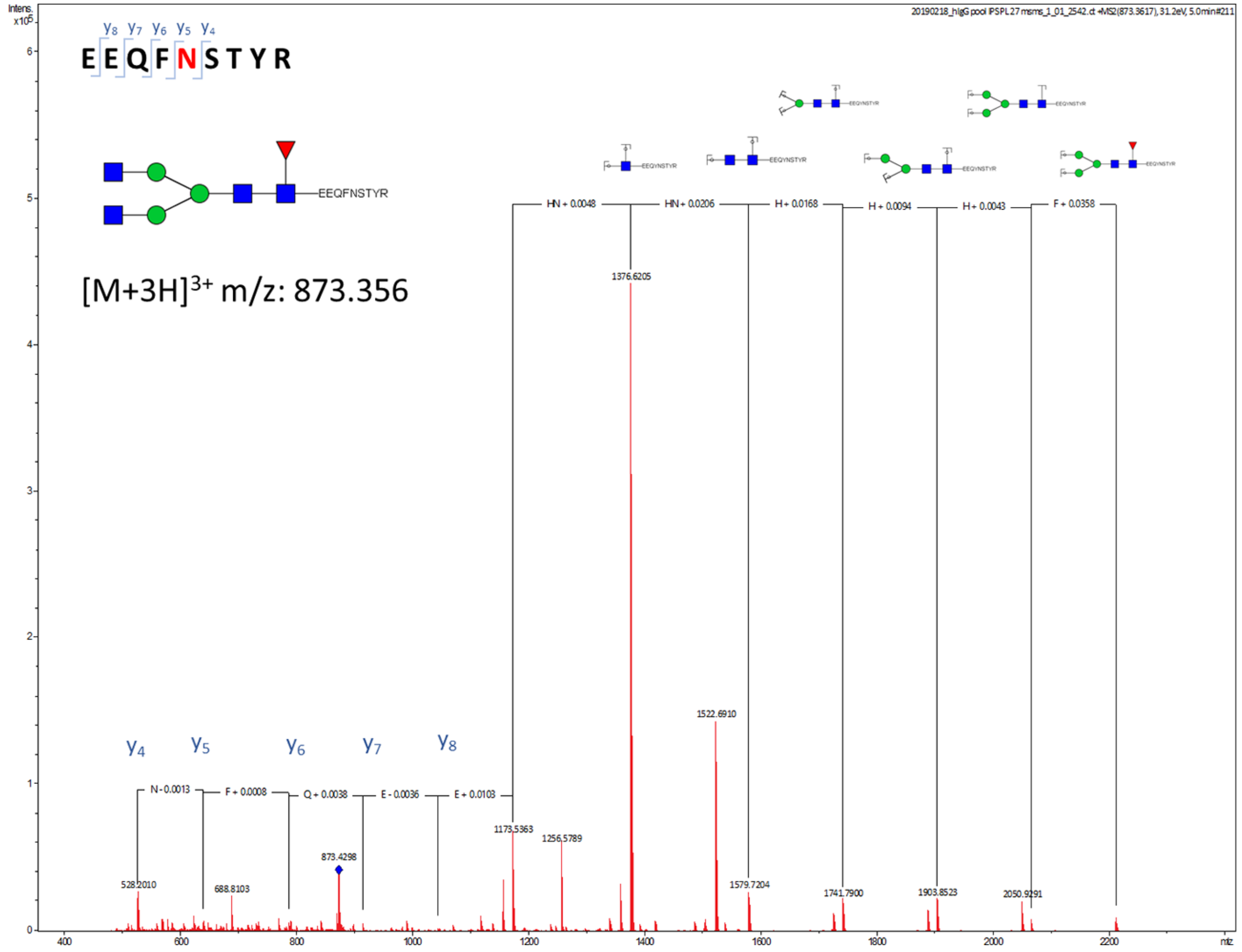

Supplementary Figure 5. Fragment spectra of G0F glycan attached to IgG4 tryptic fragment. Glycan composition: N (N-acetylglucosamine), F (fucose), G (galactose) and S (N-acetylneuraminic acid) followed by a number represents number and type of monosaccharides attached to A2 glycan. Glycan structures are drawn in GlycoWorkbench version 2. Blue square = N-acetylglucosamine, red triangle = fucose, green circle = mannose, yellow circle = galactose, purple diamond = N-acetylneuraminic acid. IgG4 tryptic peptide sequence carrying glycan: E293EQFNSTFR301.
