# Supplementary material for: Global variability of the human IgG glycome
Source: Aging (Albany NY). 2020 Aug 12;12(15):15222–59. doi: 10.18632/aging.103884 (PMC7467356; doi:10.18632/aging.103884)
Supplement: Supplementary Tables 1-6 [file aging-12-103884-s007..pdf]

SUPPLEMENTARY TABLES

Supplementary Table 1. Demographic characteristics of five populations used for HILIC-UPLC analysis of total IgG glycosylation.

Population	Abbreviation	Country of residence	Min age	Q1	Median	Mean	Q3	Max age	F	M
General population of China (Han)	China	China	20	43	48	47	51	68	423	201
General population of Croatia	Croatia	Croatia	18	46	57	56	68	98	1116	689
General population of Estonia	Estonia	Estonia	31	60	69	67	75	88	516	593
Population of Orkney Islands	Scotland	Scotland	17	41	54	53	65	100	1236	794
General population of England (TwinsUK cohort)	England	England	17	42	54	52	62	83	4282	342

In table are given: abbreviation of analysed population, country of participant's residence, age parameters (minimum, maximum, median, mean, 1st and 3rd quartile) and sex parameters (number of female and male participants).

Supplementary Table 2. Description of directly measured traits measured by HILIC-UPLC.

Glycan peak	The most abundant glycan structure[1]	Description of glycan trait	Trait calculation[2]
GP1	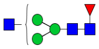	*The percentage of FA1 glycan in total IgG glycans*	*GP1 / TOTAL GLYCANS* 100*
GP2	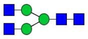	*The percentage of A2 glycan in total IgG glycans*	*GP2 / TOTAL GLYCANS* 100*
GP3	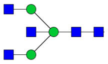	*The percentage of A2B glycan in total IgG glycans*	*GP3 / TOTAL GLYCANS* 100*
GP4	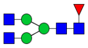	*The percentage of FA2 glycan in total IgG glycans*	*GP4 / TOTAL GLYCANS* 100*
GP5	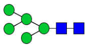	*The percentage of M5 glycan in total IgG glycans*	*GP5 / TOTAL GLYCANS* 100*
GP6	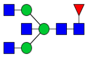	*The percentage of FA2B glycan in total IgG glycans*	*GP6 / TOTAL GLYCANS* 100*
GP7	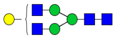	*The percentage of A2G1 glycan in total IgG glycans*	*GP7 / TOTAL GLYCANS* 100*
GP8	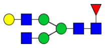	*The percentage of FA2[6]G1 glycan in total IgG glycans*	*GP8 / TOTAL GLYCANS* 100*
GP9	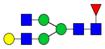	*The percentage of FA2[3]G1 glycan in total IgG glycans*	*GP9 / TOTAL GLYCANS* 100*
GP10	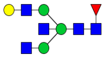	*The percentage of FA2[6]BG1 glycan in total IgG glycans*	*GP10 / TOTAL GLYCANS* 100*
GP11	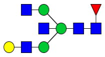	*The percentage of FA2[3]BG1 glycan in total IgG glycans*	*GP11 / TOTAL GLYCANS* 100*
GP12	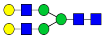	*The percentage of A2G2 glycan in total IgG glycans*	*GP12 / TOTAL GLYCANS* 100*
GP13	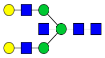	*The percentage of A2BG2 glycan in total IgG glycans*	*GP13 / TOTAL GLYCANS* 100*
GP14	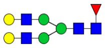	*The percentage of FA2G2 glycan in total IgG glycans*	*GP14 / TOTAL GLYCANS* 100*
GP15	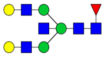	*The percentage of FA2BG2 glycan in total IgG glycans*	*GP15 / TOTAL GLYCANS* 100*
GP16	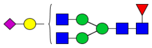	*The percentage of FA2G1S1 glycan in total IgG glycans*	*GP16 / TOTAL GLYCANS* 100*
GP17	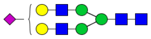	*The percentage of A2G2S1 glycan in total IgG glycans*	*GP17/ TOTAL GLYCANS* 100*
GP18	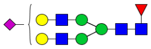	*The percentage of FA2G2S1 glycan in total IgG glycans*	*GP18 / TOTAL GLYCANS* 100*
GP19	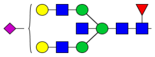	*The percentage of FA2BG2S1 glycan in total IgG glycans*	*GP19 / TOTAL GLYCANS* 100*
GP20		*Structure not determined*	*GP20 / TOTAL GLYCANS* 100*
GP21	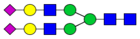	*The percentage of A2G2S2 glycan in total IgG glycans*	*GP21 / TOTAL GLYCANS* 100*
GP22	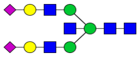	*The percentage of A2BG2S2 glycan in total IgG glycans*	*GP22 / TOTAL GLYCANS* 100*
GP23	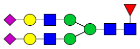	*The percentage of FA2G2S2 glycan in total IgG glycans*	*GP23 / TOTAL GLYCANS* 100*
GP24	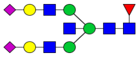	*The percentage of FA2BG2S2 glycan in total IgG glycans*	*GP24 / TOTAL GLYCANS* 100*

Supplementary Table 3. Description of derived IgG glycan traits measured by HILIC-UPLC with correlations between derived glycan traits.

Derived glycan trait	Derived trait description	Derived trait calculation
Core fucosylation	Fraction of structures containing core fucose in total IgG glycans	GP1+GP4+GP6+GP8+GP9+GP10+GP11+GP14+GP15+GP16+GP18+GP19+GP23+ GP24
Bisecting GlcNAc	Fraction of structures containing bisecting GlcNAc in total IgG glycans	GP3+GP6+GP10+GP11+GP13+GP15+GP19+GP22+GP24
Agalactosylation	Fraction of agalactosylated structures in total IgG glycans	GP1+GP2+GP3+GP4+GP5+GP6
Monogalactosylation	Fraction of structures containing one galactose in total IgG glycans	GP7+GP8+GP9+GP10+GP11+GP16
Digalactosylation	Fraction of structures containing two galactoses in total IgG glycans	GP12+GP13+GP14+GP15+GP17+GP18+GP19+GP20+GP21+GP22+GP23+GP24
Sialylation	Fraction of structures containing sialic acid in total IgG glycans	GP16+GP17+GP18+GP19+GP20+GP21+GP22+GP23+GP24

[1]Glycan structures are drawn in GlycoWorkbench version 2. blue square = N-acetylglucosamine, red triangle = fucose, green circle = mannose, yellow circle = galactose, purple diamond = N-acetylneuraminic acid.
[2]Total glycans = sum of all 24 glycan peaks.

Supplementary Table 4. Derived glycan traits in five populations used for HILIC-UPLC analysis of IgG glycosylation.

	Cohort	Min.	1st Qu.	Median	Mean	3rd Qu.	Max.
Agalactosylation	China	9,232	16,45	19,96	20,46	23,5	48,04
	Croatia	8,679	21,88	27,06	27,33	32,53	55,42
	Estonia	10,62	31,26	36,24	36,31	41,14	72,63
	Scotland	8,727	18,93	24,03	24,67	29,72	60,62
	England	7,458	19,21	24,6	25,29	30,52	63,45
Monogalactosylation	China	27,24	33,38	35,04	34,82	36,44	42,06
	Croatia	20,18	29,07	30,76	30,58	32,4	39,36
	Estonia	17,39	32,57	34,29	33,99	35,78	45,23
	Scotland	23,51	34,01	35,62	35,43	37,12	44,54
	England	22,7	34,68	36,21	36,02	37,55	49,37
Digalactosylation	China	7,297	19,07	21,86	21,76	24,6	33,82
	Croatia	4,603	11,33	13,75	14,26	16,72	29,7
	Estonia	3,129	10,32	12,43	12,69	14,8	26,02
	Scotland	5,246	14,09	17,12	17,52	20,65	35,39
	England	4,536	14,49	17,9	18,12	21,63	35,39
Bisecting GlcNAc	China	8,992	14,17	15,58	15,79	17,18	26,98
	Croatia	11,1	16,82	18,4	18,49	19,99	29,32
	Estonia	5,771	16,56	18,17	18,32	19,87	29,48
	Scotland	8,377	14,88	16,45	16,71	18,46	36,53
	England	6,958	15,24	16,96	17,17	18,9	32,23
Sialylation	China	12,3	20,62	22,92	22,95	25,37	33,51
	Croatia	13,32	24,26	27,33	27,83	30,93	51,82
	Estonia	4,63	14,97	16,74	17	18,82	39,29
	Scotland	10,3	19,62	22,16	22,37	25,05	36,94
	England	3,008	17,87	20,35	20,58	23,23	49,82
Core fucosylation	China	88,18	94,62	95,44	95,26	96,14	98,08
	Croatia	68,12	87,45	89,86	89,12	91,72	96,01
	Estonia	90,59	95,67	96,38	96,18	96,92	99,13
	Scotland	74	95,28	96,05	95,83	96,64	98,17
	England	81,95	94,74	95,59	95,36	96,28	98,14

In table are given: analysed population, glycan trait parameters (minimum, maximum, median, mean, 1st and 3rd quartile).

Supplementary Table 5. Linear mixed model in five populations with age and sex defined as fixed effects and country of residence as a random effect.

Derived glycan trait	Percent of glycan trait variability explained by country of residence (%)	Percent of glycan trait variability explained by age (%)	Percent of glycan trait variability explained by sex (%)	Country of residence *P* value
Agalactosylation	14.4	30.9	0.8	6.6×10^{-194}
Monogalactosylation	39.9	0.0	0.2	$< 6 \times 10^{-350}$
Digalactosylation	19.5	30.2	0.7	1.1×10^{-301}
Bisecting GlcNAc	6.3	20.1	0.1	6.7×10^{-104}
Sialylation	42.5	11.9	0.2	$< 6 \times 10^{-350}$
Core fucosylation	57.5	0.1	0.0	$< 6 \times 10^{-350}$

Displayed values represent percentage (%) glycan trait variability explained by age, sex and country of residence, with country of residence likelihood-ratio test *P* value.

Supplementary Table 6. Demographic characteristics of 27 populations used for IgG Fc glycosylation analysis.

Population	Country of residence	Cohort abbreviation	Min.	Q1	Median	Mean	Q3	Max.	F	M
Evenk minority	China	Evenk	10	22	29	33	41	85	29	58
General population of China (Han)	China	China	20	26	44	40	51	60	58	42
Kazak minority	China	ChiKaz	21	33	45	44	56	78	52	45
Kyrgyz minority	China	ChiKrz	22	55	65	62	70	92	56	41
Uyghur minority	China	ChiUyg	8	38	49	47	59	85	63	36
Yakut minority	Russia	Yakut	18	34	42	44	54	72	72	28
General population of Croatia	Croatia	Croatia	18	38	49	50	62	81	24	73
Roma minority	Croatia	Roma	18	28	37	38	42	79	48	51
General population of Germany	Germany	Germany	32	43	47	49	57	70	30	67
General population of Italy	Italy	Italy	24	34	46	47	58	86	48	41
General population of Kosovo	Kosovo	Kosovo	21	33	46	44	57	62	60	40
General population of Papua New Guinea	Papua New Guinea	NewGui	3	8	12	18	26	48	46	37
General population of Russia	Russia	Russia	9	17	20	23	26	43	62	38
Kazak minority	Russia	RuKaz	21	34	42	43	53	63	48	50
Tatar minority	Russia	RuTar	18	23	31	31	36	48	57	42
Yakut minority	Russia	RuYak	20	37	47	47	56	75	68	13
General population of Sweden	Sweden	Sweden	22	33	44	42	51	64	43	54
Uninfected HIV cohort participants from Thailand	Thailand	Thailand	18	28	32	33	36	49	49	50
General population of Trinidad and Tobago	Trinidad and Tobago	TriTob	22	40	53	50	59	77	81	15
General population of Turkey	Turkey	Turkey	16	48	59	57	67	79	38	62
General population of Uganda	Uganda	Uganda	17	29	33	33	37	47	54	44
General population of England (SABRE cohort)	England	SabEng	59	63	67	68	72	77	26	72
General population of England (TwinsUK cohort)	England	TwinsUK	21	33	46	46	57	70	55	42
British Indians (SABRE cohort)	England	SabInd	60	65	69	69	73	83	22	76
British Jamaicans (SABRE cohort)	England	SabJam	59	66	69	69	73	81	56	40
Population of Orkney Islands	Scotland	Orkney	21	33	46	45	56	70	52	26
Population of Shetland Islands	Scotland	Shetland	21	34	46	45	56	69	50	49

In table are given: country of residence of participants, abbreviation of analysed cohort, age parameters (minimum, maximum, median, mean, 1st and 3rd quartile) and sex parameters (number of female and male participants).
