# Supplementary material for: Global variability of the human IgG glycome
Source: Aging (Albany NY). 2020 Aug 12;12(15):15222–59. doi: 10.18632/aging.103884 (PMC7467356; doi:10.18632/aging.103884)
Supplement: Supplementary Table 7 [file aging-12-103884-s002..docx]

**Supplementary Table 7. Derived glycan traits in 27 populations used for IgG1 Fc glycopeptide analysis.** In table are given: abbreviation of analysed population, glycan trait parameters (minimum, maximum, median, mean, 1^st^ and 3^rd^ quartile). Population abbreviations are defined in Figure 2 and Supplementary Table 6.

|  | IgG1 Agalactosylation | | | | | | IgG1 Monogalactosylation | | | | | | IgG1 Digalactosylation | | | | | | IgG1 Sialylation | | | | | | IgG1 Bisecting | | | | | |
| --- | --- | --- | --- | --- | --- | --- | --- | --- | --- | --- | --- | --- | --- | --- | --- | --- | --- | --- | --- | --- | --- | --- | --- | --- | --- | --- | --- | --- | --- | --- |
| Pop | Min. | 1st Qu. | Median | Mean | 3rd Qu. | Max. | Min. | 1st Qu. | Median | Mean | 3rd Qu. | Max. | Min. | 1st Qu. | Median | Mean | 3rd Qu. | Max. | Min. | 1st Qu. | Median | Mean | 3rd Qu. | Max. | Min. | 1st Qu. | Median | Mean | 3rd Qu. | Max. |
| Evenk | 16,1 | 32,8 | 39,4 | 38,5 | 43,7 | 58,0 | 24,5 | 33,7 | 36,1 | 35,7 | 37,8 | 44,5 | 6,1 | 10,5 | 12,7 | 13,6 | 16,2 | 28,1 | 4,5 | 9,6 | 11,6 | 12,2 | 14,0 | 28,5 | 7,7 | 12,6 | 13,9 | 14,5 | 16,1 | 27,1 |
| Yakut | 17,0 | 26,9 | 34,0 | 35,4 | 41,7 | 57,2 | 27,2 | 35,5 | 37,8 | 37,4 | 39,3 | 45,0 | 6,0 | 12,2 | 15,1 | 15,6 | 18,3 | 27,3 | 4,8 | 8,7 | 12,4 | 11,6 | 14,1 | 20,9 | 8,6 | 11,6 | 13,6 | 14,0 | 16,4 | 21,0 |
| RuYak | 22,1 | 28,8 | 32,7 | 33,8 | 39,1 | 49,2 | 30,5 | 36,4 | 38,0 | 38,1 | 40,0 | 43,6 | 7,1 | 12,4 | 15,1 | 15,3 | 18,0 | 24,0 | 6,8 | 10,4 | 12,7 | 12,9 | 14,7 | 19,5 | 9,8 | 13,5 | 14,7 | 15,0 | 16,1 | 20,7 |
| ChiKrz | 22,0 | 39,1 | 43,3 | 43,3 | 47,9 | 64,4 | 21,2 | 33,7 | 36,0 | 35,2 | 37,4 | 42,7 | 4,0 | 9,7 | 11,4 | 11,9 | 13,8 | 23,8 | 4,7 | 7,7 | 9,2 | 9,6 | 11,0 | 23,9 | 8,7 | 13,7 | 15,6 | 15,7 | 17,3 | 24,3 |
| China | 15,8 | 24,7 | 28,5 | 29,4 | 33,7 | 46,5 | 31,1 | 37,1 | 39,2 | 39,2 | 40,9 | 47,7 | 10,0 | 16,1 | 19,0 | 18,7 | 21,3 | 29,5 | 4,6 | 10,1 | 12,6 | 12,7 | 14,8 | 20,7 | 9,0 | 12,3 | 13,8 | 14,0 | 15,7 | 22,5 |
| Thailand | 20,3 | 26,8 | 31,1 | 31,5 | 34,9 | 58,6 | 26,3 | 36,3 | 37,7 | 37,6 | 39,5 | 43,6 | 7,3 | 14,8 | 17,4 | 17,4 | 19,5 | 26,9 | 7,2 | 10,9 | 13,4 | 13,6 | 15,5 | 23,8 | 8,1 | 11,8 | 13,5 | 13,5 | 14,8 | 19,2 |
| ChiKaz | 16,8 | 29,9 | 35,3 | 35,8 | 41,1 | 60,3 | 27,0 | 36,1 | 38,0 | 37,9 | 40,4 | 45,4 | 5,4 | 13,0 | 15,8 | 15,9 | 19,1 | 26,3 | 3,1 | 8,5 | 10,5 | 10,4 | 12,0 | 19,1 | 8,5 | 13,3 | 14,3 | 14,4 | 15,6 | 21,6 |
| ChiUyg | 14,5 | 30,6 | 37,8 | 37,1 | 43,6 | 58,7 | 23,3 | 36,1 | 38,0 | 37,7 | 39,8 | 47,1 | 4,6 | 11,4 | 14,3 | 15,0 | 17,9 | 31,8 | 3,2 | 7,9 | 9,4 | 10,2 | 12,4 | 19,3 | 7,6 | 12,3 | 14,2 | 14,1 | 15,7 | 20,6 |
| RuKaz | 15,5 | 27,9 | 31,7 | 32,7 | 37,4 | 61,4 | 29,7 | 35,8 | 37,8 | 37,8 | 39,9 | 45,7 | 4,4 | 13,4 | 16,8 | 16,4 | 19,5 | 29,0 | 4,6 | 10,6 | 12,6 | 13,0 | 14,9 | 31,6 | 8,4 | 12,8 | 14,4 | 14,6 | 16,3 | 22,2 |
| SabJam | 15,5 | 26,8 | 30,3 | 30,6 | 34,6 | 51,2 | 30,6 | 38,5 | 40,6 | 40,6 | 42,5 | 45,7 | 6,7 | 13,4 | 15,6 | 15,7 | 17,6 | 25,5 | 6,9 | 10,6 | 12,5 | 13,0 | 15,0 | 24,3 | 8,0 | 12,6 | 14,8 | 14,7 | 16,2 | 22,3 |
| Germany | 16,1 | 27,6 | 31,5 | 33,0 | 39,2 | 53,0 | 30,9 | 39,1 | 41,8 | 41,1 | 42,8 | 47,5 | 7,2 | 12,3 | 14,7 | 15,2 | 17,6 | 28,4 | 4,3 | 8,6 | 10,5 | 10,7 | 12,9 | 17,7 | 9,8 | 14,4 | 16,3 | 16,6 | 19,2 | 23,5 |
| SabEng | 21,6 | 32,8 | 36,6 | 37,9 | 42,8 | 66,1 | 26,3 | 37,9 | 40,7 | 40,4 | 42,6 | 51,2 | 3,9 | 10,6 | 12,9 | 12,5 | 14,5 | 18,9 | 3,7 | 7,7 | 8,9 | 9,2 | 10,6 | 19,4 | 12,0 | 15,1 | 17,8 | 18,1 | 20,5 | 29,4 |
| TwinsUK | 14,2 | 23,8 | 28,3 | 29,2 | 32,8 | 50,7 | 33,3 | 39,6 | 42,0 | 41,4 | 43,3 | 48,7 | 8,1 | 13,4 | 17,3 | 16,9 | 19,5 | 27,3 | 4,1 | 10,0 | 12,4 | 12,5 | 14,9 | 24,3 | 10,3 | 13,5 | 16,1 | 16,0 | 17,7 | 24,7 |
| Sweden | 16,1 | 26,0 | 29,7 | 30,4 | 34,8 | 48,8 | 33,2 | 38,6 | 40,8 | 40,3 | 42,4 | 46,3 | 8,8 | 12,9 | 14,9 | 15,6 | 18,2 | 26,2 | 5,6 | 11,4 | 13,1 | 13,7 | 15,9 | 26,9 | 9,2 | 13,2 | 14,4 | 14,7 | 16,1 | 29,6 |
| Orkney | 13,8 | 25,2 | 31,0 | 30,7 | 35,8 | 48,1 | 32,7 | 38,6 | 40,5 | 40,2 | 42,3 | 44,3 | 8,2 | 12,1 | 15,0 | 15,4 | 18,0 | 26,5 | 4,8 | 11,6 | 13,0 | 13,7 | 14,9 | 23,1 | 9,0 | 13,3 | 15,1 | 15,1 | 17,0 | 24,5 |
| Croatia | 19,2 | 29,3 | 34,9 | 34,5 | 39,1 | 58,8 | 27,1 | 37,1 | 39,0 | 38,8 | 41,1 | 44,7 | 6,4 | 11,7 | 13,8 | 13,9 | 15,8 | 23,2 | 5,2 | 10,8 | 11,9 | 12,8 | 14,4 | 29,8 | 12,1 | 15,4 | 17,3 | 17,7 | 19,8 | 28,4 |
| Italy | 12,7 | 24,7 | 31,0 | 32,6 | 38,0 | 70,1 | 20,7 | 39,2 | 41,9 | 40,8 | 44,3 | 47,8 | 3,6 | 12,7 | 16,7 | 16,7 | 20,4 | 28,3 | 3,7 | 7,7 | 9,7 | 9,9 | 11,4 | 20,0 | 9,3 | 13,4 | 15,5 | 16,0 | 18,2 | 27,8 |
| Kosovo | 18,6 | 29,5 | 35,6 | 35,6 | 40,6 | 60,6 | 25,2 | 35,1 | 37,7 | 37,7 | 40,2 | 46,3 | 6,2 | 11,0 | 13,6 | 14,2 | 17,1 | 24,5 | 5,0 | 9,9 | 12,5 | 12,5 | 14,1 | 22,4 | 10,4 | 14,6 | 16,6 | 16,9 | 18,9 | 30,0 |
| Russia | 12,0 | 24,3 | 29,4 | 29,4 | 34,8 | 57,7 | 28,4 | 37,0 | 39,2 | 38,8 | 40,6 | 46,9 | 6,2 | 14,0 | 16,9 | 17,7 | 20,9 | 34,4 | 5,1 | 11,9 | 13,9 | 14,1 | 16,5 | 26,7 | 8,3 | 14,0 | 15,6 | 15,4 | 17,2 | 25,0 |
| RuTar | 15,4 | 24,4 | 28,8 | 29,0 | 32,5 | 46,8 | 30,9 | 37,8 | 39,7 | 39,6 | 41,7 | 45,1 | 10,1 | 15,0 | 17,2 | 17,4 | 20,0 | 29,8 | 7,2 | 12,1 | 13,9 | 14,0 | 16,0 | 22,5 | 9,5 | 12,9 | 14,5 | 14,5 | 15,8 | 20,3 |
| Turkey | 21,1 | 35,2 | 41,8 | 41,2 | 46,0 | 74,9 | 16,1 | 34,0 | 36,7 | 36,1 | 39,0 | 43,8 | 2,2 | 9,2 | 11,7 | 11,7 | 13,8 | 24,0 | 4,7 | 9,2 | 10,6 | 11,0 | 13,1 | 20,0 | 11,7 | 16,7 | 18,5 | 18,9 | 20,9 | 27,7 |
| Roma | 16,4 | 31,7 | 36,9 | 38,4 | 43,6 | 60,0 | 26,5 | 34,1 | 37,2 | 36,5 | 39,1 | 43,4 | 5,5 | 10,9 | 13,4 | 13,2 | 15,8 | 20,0 | 3,7 | 9,1 | 11,3 | 11,9 | 13,5 | 31,7 | 9,4 | 15,8 | 17,7 | 17,9 | 20,1 | 30,0 |
| SabInd | 25,9 | 36,2 | 40,4 | 41,0 | 45,4 | 61,1 | 27,1 | 36,0 | 38,8 | 38,1 | 41,0 | 45,1 | 6,2 | 10,0 | 11,9 | 11,9 | 13,7 | 18,6 | 3,7 | 7,1 | 8,6 | 9,0 | 10,7 | 15,2 | 12,3 | 16,7 | 18,7 | 19,0 | 20,7 | 33,2 |
| Ugand | 17,5 | 29,8 | 37,3 | 37,3 | 43,9 | 68,1 | 18,6 | 30,2 | 32,7 | 32,5 | 35,7 | 39,6 | 4,5 | 9,9 | 12,4 | 12,7 | 15,2 | 22,6 | 5,3 | 13,4 | 16,2 | 17,5 | 19,6 | 52,4 | 6,7 | 11,6 | 12,8 | 12,6 | 13,8 | 20,3 |
| Shetland | 19,8 | 33,0 | 37,8 | 39,0 | 43,8 | 68,1 | 22,1 | 35,7 | 39,0 | 38,1 | 41,3 | 48,4 | 3,5 | 9,8 | 12,0 | 12,0 | 13,9 | 19,7 | 3,9 | 8,5 | 10,3 | 10,9 | 12,5 | 29,3 | 8,9 | 12,9 | 15,5 | 15,5 | 17,8 | 25,0 |
| TriTob | 17,2 | 26,9 | 33,1 | 33,0 | 37,1 | 58,1 | 27,7 | 36,7 | 39,1 | 38,4 | 40,4 | 45,0 | 4,7 | 11,7 | 14,8 | 15,1 | 17,8 | 26,0 | 6,5 | 11,3 | 13,4 | 13,5 | 15,7 | 22,8 | 7,1 | 11,5 | 14,1 | 14,1 | 16,5 | 24,7 |
| NewGui | 26,6 | 38,9 | 44,9 | 46,0 | 51,9 | 63,8 | 17,4 | 27,2 | 29,0 | 28,5 | 30,8 | 36,1 | 3,8 | 8,8 | 10,3 | 10,8 | 12,5 | 22,0 | 3,9 | 11,9 | 14,1 | 14,8 | 17,0 | 24,7 | 6,2 | 9,4 | 10,3 | 10,3 | 11,1 | 16,4 |
