# Supplementary material for: Global variability of the human IgG glycome
Source: Aging (Albany NY). 2020 Aug 12;12(15):15222–59. doi: 10.18632/aging.103884 (PMC7467356; doi:10.18632/aging.103884)
Supplement: Supplementary Table 8 [file aging-12-103884-s004..docx]

**Supplementary Table 8. Derived glycan traits in 27 populations used for IgG2 Fc glycopeptide analysis.** In table are given: abbreviation of analysed population, glycan trait parameters (minimum, maximum, median, mean, 1^st^ and 3^rd^ quartile). Population abbreviations are defined in Figure 2 and Supplementary Table 6.

|  | IgG2 Agalactosylation | | | | | | IgG2 Monogalactosylation | | | | | | IgG2 Digalactosylation | | | | | | IgG2 Sialylation | | | | | | IgG4 Bisecting | | | | | |
| --- | --- | --- | --- | --- | --- | --- | --- | --- | --- | --- | --- | --- | --- | --- | --- | --- | --- | --- | --- | --- | --- | --- | --- | --- | --- | --- | --- | --- | --- | --- |
| Pop | Min. | 1st Qu. | Median | Mean | 3rd Qu. | Max. | Min. | 1st Qu. | Median | Mean | 3rd Qu. | Max. | Min. | 1st Qu. | Median | Mean | 3rd Qu. | Max. | Min. | 1st Qu. | Median | Mean | 3rd Qu. | Max. | Min. | 1st Qu. | Median | Mean | 3rd Qu. | Max. |
| Evenk | 24,1 | 38,3 | 43,8 | 44,6 | 50,3 | 64,2 | 24,2 | 30,0 | 32,4 | 32,3 | 34,1 | 39,2 | 5,8 | 9,6 | 12,7 | 12,8 | 15,1 | 24,2 | 3,8 | 7,6 | 9,9 | 10,3 | 11,8 | 22,8 | 6,0 | 9,8 | 10,9 | 11,1 | 12,5 | 18,1 |
| Yakut | 24,8 | 33,6 | 41,3 | 42,4 | 49,3 | 69,6 | 23,5 | 30,1 | 33,4 | 32,7 | 34,8 | 40,0 | 3,8 | 11,2 | 14,3 | 14,3 | 18,0 | 24,4 | 3,1 | 7,7 | 11,1 | 10,7 | 13,4 | 17,1 | 6,6 | 9,7 | 11,1 | 11,2 | 12,8 | 16,1 |
| RuYak | 26,8 | 35,4 | 39,1 | 41,5 | 48,6 | 63,4 | 24,3 | 31,2 | 33,1 | 33,2 | 35,7 | 39,6 | 5,5 | 11,8 | 14,5 | 14,2 | 16,4 | 24,1 | 3,0 | 8,9 | 11,0 | 11,1 | 13,1 | 21,7 | 7,7 | 10,4 | 11,3 | 11,6 | 12,8 | 17,1 |
| ChiKrz | 27,4 | 44,9 | 51,4 | 49,9 | 55,0 | 71,5 | 20,2 | 27,9 | 29,7 | 29,5 | 32,1 | 36,5 | 3,0 | 8,9 | 10,4 | 10,9 | 12,7 | 23,4 | 3,8 | 7,6 | 9,1 | 9,7 | 11,5 | 17,7 | 7,2 | 10,5 | 11,4 | 11,7 | 13,1 | 16,8 |
| China | 17,7 | 32,5 | 37,3 | 37,3 | 43,6 | 56,2 | 28,4 | 32,9 | 34,8 | 34,9 | 36,5 | 42,9 | 6,3 | 12,9 | 15,9 | 16,1 | 18,4 | 25,6 | 5,6 | 9,4 | 11,4 | 11,7 | 13,3 | 24,8 | 6,7 | 9,5 | 10,8 | 10,9 | 12,1 | 17,3 |
| Thailand | 22,6 | 30,5 | 34,0 | 34,5 | 37,8 | 52,4 | 26,5 | 32,7 | 34,2 | 34,0 | 35,3 | 41,2 | 8,6 | 14,9 | 17,0 | 17,1 | 19,0 | 26,2 | 8,1 | 12,4 | 14,4 | 14,5 | 16,2 | 21,6 | 6,8 | 9,1 | 10,4 | 10,6 | 12,0 | 14,7 |
| ChiKaz | 21,4 | 34,8 | 40,6 | 40,7 | 47,3 | 62,8 | 23,5 | 30,9 | 33,0 | 32,6 | 34,6 | 41,1 | 5,9 | 11,7 | 14,6 | 14,8 | 17,7 | 26,3 | 5,1 | 9,8 | 11,7 | 11,9 | 13,5 | 19,8 | 6,2 | 9,8 | 11,1 | 11,1 | 12,2 | 16,4 |
| ChiUyg | 20,8 | 37,3 | 44,6 | 43,5 | 49,5 | 72,9 | 16,1 | 29,7 | 31,5 | 31,5 | 33,6 | 40,2 | 4,2 | 10,0 | 12,4 | 13,5 | 15,9 | 28,2 | 3,9 | 8,5 | 10,9 | 11,6 | 14,1 | 21,6 | 4,9 | 9,5 | 10,8 | 10,8 | 11,9 | 16,7 |
| RuKaz | 24,0 | 34,4 | 37,9 | 39,7 | 44,8 | 59,2 | 27,1 | 31,0 | 33,2 | 33,2 | 34,9 | 40,4 | 7,2 | 12,0 | 15,0 | 15,0 | 17,4 | 26,4 | 5,6 | 10,1 | 11,7 | 12,1 | 14,0 | 23,1 | 5,7 | 9,6 | 11,1 | 11,3 | 12,5 | 18,7 |
| SabJam | 21,8 | 35,5 | 40,1 | 40,0 | 45,2 | 60,4 | 23,6 | 33,0 | 34,4 | 34,3 | 36,1 | 40,6 | 7,8 | 11,3 | 13,3 | 13,5 | 15,8 | 21,0 | 5,7 | 9,9 | 12,0 | 12,2 | 14,2 | 22,8 | 4,5 | 9,6 | 11,0 | 11,1 | 12,3 | 18,6 |
| Germany | 24,2 | 35,3 | 41,1 | 41,5 | 47,3 | 68,0 | 20,0 | 32,1 | 34,6 | 34,1 | 36,8 | 46,1 | 5,9 | 10,6 | 12,8 | 13,0 | 15,6 | 22,7 | 6,1 | 9,2 | 11,5 | 11,4 | 13,0 | 24,0 | 7,2 | 10,1 | 12,2 | 12,0 | 13,6 | 16,5 |
| SabEng | 32,3 | 42,0 | 46,1 | 46,9 | 51,4 | 72,3 | 19,8 | 30,4 | 32,9 | 32,7 | 35,7 | 39,8 | 3,4 | 8,9 | 10,5 | 10,5 | 12,1 | 16,6 | 4,4 | 8,3 | 9,9 | 9,8 | 11,1 | 17,0 | 7,7 | 12,1 | 13,8 | 14,1 | 16,0 | 25,9 |
| TwinsUK | 21,8 | 30,7 | 36,3 | 37,3 | 43,5 | 58,7 | 22,8 | 32,3 | 35,0 | 34,7 | 36,8 | 42,7 | 7,0 | 11,6 | 14,5 | 14,7 | 17,4 | 24,8 | 4,4 | 9,7 | 13,6 | 13,4 | 16,1 | 21,7 | 6,7 | 10,1 | 11,4 | 11,8 | 13,3 | 18,9 |
| Sweden | 20,2 | 34,7 | 41,1 | 40,5 | 45,6 | 65,9 | 23,2 | 32,4 | 34,0 | 34,0 | 36,2 | 40,1 | 5,2 | 10,5 | 12,8 | 13,2 | 15,7 | 23,2 | 5,3 | 9,9 | 11,8 | 12,3 | 14,5 | 22,0 | 6,6 | 9,9 | 11,0 | 11,1 | 12,2 | 16,3 |
| Orkney | 22,3 | 35,2 | 42,2 | 41,9 | 48,7 | 62,2 | 23,8 | 31,5 | 33,2 | 33,0 | 35,3 | 39,0 | 5,7 | 9,8 | 12,6 | 13,0 | 15,5 | 24,4 | 4,5 | 10,0 | 12,3 | 12,1 | 14,0 | 21,1 | 8,2 | 9,9 | 11,2 | 11,4 | 12,5 | 17,4 |
| Croatia | 25,8 | 39,3 | 43,7 | 44,0 | 49,4 | 67,2 | 21,6 | 30,5 | 33,1 | 32,5 | 34,8 | 40,3 | 4,1 | 10,0 | 11,7 | 12,0 | 14,0 | 22,4 | 4,6 | 9,5 | 11,2 | 11,5 | 12,7 | 21,8 | 9,3 | 10,5 | 12,3 | 12,7 | 14,6 | 20,3 |
| Italy | 17,8 | 30,9 | 37,8 | 38,4 | 44,0 | 67,5 | 21,2 | 32,7 | 36,4 | 35,3 | 38,5 | 42,8 | 5,4 | 12,2 | 14,9 | 15,2 | 18,8 | 25,9 | 4,5 | 7,8 | 11,2 | 11,1 | 13,1 | 21,2 | 7,5 | 10,7 | 12,2 | 12,5 | 13,5 | 18,9 |
| Kosovo | 24,5 | 36,0 | 42,2 | 43,4 | 49,7 | 65,9 | 20,8 | 29,1 | 31,9 | 31,3 | 33,7 | 39,5 | 4,7 | 9,8 | 12,3 | 12,8 | 15,2 | 22,1 | 5,7 | 10,1 | 12,2 | 12,5 | 14,6 | 25,6 | 7,2 | 11,1 | 12,2 | 12,7 | 13,9 | 24,0 |
| Russia | 18,0 | 31,1 | 36,9 | 36,7 | 42,1 | 61,4 | 26,2 | 31,2 | 33,2 | 33,0 | 35,2 | 40,3 | 5,3 | 13,2 | 15,6 | 15,9 | 18,3 | 31,4 | 5,5 | 11,9 | 13,8 | 14,3 | 16,7 | 26,1 | 5,5 | 10,7 | 11,9 | 11,8 | 13,0 | 17,0 |
| RuTar | 22,4 | 30,7 | 36,2 | 36,3 | 40,6 | 55,7 | 25,4 | 32,3 | 34,4 | 34,0 | 36,0 | 41,0 | 9,2 | 14,0 | 16,0 | 16,0 | 18,0 | 26,3 | 6,3 | 11,2 | 13,8 | 13,7 | 15,9 | 22,3 | 6,6 | 10,0 | 10,9 | 11,1 | 12,2 | 18,3 |
| Turkey | 22,5 | 42,1 | 47,9 | 47,7 | 53,1 | 79,6 | 13,8 | 28,7 | 31,1 | 30,7 | 33,1 | 38,6 | 2,2 | 8,5 | 10,3 | 10,9 | 13,1 | 24,8 | 4,4 | 8,7 | 10,4 | 10,8 | 12,1 | 23,7 | 8,3 | 12,0 | 13,5 | 13,8 | 15,2 | 20,0 |
| Roma | 29,4 | 38,2 | 42,6 | 44,6 | 50,1 | 67,3 | 21,6 | 29,0 | 31,8 | 31,2 | 33,9 | 38,6 | 4,4 | 9,8 | 12,4 | 12,4 | 14,8 | 20,8 | 4,0 | 8,9 | 12,3 | 11,8 | 14,2 | 19,4 | 5,5 | 11,3 | 13,3 | 13,2 | 14,7 | 22,0 |
| SabInd | 23,8 | 44,0 | 48,7 | 48,3 | 52,5 | 65,3 | 21,7 | 29,1 | 31,6 | 31,3 | 33,3 | 39,2 | 5,4 | 8,5 | 10,2 | 10,4 | 12,1 | 17,5 | 4,1 | 7,9 | 9,5 | 10,1 | 11,3 | 27,6 | 8,8 | 13,3 | 14,3 | 14,9 | 16,2 | 28,7 |
| Ugand | 22,1 | 33,9 | 41,2 | 41,3 | 48,5 | 59,7 | 22,6 | 28,8 | 31,2 | 30,8 | 32,9 | 38,2 | 6,7 | 10,5 | 12,8 | 13,2 | 16,1 | 21,8 | 6,5 | 11,7 | 14,0 | 14,7 | 16,6 | 29,9 | 6,6 | 8,7 | 9,5 | 10,1 | 11,3 | 16,8 |
| Shetland | 31,9 | 41,8 | 45,6 | 47,7 | 52,8 | 70,5 | 21,0 | 30,0 | 32,1 | 31,9 | 34,5 | 40,5 | 2,5 | 7,9 | 9,9 | 9,9 | 12,2 | 16,3 | 2,9 | 8,1 | 10,2 | 10,5 | 12,6 | 26,3 | 6,9 | 10,9 | 12,4 | 12,6 | 14,3 | 20,7 |
| TriTob | 23,1 | 34,3 | 42,4 | 41,8 | 47,4 | 67,5 | 24,3 | 31,6 | 33,8 | 33,5 | 35,9 | 40,1 | 3,8 | 10,1 | 12,5 | 13,2 | 16,1 | 23,3 | 3,4 | 9,1 | 11,3 | 11,6 | 13,8 | 22,7 | 5,9 | 8,5 | 10,4 | 10,5 | 12,0 | 17,7 |
| NewGui | 33,3 | 43,0 | 48,8 | 47,8 | 52,2 | 71,9 | 18,4 | 26,5 | 28,4 | 28,3 | 30,4 | 38,4 | 4,6 | 9,8 | 11,9 | 11,9 | 13,8 | 22,1 | 5,1 | 9,5 | 12,0 | 12,0 | 14,7 | 19,5 | 5,2 | 8,5 | 9,5 | 9,5 | 10,2 | 20,4 |
