# Supplementary material for: Global variability of the human IgG glycome
Source: Aging (Albany NY). 2020 Aug 12;12(15):15222–59. doi: 10.18632/aging.103884 (PMC7467356; doi:10.18632/aging.103884)
Supplement: Supplementary Table 10 [file aging-12-103884-s005..docx]

**Supplementary Table 10. Correlations of IgG Fc derived glycan traits with participant’s country of residence development indicators.** Pearson's correlation coefficient with accompanying P values are given (n=14) and P values adjusted for multiple testing. Abbreviations: MMR = maternal mortality ratio; SBA = skilled birth attendance; Nnmort = neonatal mortality; NTDs = neglected tropical diseases; NCDs = non-communicable diseases; FPneedmet = family planning need met, modern contraception; Adol = Adolescent birth rate; UHC = universal health coverage; Air poll mort = Air pollution mortality; WaSH = water, sanitation, and hygiene; IPV = intimate partner violence; HHairpoll = household air pollution; MDG = Millenium Development Goals; HDI = Human development Index; SDG = Sustainable Development Goals. Development indicators are described in Supplementary Table 11 and Supplementary Table 12.

| Glycan Trait | Development index/indicator | r_p_ | P value | adjusted P value |
| --- | --- | --- | --- | --- |
| IgG1_Monogalactosylation | MDG | 0,969434756 | 1,10E-08 | 7,44E-06 |
| IgG1_Monogalactosylation | Stunting | 0,967055014 | 1,72E-08 | 1,16E-05 |
| IgG1_Monogalactosylation | HDI | 0,965825352 | 2,14E-08 | 1,44E-05 |
| IgG1_Agalactosylation | UHCTracer | -0,95037426 | 1,94E-07 | 1,31E-04 |
| IgG1_Agalactosylation | OccRiskBurden | -0,950003444 | 2,02E-07 | 1,37E-04 |
| IgG1_Monogalactosylation | UHCTracer | 0,945363091 | 3,41E-07 | 2,30E-04 |
| IgG1_Agalactosylation | Stunting | -0,9420058 | 4,84E-07 | 3,27E-04 |
| IgG1_Monogalactosylation | SDG | 0,940338227 | 5,72E-07 | 3,86E-04 |
| IgG1_Monogalactosylation | EduIndx_2014 | 0,937368449 | 7,61E-07 | 5,14E-04 |
| IgG1_Monogalactosylation | OccRiskBurden | 0,93089952 | 1,35E-06 | 9,13E-04 |
| IgG1_Digalactosylation | SBA | 0,930467933 | 1,40E-06 | 9,47E-04 |
| IgG1_Digalactosylation | Stunting | 0,923405123 | 2,47E-06 | 1,67E-03 |
| IgG1_Monogalactosylation | LifeExp_F | 0,916985412 | 3,94E-06 | 2,66E-03 |
| IgG1_Monogalactosylation | AirPollMort | 0,914275509 | 4,75E-06 | 3,21E-03 |
| IgG1_Agalactosylation | HDI | -0,906023161 | 8,10E-06 | 5,47E-03 |
| IgG1_Monogalactosylation | HealthIndx_2014 | 0,901032539 | 1,09E-05 | 7,38E-03 |
| IgG1_Monogalactosylation | LifeExp | 0,901010147 | 1,09E-05 | 7,39E-03 |
| IgG1_Agalactosylation | MDG | -0,899285831 | 1,21E-05 | 8,16E-03 |
| IgG1_Monogalactosylation | Non.MDG | 0,898094676 | 1,29E-05 | 8,73E-03 |
| IgG1_Agalactosylation | EduIndx_2014 | -0,895317502 | 1,51E-05 | 1,02E-02 |
| IgG1_Monogalactosylation | HepatB | 0,895249048 | 1,52E-05 | 1,02E-02 |
| IgG1_Agalactosylation | Water_UN | -0,891382686 | 1,87E-05 | 1,26E-02 |
| IgG2_Monogalactosylation | Stunting | 0,890858888 | 1,92E-05 | 1,30E-02 |
| IgG1_Agalactosylation | SBA | -0,890488563 | 1,96E-05 | 1,32E-02 |
| IgG1_Monogalactosylation | MMR | 0,890356305 | 1,97E-05 | 1,33E-02 |
| IgG1_Monogalactosylation | GDP_2013 | 0,888528777 | 2,17E-05 | 1,46E-02 |
| IgG1_Monogalactosylation | Sanitation_UN | 0,884836577 | 2,62E-05 | 1,77E-02 |
| IgG1_Monogalactosylation | Hygiene | 0,881989588 | 3,01E-05 | 2,03E-02 |
| IgG1_Digalactosylation | Water_UN | 0,880105424 | 3,30E-05 | 2,22E-02 |
| IgG1_Monogalactosylation | SBA | 0,875839535 | 4,03E-05 | 2,72E-02 |
| IgG1_Monogalactosylation | WaSHMort | 0,87368469 | 4,44E-05 | 3,00E-02 |
| IgG4_Monogalactosylation | Water_UN | 0,872607839 | 4,66E-05 | 3,15E-02 |
| IgG1_Agalactosylation | AirPollMort | -0,871570709 | 4,88E-05 | 3,30E-02 |
| IgG1_Agalactosylation | SDG | -0,871295686 | 4,94E-05 | 3,34E-02 |
| IgG1_Monogalactosylation | AdolBirthRate | 0,870585663 | 5,10E-05 | 3,44E-02 |
| IgG1_Monogalactosylation | LifeExp_M | 0,868743447 | 5,53E-05 | 3,73E-02 |
| IgG1_Digalactosylation | Sanitation_UN | 0,867063308 | 5,95E-05 | 4,01E-02 |
| IgG1_Digalactosylation | OccRiskBurden | 0,866441892 | 6,11E-05 | 4,12E-02 |
| IgG1_Monogalactosylation | Under5Mort | 0,866368769 | 6,13E-05 | 4,14E-02 |
| IgG2_Monogalactosylation | HDI | 0,864097812 | 6,74E-05 | 4,55E-02 |
| IgG2_Monogalactosylation | OccRiskBurden | 0,864088251 | 6,75E-05 | 4,55E-02 |
| IgG2_Monogalactosylation | MDG | 0,863881556 | 6,80E-05 | 4,59E-02 |
| IgG2_Monogalactosylation | AirPollMort | 0,862569569 | 7,19E-05 | 4,85E-02 |
| IgG1_Agalactosylation | Sanitation_UN | -0,861935177 | 7,38E-05 | 4,98E-02 |
| IgG1_Digalactosylation | MDG | 0,861366104 | 7,55E-05 | 5,10E-02 |
| IgG1_Digalactosylation | HDI | 0,860780423 | 7,74E-05 | 5,22E-02 |
| IgG2_Monogalactosylation | UHCTracer | 0,853575289 | 1,03E-04 | 6,95E-02 |
| IgG1_Digalactosylation | UHCTracer | 0,848991406 | 1,23E-04 | 8,28E-02 |
| IgG4_Monogalactosylation | OccRiskBurden | 0,846935798 | 1,32E-04 | 8,94E-02 |
| IgG4_Monogalactosylation | UHCTracer | 0,845494245 | 1,40E-04 | 9,42E-02 |
| IgG2_Monogalactosylation | SDG | 0,844836201 | 1,43E-04 | 9,65E-02 |
| IgG2_Agalactosylation | Water_UN | -0,843662929 | 1,49E-04 | 1,01E-01 |
| IgG4_Monogalactosylation | Stunting | 0,842223049 | 1,57E-04 | 1,06E-01 |
| IgG2_Monogalactosylation | LifeExp_F | 0,84175077 | 1,60E-04 | 1,08E-01 |
| IgG1_Digalactosylation | LifeExp_F | 0,840123908 | 1,69E-04 | 1,14E-01 |
| IgG1_Agalactosylation | Non.MDG | -0,839575445 | 1,73E-04 | 1,16E-01 |
| IgG2_Monogalactosylation | GDP_2013 | 0,837973778 | 1,82E-04 | 1,23E-01 |
| IgG1_Agalactosylation | HepatB | -0,833457052 | 2,13E-04 | 1,44E-01 |
| IgG1_Digalactosylation | MMR | 0,829385853 | 2,44E-04 | 1,65E-01 |
| IgG4_Monogalactosylation | SBA | 0,827296068 | 2,61E-04 | 1,76E-01 |
| IgG1_Monogalactosylation | Water_UN | 0,826515112 | 2,68E-04 | 1,81E-01 |
| IgG2_Agalactosylation | Stunting | -0,826331354 | 2,69E-04 | 1,82E-01 |
| IgG1_Agalactosylation | LifeExp_F | -0,824494857 | 2,86E-04 | 1,93E-01 |
| IgG2_Agalactosylation | SBA | -0,823894008 | 2,91E-04 | 1,97E-01 |
| IgG2_Monogalactosylation | Hygiene | 0,823431265 | 2,96E-04 | 2,00E-01 |
| IgG1_Monogalactosylation | RoadInjuries | 0,823239508 | 2,97E-04 | 2,01E-01 |
| IgG1_Monogalactosylation | Nnmort | 0,822245754 | 3,07E-04 | 2,07E-01 |
| IgG1_Agalactosylation | GDP_2013 | -0,821517563 | 3,14E-04 | 2,12E-01 |
| IgG1_Monogalactosylation | NCDs | 0,821496789 | 3,14E-04 | 2,12E-01 |
| IgG1_Agalactosylation | MMR | -0,821489913 | 3,14E-04 | 2,12E-01 |
| IgG2_Monogalactosylation | LifeExp | 0,820466756 | 3,24E-04 | 2,19E-01 |
| IgG2_Monogalactosylation | HealthIndx_2014 | 0,820204125 | 3,27E-04 | 2,21E-01 |
| IgG2_Monogalactosylation | Non.MDG | 0,812966233 | 4,08E-04 | 2,75E-01 |
| IgG2_Monogalactosylation | SBA | 0,812231749 | 4,17E-04 | 2,81E-01 |
| IgG4_Agalactosylation | Water_UN | -0,809870949 | 4,47E-04 | 3,01E-01 |
| IgG2_Agalactosylation | OccRiskBurden | -0,809791983 | 4,48E-04 | 3,02E-01 |
| IgG2_Monogalactosylation | Sanitation_UN | 0,807061294 | 4,85E-04 | 3,27E-01 |
| IgG1_Digalactosylation | LifeExp | 0,806219491 | 4,96E-04 | 3,35E-01 |
| IgG1_Agalactosylation | Hygiene | -0,806160418 | 4,97E-04 | 3,36E-01 |
| IgG1_Digalactosylation | HealthIndx_2014 | 0,806030521 | 4,99E-04 | 3,37E-01 |
| IgG1_Digalactosylation | SDG | 0,804797471 | 5,17E-04 | 3,49E-01 |
| IgG1_Digalactosylation | EduIndx_2014 | 0,802333078 | 5,54E-04 | 3,74E-01 |
| IgG1_Agalactosylation | WaSHMort | -0,798814855 | 6,11E-04 | 4,12E-01 |
| IgG1_Agalactosylation | HealthIndx_2014 | -0,797763059 | 6,29E-04 | 4,25E-01 |
| IgG1_Agalactosylation | LifeExp | -0,797752927 | 6,29E-04 | 4,25E-01 |
| IgG2_Monogalactosylation | EduIndx_2014 | 0,79697559 | 6,43E-04 | 4,34E-01 |
| IgG1_Digalactosylation | WaSHMort | 0,796649312 | 6,48E-04 | 4,38E-01 |
| IgG2_Monogalactosylation | Under5Mort | 0,794179045 | 6,93E-04 | 4,68E-01 |
| IgG2_Digalactosylation | SBA | 0,793871394 | 6,99E-04 | 4,72E-01 |
| IgG1_Bisecting | Water_UN | 0,791100782 | 7,52E-04 | 5,08E-01 |
| IgG1_Bisecting | WaSHMort | 0,789357384 | 7,87E-04 | 5,32E-01 |
| IgG2_Monogalactosylation | LifeExp_M | 0,787301248 | 8,31E-04 | 5,61E-01 |
| IgG1_Monogalactosylation | HHAirPoll | 0,786664858 | 8,44E-04 | 5,70E-01 |
| IgG1_Digalactosylation | Under5Mort | 0,786047351 | 8,58E-04 | 5,79E-01 |
| IgG2_Monogalactosylation | MMR | 0,785717063 | 8,65E-04 | 5,84E-01 |
| IgG1_Digalactosylation | AirPollMort | 0,785520456 | 8,70E-04 | 5,87E-01 |
| IgG2_Agalactosylation | Sanitation_UN | -0,783580847 | 9,14E-04 | 6,17E-01 |
| IgG2_Monogalactosylation | HepatB | 0,780914085 | 9,77E-04 | 6,60E-01 |
| IgG1_Agalactosylation | Under5Mort | -0,779220205 | 1,02E-03 | 6,88E-01 |
| IgG2_Agalactosylation | UHCTracer | -0,77700753 | 1,08E-03 | 7,27E-01 |
| IgG4_Monogalactosylation | HDI | 0,776086511 | 1,10E-03 | 7,43E-01 |
| IgG2_Monogalactosylation | AdolBirthRate | 0,774879532 | 1,13E-03 | 7,66E-01 |
| IgG4_Monogalactosylation | MDG | 0,771267904 | 1,24E-03 | 8,35E-01 |
| IgG1_Bisecting | MMR | 0,770597406 | 1,26E-03 | 8,49E-01 |
| IgG1_Monogalactosylation | Wasting | 0,767918251 | 1,34E-03 | 9,04E-01 |
| IgG4_Monogalactosylation | Sanitation_UN | 0,767152449 | 1,36E-03 | 9,21E-01 |
| IgG2_Monogalactosylation | NCDs | 0,766742835 | 1,38E-03 | 9,29E-01 |
| IgG4_Monogalactosylation | EduIndx_2014 | 0,764681027 | 1,44E-03 | 9,75E-01 |
| IgG1_Sialylation | HDI | -0,512835432 | 6,08E-02 | 1,00E+00 |
| IgG1_Bisecting | HDI | 0,647646104 | 1,23E-02 | 1,00E+00 |
| IgG2_Agalactosylation | HDI | -0,756061358 | 1,76E-03 | 1,00E+00 |
| IgG2_Digalactosylation | HDI | 0,649999603 | 1,19E-02 | 1,00E+00 |
| IgG2_Sialylation | HDI | 0,121510885 | 6,79E-01 | 1,00E+00 |
| IgG2_Bisecting | HDI | 0,529085703 | 5,17E-02 | 1,00E+00 |
| IgG4_Agalactosylation | HDI | -0,395134154 | 1,62E-01 | 1,00E+00 |
| IgG4_Digalactosylation | HDI | 0,240297289 | 4,08E-01 | 1,00E+00 |
| IgG4_Sialylation | HDI | -0,052923302 | 8,57E-01 | 1,00E+00 |
| IgG4_Bisecting | HDI | 0,030439221 | 9,18E-01 | 1,00E+00 |
| IgG1_Sialylation | SDG | -0,468841347 | 9,08E-02 | 1,00E+00 |
| IgG1_Bisecting | SDG | 0,529506192 | 5,15E-02 | 1,00E+00 |
| IgG2_Agalactosylation | SDG | -0,704767262 | 4,88E-03 | 1,00E+00 |
| IgG2_Digalactosylation | SDG | 0,58127416 | 2,92E-02 | 1,00E+00 |
| IgG2_Sialylation | SDG | 0,073926851 | 8,02E-01 | 1,00E+00 |
| IgG2_Bisecting | SDG | 0,411231552 | 1,44E-01 | 1,00E+00 |
| IgG4_Agalactosylation | SDG | -0,316231061 | 2,71E-01 | 1,00E+00 |
| IgG4_Monogalactosylation | SDG | 0,725112788 | 3,34E-03 | 1,00E+00 |
| IgG4_Digalactosylation | SDG | 0,16147417 | 5,81E-01 | 1,00E+00 |
| IgG4_Sialylation | SDG | -0,112824422 | 7,01E-01 | 1,00E+00 |
| IgG4_Bisecting | SDG | 0,009620853 | 9,74E-01 | 1,00E+00 |
| IgG1_Agalactosylation | Disaster | -0,68565858 | 6,79E-03 | 1,00E+00 |
| IgG1_Monogalactosylation | Disaster | 0,6608941 | 1,01E-02 | 1,00E+00 |
| IgG1_Digalactosylation | Disaster | 0,479713009 | 8,26E-02 | 1,00E+00 |
| IgG1_Sialylation | Disaster | 0,111545691 | 7,04E-01 | 1,00E+00 |
| IgG1_Bisecting | Disaster | 0,209030447 | 4,73E-01 | 1,00E+00 |
| IgG2_Agalactosylation | Disaster | -0,411580883 | 1,44E-01 | 1,00E+00 |
| IgG2_Monogalactosylation | Disaster | 0,587857022 | 2,70E-02 | 1,00E+00 |
| IgG2_Digalactosylation | Disaster | 0,188855976 | 5,18E-01 | 1,00E+00 |
| IgG2_Sialylation | Disaster | 0,157024067 | 5,92E-01 | 1,00E+00 |
| IgG2_Bisecting | Disaster | -0,000407641 | 9,99E-01 | 1,00E+00 |
| IgG4_Agalactosylation | Disaster | -0,285038409 | 3,23E-01 | 1,00E+00 |
| IgG4_Monogalactosylation | Disaster | 0,582205084 | 2,89E-02 | 1,00E+00 |
| IgG4_Digalactosylation | Disaster | 0,087805905 | 7,65E-01 | 1,00E+00 |
| IgG4_Sialylation | Disaster | 0,078446819 | 7,90E-01 | 1,00E+00 |
| IgG4_Bisecting | Disaster | -0,013813054 | 9,63E-01 | 1,00E+00 |
| IgG1_Sialylation | Stunting | -0,518666635 | 5,74E-02 | 1,00E+00 |
| IgG1_Bisecting | Stunting | 0,640431247 | 1,36E-02 | 1,00E+00 |
| IgG2_Digalactosylation | Stunting | 0,737032466 | 2,63E-03 | 1,00E+00 |
| IgG2_Sialylation | Stunting | 0,191858217 | 5,11E-01 | 1,00E+00 |
| IgG2_Bisecting | Stunting | 0,485180471 | 7,87E-02 | 1,00E+00 |
| IgG4_Agalactosylation | Stunting | -0,524210355 | 5,43E-02 | 1,00E+00 |
| IgG4_Digalactosylation | Stunting | 0,390887956 | 1,67E-01 | 1,00E+00 |
| IgG4_Sialylation | Stunting | 0,039326919 | 8,94E-01 | 1,00E+00 |
| IgG4_Bisecting | Stunting | -0,077366962 | 7,93E-01 | 1,00E+00 |
| IgG1_Agalactosylation | Wasting | -0,622517725 | 1,74E-02 | 1,00E+00 |
| IgG1_Digalactosylation | Wasting | 0,558535171 | 3,79E-02 | 1,00E+00 |
| IgG1_Sialylation | Wasting | -0,54822426 | 4,24E-02 | 1,00E+00 |
| IgG1_Bisecting | Wasting | 0,425687396 | 1,29E-01 | 1,00E+00 |
| IgG2_Agalactosylation | Wasting | -0,486896407 | 7,74E-02 | 1,00E+00 |
| IgG2_Monogalactosylation | Wasting | 0,680690058 | 7,37E-03 | 1,00E+00 |
| IgG2_Digalactosylation | Wasting | 0,376317069 | 1,85E-01 | 1,00E+00 |
| IgG2_Sialylation | Wasting | -0,160863862 | 5,83E-01 | 1,00E+00 |
| IgG2_Bisecting | Wasting | 0,427929189 | 1,27E-01 | 1,00E+00 |
| IgG4_Agalactosylation | Wasting | 0,004851465 | 9,87E-01 | 1,00E+00 |
| IgG4_Monogalactosylation | Wasting | 0,475830488 | 8,55E-02 | 1,00E+00 |
| IgG4_Digalactosylation | Wasting | -0,15516085 | 5,96E-01 | 1,00E+00 |
| IgG4_Sialylation | Wasting | -0,375822845 | 1,85E-01 | 1,00E+00 |
| IgG4_Bisecting | Wasting | 0,259264679 | 3,71E-01 | 1,00E+00 |
| IgG1_Agalactosylation | Overweight | 0,440063315 | 1,15E-01 | 1,00E+00 |
| IgG1_Monogalactosylation | Overweight | -0,597639054 | 2,40E-02 | 1,00E+00 |
| IgG1_Digalactosylation | Overweight | -0,474084685 | 8,68E-02 | 1,00E+00 |
| IgG1_Sialylation | Overweight | 0,651490247 | 1,16E-02 | 1,00E+00 |
| IgG1_Bisecting | Overweight | -0,540272828 | 4,61E-02 | 1,00E+00 |
| IgG2_Agalactosylation | Overweight | 0,38036389 | 1,80E-01 | 1,00E+00 |
| IgG2_Monogalactosylation | Overweight | -0,543930227 | 4,44E-02 | 1,00E+00 |
| IgG2_Digalactosylation | Overweight | -0,383445469 | 1,76E-01 | 1,00E+00 |
| IgG2_Sialylation | Overweight | 0,296425717 | 3,03E-01 | 1,00E+00 |
| IgG2_Bisecting | Overweight | -0,586556576 | 2,75E-02 | 1,00E+00 |
| IgG4_Agalactosylation | Overweight | 0,007801878 | 9,79E-01 | 1,00E+00 |
| IgG4_Monogalactosylation | Overweight | -0,38011317 | 1,80E-01 | 1,00E+00 |
| IgG4_Digalactosylation | Overweight | 0,081318441 | 7,82E-01 | 1,00E+00 |
| IgG4_Sialylation | Overweight | 0,301473685 | 2,95E-01 | 1,00E+00 |
| IgG4_Bisecting | Overweight | -0,414746144 | 1,40E-01 | 1,00E+00 |
| IgG1_Sialylation | MMR | -0,626475779 | 1,65E-02 | 1,00E+00 |
| IgG2_Agalactosylation | MMR | -0,743594726 | 2,30E-03 | 1,00E+00 |
| IgG2_Digalactosylation | MMR | 0,676905841 | 7,84E-03 | 1,00E+00 |
| IgG2_Sialylation | MMR | 0,176345168 | 5,46E-01 | 1,00E+00 |
| IgG2_Bisecting | MMR | 0,698558966 | 5,45E-03 | 1,00E+00 |
| IgG4_Agalactosylation | MMR | -0,426005718 | 1,29E-01 | 1,00E+00 |
| IgG4_Monogalactosylation | MMR | 0,707747908 | 4,63E-03 | 1,00E+00 |
| IgG4_Digalactosylation | MMR | 0,315794855 | 2,71E-01 | 1,00E+00 |
| IgG4_Sialylation | MMR | 0,003984067 | 9,89E-01 | 1,00E+00 |
| IgG4_Bisecting | MMR | 0,142564154 | 6,27E-01 | 1,00E+00 |
| IgG1_Sialylation | SBA | -0,506135755 | 6,48E-02 | 1,00E+00 |
| IgG1_Bisecting | SBA | 0,676067183 | 7,94E-03 | 1,00E+00 |
| IgG2_Sialylation | SBA | 0,260388753 | 3,69E-01 | 1,00E+00 |
| IgG2_Bisecting | SBA | 0,507473706 | 6,40E-02 | 1,00E+00 |
| IgG4_Agalactosylation | SBA | -0,627201116 | 1,64E-02 | 1,00E+00 |
| IgG4_Digalactosylation | SBA | 0,540164578 | 4,61E-02 | 1,00E+00 |
| IgG4_Sialylation | SBA | 0,171052148 | 5,59E-01 | 1,00E+00 |
| IgG4_Bisecting | SBA | -0,188359717 | 5,19E-01 | 1,00E+00 |
| IgG1_Sialylation | Under5Mort | -0,586299164 | 2,76E-02 | 1,00E+00 |
| IgG1_Bisecting | Under5Mort | 0,589730559 | 2,64E-02 | 1,00E+00 |
| IgG2_Agalactosylation | Under5Mort | -0,712602803 | 4,23E-03 | 1,00E+00 |
| IgG2_Digalactosylation | Under5Mort | 0,656419002 | 1,08E-02 | 1,00E+00 |
| IgG2_Sialylation | Under5Mort | 0,095756562 | 7,45E-01 | 1,00E+00 |
| IgG2_Bisecting | Under5Mort | 0,534202452 | 4,91E-02 | 1,00E+00 |
| IgG4_Agalactosylation | Under5Mort | -0,318690201 | 2,67E-01 | 1,00E+00 |
| IgG4_Monogalactosylation | Under5Mort | 0,66574538 | 9,35E-03 | 1,00E+00 |
| IgG4_Digalactosylation | Under5Mort | 0,219735454 | 4,50E-01 | 1,00E+00 |
| IgG4_Sialylation | Under5Mort | -0,114031357 | 6,98E-01 | 1,00E+00 |
| IgG4_Bisecting | Under5Mort | 0,013005203 | 9,65E-01 | 1,00E+00 |
| IgG1_Agalactosylation | Nnmort | -0,715323835 | 4,03E-03 | 1,00E+00 |
| IgG1_Digalactosylation | Nnmort | 0,727820535 | 3,17E-03 | 1,00E+00 |
| IgG1_Sialylation | Nnmort | -0,606915915 | 2,14E-02 | 1,00E+00 |
| IgG1_Bisecting | Nnmort | 0,558862093 | 3,78E-02 | 1,00E+00 |
| IgG2_Agalactosylation | Nnmort | -0,646188088 | 1,25E-02 | 1,00E+00 |
| IgG2_Monogalactosylation | Nnmort | 0,732182185 | 2,91E-03 | 1,00E+00 |
| IgG2_Digalactosylation | Nnmort | 0,602425825 | 2,26E-02 | 1,00E+00 |
| IgG2_Sialylation | Nnmort | 0,054118823 | 8,54E-01 | 1,00E+00 |
| IgG2_Bisecting | Nnmort | 0,533005119 | 4,97E-02 | 1,00E+00 |
| IgG4_Agalactosylation | Nnmort | -0,229448008 | 4,30E-01 | 1,00E+00 |
| IgG4_Monogalactosylation | Nnmort | 0,600980357 | 2,30E-02 | 1,00E+00 |
| IgG4_Digalactosylation | Nnmort | 0,137171605 | 6,40E-01 | 1,00E+00 |
| IgG4_Sialylation | Nnmort | -0,187733241 | 5,20E-01 | 1,00E+00 |
| IgG4_Bisecting | Nnmort | 0,056595872 | 8,48E-01 | 1,00E+00 |
| IgG1_Agalactosylation | HIV | -0,461388721 | 9,68E-02 | 1,00E+00 |
| IgG1_Monogalactosylation | HIV | 0,599964338 | 2,33E-02 | 1,00E+00 |
| IgG1_Digalactosylation | HIV | 0,418003706 | 1,37E-01 | 1,00E+00 |
| IgG1_Sialylation | HIV | -0,454826608 | 1,02E-01 | 1,00E+00 |
| IgG1_Bisecting | HIV | 0,688790351 | 6,44E-03 | 1,00E+00 |
| IgG2_Agalactosylation | HIV | -0,275785121 | 3,40E-01 | 1,00E+00 |
| IgG2_Monogalactosylation | HIV | 0,439740809 | 1,16E-01 | 1,00E+00 |
| IgG2_Digalactosylation | HIV | 0,194577069 | 5,05E-01 | 1,00E+00 |
| IgG2_Sialylation | HIV | -0,157795771 | 5,90E-01 | 1,00E+00 |
| IgG2_Bisecting | HIV | 0,662516552 | 9,83E-03 | 1,00E+00 |
| IgG4_Agalactosylation | HIV | 0,036314938 | 9,02E-01 | 1,00E+00 |
| IgG4_Monogalactosylation | HIV | 0,318221483 | 2,68E-01 | 1,00E+00 |
| IgG4_Digalactosylation | HIV | -0,170423567 | 5,60E-01 | 1,00E+00 |
| IgG4_Sialylation | HIV | -0,182679967 | 5,32E-01 | 1,00E+00 |
| IgG4_Bisecting | HIV | 0,401165555 | 1,55E-01 | 1,00E+00 |
| IgG1_Agalactosylation | Tuberculosis | -0,603069247 | 2,24E-02 | 1,00E+00 |
| IgG1_Monogalactosylation | Tuberculosis | 0,76203146 | 1,54E-03 | 1,00E+00 |
| IgG1_Digalactosylation | Tuberculosis | 0,517171826 | 5,82E-02 | 1,00E+00 |
| IgG1_Sialylation | Tuberculosis | -0,490455499 | 7,50E-02 | 1,00E+00 |
| IgG1_Bisecting | Tuberculosis | 0,524246562 | 5,43E-02 | 1,00E+00 |
| IgG2_Agalactosylation | Tuberculosis | -0,442737669 | 1,13E-01 | 1,00E+00 |
| IgG2_Monogalactosylation | Tuberculosis | 0,665653408 | 9,36E-03 | 1,00E+00 |
| IgG2_Digalactosylation | Tuberculosis | 0,310149244 | 2,81E-01 | 1,00E+00 |
| IgG2_Sialylation | Tuberculosis | -0,167224 | 5,68E-01 | 1,00E+00 |
| IgG2_Bisecting | Tuberculosis | 0,493822105 | 7,27E-02 | 1,00E+00 |
| IgG4_Agalactosylation | Tuberculosis | -0,004363498 | 9,88E-01 | 1,00E+00 |
| IgG4_Monogalactosylation | Tuberculosis | 0,484600794 | 7,91E-02 | 1,00E+00 |
| IgG4_Digalactosylation | Tuberculosis | -0,169672041 | 5,62E-01 | 1,00E+00 |
| IgG4_Sialylation | Tuberculosis | -0,30977615 | 2,81E-01 | 1,00E+00 |
| IgG4_Bisecting | Tuberculosis | 0,308524401 | 2,83E-01 | 1,00E+00 |
| IgG1_Agalactosylation | Malaria | -0,107121449 | 7,15E-01 | 1,00E+00 |
| IgG1_Monogalactosylation | Malaria | 0,147136165 | 6,16E-01 | 1,00E+00 |
| IgG1_Digalactosylation | Malaria | 0,089636204 | 7,61E-01 | 1,00E+00 |
| IgG1_Sialylation | Malaria | -0,125945371 | 6,68E-01 | 1,00E+00 |
| IgG1_Bisecting | Malaria | 0,415959578 | 1,39E-01 | 1,00E+00 |
| IgG2_Agalactosylation | Malaria | 0,01787872 | 9,52E-01 | 1,00E+00 |
| IgG2_Monogalactosylation | Malaria | -0,059131692 | 8,41E-01 | 1,00E+00 |
| IgG2_Digalactosylation | Malaria | -0,029620599 | 9,20E-01 | 1,00E+00 |
| IgG2_Sialylation | Malaria | 0,135425653 | 6,44E-01 | 1,00E+00 |
| IgG2_Bisecting | Malaria | 0,411093709 | 1,44E-01 | 1,00E+00 |
| IgG4_Agalactosylation | Malaria | -0,053910439 | 8,55E-01 | 1,00E+00 |
| IgG4_Monogalactosylation | Malaria | 0,069863161 | 8,12E-01 | 1,00E+00 |
| IgG4_Digalactosylation | Malaria | -0,002788222 | 9,92E-01 | 1,00E+00 |
| IgG4_Sialylation | Malaria | 0,097345335 | 7,41E-01 | 1,00E+00 |
| IgG4_Bisecting | Malaria | 0,227295177 | 4,35E-01 | 1,00E+00 |
| IgG1_Digalactosylation | HepatB | 0,690722326 | 6,24E-03 | 1,00E+00 |
| IgG1_Sialylation | HepatB | -0,313363699 | 2,75E-01 | 1,00E+00 |
| IgG1_Bisecting | HepatB | 0,492759131 | 7,34E-02 | 1,00E+00 |
| IgG2_Agalactosylation | HepatB | -0,61673081 | 1,88E-02 | 1,00E+00 |
| IgG2_Digalactosylation | HepatB | 0,4293719 | 1,25E-01 | 1,00E+00 |
| IgG2_Sialylation | HepatB | 0,122767741 | 6,76E-01 | 1,00E+00 |
| IgG2_Bisecting | HepatB | 0,368579372 | 1,95E-01 | 1,00E+00 |
| IgG4_Agalactosylation | HepatB | -0,304482452 | 2,90E-01 | 1,00E+00 |
| IgG4_Monogalactosylation | HepatB | 0,699305953 | 5,38E-03 | 1,00E+00 |
| IgG4_Digalactosylation | HepatB | 0,104314697 | 7,23E-01 | 1,00E+00 |
| IgG4_Sialylation | HepatB | -0,065251782 | 8,25E-01 | 1,00E+00 |
| IgG4_Bisecting | HepatB | 0,156866034 | 5,92E-01 | 1,00E+00 |
| IgG1_Agalactosylation | NTDs | 0,003983452 | 9,89E-01 | 1,00E+00 |
| IgG1_Monogalactosylation | NTDs | 0,025072478 | 9,32E-01 | 1,00E+00 |
| IgG1_Digalactosylation | NTDs | -0,020147237 | 9,45E-01 | 1,00E+00 |
| IgG1_Sialylation | NTDs | -0,046904279 | 8,73E-01 | 1,00E+00 |
| IgG1_Bisecting | NTDs | 0,332299083 | 2,46E-01 | 1,00E+00 |
| IgG2_Agalactosylation | NTDs | 0,095570966 | 7,45E-01 | 1,00E+00 |
| IgG2_Monogalactosylation | NTDs | -0,166334821 | 5,70E-01 | 1,00E+00 |
| IgG2_Digalactosylation | NTDs | -0,10243172 | 7,28E-01 | 1,00E+00 |
| IgG2_Sialylation | NTDs | 0,180646576 | 5,37E-01 | 1,00E+00 |
| IgG2_Bisecting | NTDs | 0,344380425 | 2,28E-01 | 1,00E+00 |
| IgG4_Agalactosylation | NTDs | -0,028134675 | 9,24E-01 | 1,00E+00 |
| IgG4_Monogalactosylation | NTDs | -0,017998302 | 9,51E-01 | 1,00E+00 |
| IgG4_Digalactosylation | NTDs | -0,006793718 | 9,82E-01 | 1,00E+00 |
| IgG4_Sialylation | NTDs | 0,122593774 | 6,76E-01 | 1,00E+00 |
| IgG4_Bisecting | NTDs | 0,205752388 | 4,80E-01 | 1,00E+00 |
| IgG1_Agalactosylation | NCDs | -0,763208648 | 1,49E-03 | 1,00E+00 |
| IgG1_Digalactosylation | NCDs | 0,709659459 | 4,47E-03 | 1,00E+00 |
| IgG1_Sialylation | NCDs | -0,494615449 | 7,22E-02 | 1,00E+00 |
| IgG1_Bisecting | NCDs | 0,611930921 | 2,00E-02 | 1,00E+00 |
| IgG2_Agalactosylation | NCDs | -0,718087966 | 3,82E-03 | 1,00E+00 |
| IgG2_Digalactosylation | NCDs | 0,572247718 | 3,25E-02 | 1,00E+00 |
| IgG2_Sialylation | NCDs | 0,278644748 | 3,35E-01 | 1,00E+00 |
| IgG2_Bisecting | NCDs | 0,533132994 | 4,96E-02 | 1,00E+00 |
| IgG4_Agalactosylation | NCDs | -0,405769059 | 1,50E-01 | 1,00E+00 |
| IgG4_Monogalactosylation | NCDs | 0,679253371 | 7,55E-03 | 1,00E+00 |
| IgG4_Digalactosylation | NCDs | 0,282577889 | 3,28E-01 | 1,00E+00 |
| IgG4_Sialylation | NCDs | -0,01516047 | 9,59E-01 | 1,00E+00 |
| IgG4_Bisecting | NCDs | 0,144924452 | 6,21E-01 | 1,00E+00 |
| IgG1_Agalactosylation | Suicide | -0,28838536 | 3,17E-01 | 1,00E+00 |
| IgG1_Monogalactosylation | Suicide | 0,383192902 | 1,76E-01 | 1,00E+00 |
| IgG1_Digalactosylation | Suicide | 0,281059652 | 3,30E-01 | 1,00E+00 |
| IgG1_Sialylation | Suicide | -0,426197376 | 1,29E-01 | 1,00E+00 |
| IgG1_Bisecting | Suicide | 0,354301464 | 2,14E-01 | 1,00E+00 |
| IgG2_Agalactosylation | Suicide | -0,293529669 | 3,08E-01 | 1,00E+00 |
| IgG2_Monogalactosylation | Suicide | 0,366141401 | 1,98E-01 | 1,00E+00 |
| IgG2_Digalactosylation | Suicide | 0,230680994 | 4,28E-01 | 1,00E+00 |
| IgG2_Sialylation | Suicide | -0,088711598 | 7,63E-01 | 1,00E+00 |
| IgG2_Bisecting | Suicide | 0,397810584 | 1,59E-01 | 1,00E+00 |
| IgG4_Agalactosylation | Suicide | 0,035279648 | 9,05E-01 | 1,00E+00 |
| IgG4_Monogalactosylation | Suicide | 0,191424793 | 5,12E-01 | 1,00E+00 |
| IgG4_Digalactosylation | Suicide | -0,099371818 | 7,35E-01 | 1,00E+00 |
| IgG4_Sialylation | Suicide | -0,247002195 | 3,95E-01 | 1,00E+00 |
| IgG4_Bisecting | Suicide | 0,319975306 | 2,65E-01 | 1,00E+00 |
| IgG1_Agalactosylation | Alcohol | 0,397379917 | 1,59E-01 | 1,00E+00 |
| IgG1_Monogalactosylation | Alcohol | -0,329454176 | 2,50E-01 | 1,00E+00 |
| IgG1_Digalactosylation | Alcohol | -0,317509086 | 2,69E-01 | 1,00E+00 |
| IgG1_Sialylation | Alcohol | -0,083148757 | 7,77E-01 | 1,00E+00 |
| IgG1_Bisecting | Alcohol | -0,257377094 | 3,74E-01 | 1,00E+00 |
| IgG2_Agalactosylation | Alcohol | 0,25818445 | 3,73E-01 | 1,00E+00 |
| IgG2_Monogalactosylation | Alcohol | -0,242742822 | 4,03E-01 | 1,00E+00 |
| IgG2_Digalactosylation | Alcohol | -0,18911913 | 5,17E-01 | 1,00E+00 |
| IgG2_Sialylation | Alcohol | -0,273261169 | 3,45E-01 | 1,00E+00 |
| IgG2_Bisecting | Alcohol | -0,15540424 | 5,96E-01 | 1,00E+00 |
| IgG4_Agalactosylation | Alcohol | 0,382954552 | 1,77E-01 | 1,00E+00 |
| IgG4_Monogalactosylation | Alcohol | -0,374776358 | 1,87E-01 | 1,00E+00 |
| IgG4_Digalactosylation | Alcohol | -0,2912194 | 3,12E-01 | 1,00E+00 |
| IgG4_Sialylation | Alcohol | -0,292786116 | 3,10E-01 | 1,00E+00 |
| IgG4_Bisecting | Alcohol | -0,03195087 | 9,14E-01 | 1,00E+00 |
| IgG1_Agalactosylation | RoadInjuries | -0,724146439 | 3,40E-03 | 1,00E+00 |
| IgG1_Digalactosylation | RoadInjuries | 0,569398699 | 3,36E-02 | 1,00E+00 |
| IgG1_Sialylation | RoadInjuries | -0,310861245 | 2,79E-01 | 1,00E+00 |
| IgG1_Bisecting | RoadInjuries | 0,559478746 | 3,75E-02 | 1,00E+00 |
| IgG2_Agalactosylation | RoadInjuries | -0,467323306 | 9,20E-02 | 1,00E+00 |
| IgG2_Monogalactosylation | RoadInjuries | 0,647845416 | 1,22E-02 | 1,00E+00 |
| IgG2_Digalactosylation | RoadInjuries | 0,27558139 | 3,40E-01 | 1,00E+00 |
| IgG2_Sialylation | RoadInjuries | 0,062064223 | 8,33E-01 | 1,00E+00 |
| IgG2_Bisecting | RoadInjuries | 0,484967013 | 7,88E-02 | 1,00E+00 |
| IgG4_Agalactosylation | RoadInjuries | -0,114322383 | 6,97E-01 | 1,00E+00 |
| IgG4_Monogalactosylation | RoadInjuries | 0,538160679 | 4,71E-02 | 1,00E+00 |
| IgG4_Digalactosylation | RoadInjuries | -0,09187628 | 7,55E-01 | 1,00E+00 |
| IgG4_Sialylation | RoadInjuries | -0,150732945 | 6,07E-01 | 1,00E+00 |
| IgG4_Bisecting | RoadInjuries | 0,295185425 | 3,06E-01 | 1,00E+00 |
| IgG1_Agalactosylation | FPneedMet | -0,631416341 | 1,54E-02 | 1,00E+00 |
| IgG1_Monogalactosylation | FPneedMet | 0,677482759 | 7,77E-03 | 1,00E+00 |
| IgG1_Digalactosylation | FPneedMet | 0,657751769 | 1,06E-02 | 1,00E+00 |
| IgG1_Sialylation | FPneedMet | -0,485834436 | 7,82E-02 | 1,00E+00 |
| IgG1_Bisecting | FPneedMet | 0,086512634 | 7,69E-01 | 1,00E+00 |
| IgG2_Agalactosylation | FPneedMet | -0,61524581 | 1,92E-02 | 1,00E+00 |
| IgG2_Monogalactosylation | FPneedMet | 0,705505521 | 4,82E-03 | 1,00E+00 |
| IgG2_Digalactosylation | FPneedMet | 0,589614418 | 2,65E-02 | 1,00E+00 |
| IgG2_Sialylation | FPneedMet | -0,054212841 | 8,54E-01 | 1,00E+00 |
| IgG2_Bisecting | FPneedMet | 0,017824051 | 9,52E-01 | 1,00E+00 |
| IgG4_Agalactosylation | FPneedMet | -0,214637897 | 4,61E-01 | 1,00E+00 |
| IgG4_Monogalactosylation | FPneedMet | 0,576068283 | 3,11E-02 | 1,00E+00 |
| IgG4_Digalactosylation | FPneedMet | 0,157424438 | 5,91E-01 | 1,00E+00 |
| IgG4_Sialylation | FPneedMet | -0,275092581 | 3,41E-01 | 1,00E+00 |
| IgG4_Bisecting | FPneedMet | -0,287801317 | 3,18E-01 | 1,00E+00 |
| IgG1_Agalactosylation | AdolBirthRate | -0,740408874 | 2,46E-03 | 1,00E+00 |
| IgG1_Digalactosylation | AdolBirthRate | 0,747298947 | 2,13E-03 | 1,00E+00 |
| IgG1_Sialylation | AdolBirthRate | -0,668919108 | 8,90E-03 | 1,00E+00 |
| IgG1_Bisecting | AdolBirthRate | 0,595527048 | 2,46E-02 | 1,00E+00 |
| IgG2_Agalactosylation | AdolBirthRate | -0,599888648 | 2,33E-02 | 1,00E+00 |
| IgG2_Digalactosylation | AdolBirthRate | 0,559378586 | 3,75E-02 | 1,00E+00 |
| IgG2_Sialylation | AdolBirthRate | -0,183342184 | 5,30E-01 | 1,00E+00 |
| IgG2_Bisecting | AdolBirthRate | 0,533989531 | 4,92E-02 | 1,00E+00 |
| IgG4_Agalactosylation | AdolBirthRate | -0,164733276 | 5,74E-01 | 1,00E+00 |
| IgG4_Monogalactosylation | AdolBirthRate | 0,633020849 | 1,51E-02 | 1,00E+00 |
| IgG4_Digalactosylation | AdolBirthRate | 0,035947295 | 9,03E-01 | 1,00E+00 |
| IgG4_Sialylation | AdolBirthRate | -0,264762893 | 3,60E-01 | 1,00E+00 |
| IgG4_Bisecting | AdolBirthRate | 0,161430215 | 5,81E-01 | 1,00E+00 |
| IgG1_Sialylation | UHCTracer | -0,347857289 | 2,23E-01 | 1,00E+00 |
| IgG1_Bisecting | UHCTracer | 0,596825453 | 2,42E-02 | 1,00E+00 |
| IgG2_Digalactosylation | UHCTracer | 0,611301378 | 2,02E-02 | 1,00E+00 |
| IgG2_Sialylation | UHCTracer | 0,284858144 | 3,24E-01 | 1,00E+00 |
| IgG2_Bisecting | UHCTracer | 0,416957 | 1,38E-01 | 1,00E+00 |
| IgG4_Agalactosylation | UHCTracer | -0,567544362 | 3,43E-02 | 1,00E+00 |
| IgG4_Digalactosylation | UHCTracer | 0,390450897 | 1,68E-01 | 1,00E+00 |
| IgG4_Sialylation | UHCTracer | 0,133111681 | 6,50E-01 | 1,00E+00 |
| IgG4_Bisecting | UHCTracer | 0,043153528 | 8,84E-01 | 1,00E+00 |
| IgG1_Sialylation | AirPollMort | -0,381918984 | 1,78E-01 | 1,00E+00 |
| IgG1_Bisecting | AirPollMort | 0,564891819 | 3,53E-02 | 1,00E+00 |
| IgG2_Agalactosylation | AirPollMort | -0,738456494 | 2,56E-03 | 1,00E+00 |
| IgG2_Digalactosylation | AirPollMort | 0,584565427 | 2,81E-02 | 1,00E+00 |
| IgG2_Sialylation | AirPollMort | 0,163921308 | 5,76E-01 | 1,00E+00 |
| IgG2_Bisecting | AirPollMort | 0,431257539 | 1,24E-01 | 1,00E+00 |
| IgG4_Agalactosylation | AirPollMort | -0,425785871 | 1,29E-01 | 1,00E+00 |
| IgG4_Monogalactosylation | AirPollMort | 0,748217385 | 2,08E-03 | 1,00E+00 |
| IgG4_Digalactosylation | AirPollMort | 0,263719385 | 3,62E-01 | 1,00E+00 |
| IgG4_Sialylation | AirPollMort | 0,029099193 | 9,21E-01 | 1,00E+00 |
| IgG4_Bisecting | AirPollMort | -0,008167816 | 9,78E-01 | 1,00E+00 |
| IgG1_Sialylation | WaSHMort | -0,611132604 | 2,02E-02 | 1,00E+00 |
| IgG2_Agalactosylation | WaSHMort | -0,652927641 | 1,14E-02 | 1,00E+00 |
| IgG2_Monogalactosylation | WaSHMort | 0,746175121 | 2,18E-03 | 1,00E+00 |
| IgG2_Digalactosylation | WaSHMort | 0,596180444 | 2,44E-02 | 1,00E+00 |
| IgG2_Sialylation | WaSHMort | 0,01726152 | 9,53E-01 | 1,00E+00 |
| IgG2_Bisecting | WaSHMort | 0,698411923 | 5,46E-03 | 1,00E+00 |
| IgG4_Agalactosylation | WaSHMort | -0,372968011 | 1,89E-01 | 1,00E+00 |
| IgG4_Monogalactosylation | WaSHMort | 0,687089871 | 6,63E-03 | 1,00E+00 |
| IgG4_Digalactosylation | WaSHMort | 0,235928752 | 4,17E-01 | 1,00E+00 |
| IgG4_Sialylation | WaSHMort | -0,010438687 | 9,72E-01 | 1,00E+00 |
| IgG4_Bisecting | WaSHMort | 0,242987966 | 4,03E-01 | 1,00E+00 |
| IgG1_Agalactosylation | Poisons | -0,648514893 | 1,21E-02 | 1,00E+00 |
| IgG1_Monogalactosylation | Poisons | 0,743496698 | 2,30E-03 | 1,00E+00 |
| IgG1_Digalactosylation | Poisons | 0,597785187 | 2,40E-02 | 1,00E+00 |
| IgG1_Sialylation | Poisons | -0,440526145 | 1,15E-01 | 1,00E+00 |
| IgG1_Bisecting | Poisons | 0,678458832 | 7,64E-03 | 1,00E+00 |
| IgG2_Agalactosylation | Poisons | -0,558160794 | 3,80E-02 | 1,00E+00 |
| IgG2_Monogalactosylation | Poisons | 0,670467914 | 8,69E-03 | 1,00E+00 |
| IgG2_Digalactosylation | Poisons | 0,436428493 | 1,19E-01 | 1,00E+00 |
| IgG2_Sialylation | Poisons | 0,092499433 | 7,53E-01 | 1,00E+00 |
| IgG2_Bisecting | Poisons | 0,582413369 | 2,89E-02 | 1,00E+00 |
| IgG4_Agalactosylation | Poisons | -0,243672541 | 4,01E-01 | 1,00E+00 |
| IgG4_Monogalactosylation | Poisons | 0,615992284 | 1,90E-02 | 1,00E+00 |
| IgG4_Digalactosylation | Poisons | 0,089943449 | 7,60E-01 | 1,00E+00 |
| IgG4_Sialylation | Poisons | -0,108035979 | 7,13E-01 | 1,00E+00 |
| IgG4_Bisecting | Poisons | 0,189252866 | 5,17E-01 | 1,00E+00 |
| IgG1_Agalactosylation | Smoking | -0,099933896 | 7,34E-01 | 1,00E+00 |
| IgG1_Monogalactosylation | Smoking | -0,046616626 | 8,74E-01 | 1,00E+00 |
| IgG1_Digalactosylation | Smoking | 0,013546408 | 9,63E-01 | 1,00E+00 |
| IgG1_Sialylation | Smoking | 0,362673835 | 2,03E-01 | 1,00E+00 |
| IgG1_Bisecting | Smoking | -0,32246721 | 2,61E-01 | 1,00E+00 |
| IgG2_Agalactosylation | Smoking | -0,166154566 | 5,70E-01 | 1,00E+00 |
| IgG2_Monogalactosylation | Smoking | 0,058853238 | 8,42E-01 | 1,00E+00 |
| IgG2_Digalactosylation | Smoking | 0,041980921 | 8,87E-01 | 1,00E+00 |
| IgG2_Sialylation | Smoking | 0,501186823 | 6,79E-02 | 1,00E+00 |
| IgG2_Bisecting | Smoking | -0,46205343 | 9,62E-02 | 1,00E+00 |
| IgG4_Agalactosylation | Smoking | -0,431116956 | 1,24E-01 | 1,00E+00 |
| IgG4_Monogalactosylation | Smoking | 0,186566743 | 5,23E-01 | 1,00E+00 |
| IgG4_Digalactosylation | Smoking | 0,447683403 | 1,08E-01 | 1,00E+00 |
| IgG4_Sialylation | Smoking | 0,391761623 | 1,66E-01 | 1,00E+00 |
| IgG4_Bisecting | Smoking | -0,399004099 | 1,58E-01 | 1,00E+00 |
| IgG1_Agalactosylation | IPV | -0,419612264 | 1,35E-01 | 1,00E+00 |
| IgG1_Monogalactosylation | IPV | 0,489348659 | 7,57E-02 | 1,00E+00 |
| IgG1_Digalactosylation | IPV | 0,466933299 | 9,23E-02 | 1,00E+00 |
| IgG1_Sialylation | IPV | -0,380704071 | 1,79E-01 | 1,00E+00 |
| IgG1_Bisecting | IPV | 0,21145097 | 4,68E-01 | 1,00E+00 |
| IgG2_Agalactosylation | IPV | -0,233873776 | 4,21E-01 | 1,00E+00 |
| IgG2_Monogalactosylation | IPV | 0,368235122 | 1,95E-01 | 1,00E+00 |
| IgG2_Digalactosylation | IPV | 0,293465971 | 3,09E-01 | 1,00E+00 |
| IgG2_Sialylation | IPV | -0,326295967 | 2,55E-01 | 1,00E+00 |
| IgG2_Bisecting | IPV | 0,160831397 | 5,83E-01 | 1,00E+00 |
| IgG4_Agalactosylation | IPV | 0,078884319 | 7,89E-01 | 1,00E+00 |
| IgG4_Monogalactosylation | IPV | 0,249122518 | 3,90E-01 | 1,00E+00 |
| IgG4_Digalactosylation | IPV | -0,11848905 | 6,87E-01 | 1,00E+00 |
| IgG4_Sialylation | IPV | -0,260186921 | 3,69E-01 | 1,00E+00 |
| IgG4_Bisecting | IPV | -0,090335553 | 7,59E-01 | 1,00E+00 |
| IgG1_Agalactosylation | Water | -0,454339058 | 1,03E-01 | 1,00E+00 |
| IgG1_Monogalactosylation | Water | 0,431752919 | 1,23E-01 | 1,00E+00 |
| IgG1_Digalactosylation | Water | 0,414740925 | 1,40E-01 | 1,00E+00 |
| IgG1_Sialylation | Water | -0,13747466 | 6,39E-01 | 1,00E+00 |
| IgG1_Bisecting | Water | 0,078682325 | 7,89E-01 | 1,00E+00 |
| IgG2_Agalactosylation | Water | -0,33100302 | 2,48E-01 | 1,00E+00 |
| IgG2_Monogalactosylation | Water | 0,318844109 | 2,67E-01 | 1,00E+00 |
| IgG2_Digalactosylation | Water | 0,291060825 | 3,13E-01 | 1,00E+00 |
| IgG2_Sialylation | Water | 0,147575429 | 6,15E-01 | 1,00E+00 |
| IgG2_Bisecting | Water | 0,067696398 | 8,18E-01 | 1,00E+00 |
| IgG4_Agalactosylation | Water | -0,079974915 | 7,86E-01 | 1,00E+00 |
| IgG4_Monogalactosylation | Water | 0,235837663 | 4,17E-01 | 1,00E+00 |
| IgG4_Digalactosylation | Water | 0,034664051 | 9,06E-01 | 1,00E+00 |
| IgG4_Sialylation | Water | -0,108199571 | 7,13E-01 | 1,00E+00 |
| IgG4_Bisecting | Water | -0,059984675 | 8,39E-01 | 1,00E+00 |
| IgG1_Agalactosylation | Sanitation | -0,500133398 | 6,86E-02 | 1,00E+00 |
| IgG1_Monogalactosylation | Sanitation | 0,477524502 | 8,42E-02 | 1,00E+00 |
| IgG1_Digalactosylation | Sanitation | 0,374921373 | 1,87E-01 | 1,00E+00 |
| IgG1_Sialylation | Sanitation | 0,028572446 | 9,23E-01 | 1,00E+00 |
| IgG1_Bisecting | Sanitation | 0,176640513 | 5,46E-01 | 1,00E+00 |
| IgG2_Agalactosylation | Sanitation | -0,352436785 | 2,16E-01 | 1,00E+00 |
| IgG2_Monogalactosylation | Sanitation | 0,36153456 | 2,04E-01 | 1,00E+00 |
| IgG2_Digalactosylation | Sanitation | 0,227472913 | 4,34E-01 | 1,00E+00 |
| IgG2_Sialylation | Sanitation | 0,274291358 | 3,43E-01 | 1,00E+00 |
| IgG2_Bisecting | Sanitation | 0,179309324 | 5,40E-01 | 1,00E+00 |
| IgG4_Agalactosylation | Sanitation | -0,106223199 | 7,18E-01 | 1,00E+00 |
| IgG4_Monogalactosylation | Sanitation | 0,230733157 | 4,27E-01 | 1,00E+00 |
| IgG4_Digalactosylation | Sanitation | 0,005183696 | 9,86E-01 | 1,00E+00 |
| IgG4_Sialylation | Sanitation | 0,001487366 | 9,96E-01 | 1,00E+00 |
| IgG4_Bisecting | Sanitation | 0,027757414 | 9,25E-01 | 1,00E+00 |
| IgG1_Digalactosylation | Hygiene | 0,747485957 | 2,12E-03 | 1,00E+00 |
| IgG1_Sialylation | Hygiene | -0,450870212 | 1,06E-01 | 1,00E+00 |
| IgG1_Bisecting | Hygiene | 0,349683998 | 2,20E-01 | 1,00E+00 |
| IgG2_Agalactosylation | Hygiene | -0,68099141 | 7,34E-03 | 1,00E+00 |
| IgG2_Digalactosylation | Hygiene | 0,579158477 | 3,00E-02 | 1,00E+00 |
| IgG2_Sialylation | Hygiene | 0,031370797 | 9,15E-01 | 1,00E+00 |
| IgG2_Bisecting | Hygiene | 0,282419789 | 3,28E-01 | 1,00E+00 |
| IgG4_Agalactosylation | Hygiene | -0,256146624 | 3,77E-01 | 1,00E+00 |
| IgG4_Monogalactosylation | Hygiene | 0,666548835 | 9,23E-03 | 1,00E+00 |
| IgG4_Digalactosylation | Hygiene | 0,119961694 | 6,83E-01 | 1,00E+00 |
| IgG4_Sialylation | Hygiene | -0,188565215 | 5,19E-01 | 1,00E+00 |
| IgG4_Bisecting | Hygiene | -0,06214232 | 8,33E-01 | 1,00E+00 |
| IgG1_Agalactosylation | HHAirPoll | -0,720442239 | 3,66E-03 | 1,00E+00 |
| IgG1_Digalactosylation | HHAirPoll | 0,760921273 | 1,57E-03 | 1,00E+00 |
| IgG1_Sialylation | HHAirPoll | -0,532595223 | 4,99E-02 | 1,00E+00 |
| IgG1_Bisecting | HHAirPoll | 0,525572249 | 5,36E-02 | 1,00E+00 |
| IgG2_Agalactosylation | HHAirPoll | -0,63117229 | 1,55E-02 | 1,00E+00 |
| IgG2_Monogalactosylation | HHAirPoll | 0,748424795 | 2,08E-03 | 1,00E+00 |
| IgG2_Digalactosylation | HHAirPoll | 0,631430596 | 1,54E-02 | 1,00E+00 |
| IgG2_Sialylation | HHAirPoll | -0,13154409 | 6,54E-01 | 1,00E+00 |
| IgG2_Bisecting | HHAirPoll | 0,451423307 | 1,05E-01 | 1,00E+00 |
| IgG4_Agalactosylation | HHAirPoll | -0,25040777 | 3,88E-01 | 1,00E+00 |
| IgG4_Monogalactosylation | HHAirPoll | 0,582940986 | 2,87E-02 | 1,00E+00 |
| IgG4_Digalactosylation | HHAirPoll | 0,15971854 | 5,85E-01 | 1,00E+00 |
| IgG4_Sialylation | HHAirPoll | -0,114732718 | 6,96E-01 | 1,00E+00 |
| IgG4_Bisecting | HHAirPoll | -0,10805427 | 7,13E-01 | 1,00E+00 |
| IgG1_Sialylation | OccRiskBurden | -0,310366031 | 2,80E-01 | 1,00E+00 |
| IgG1_Bisecting | OccRiskBurden | 0,636037041 | 1,45E-02 | 1,00E+00 |
| IgG2_Digalactosylation | OccRiskBurden | 0,660271401 | 1,02E-02 | 1,00E+00 |
| IgG2_Sialylation | OccRiskBurden | 0,332632221 | 2,45E-01 | 1,00E+00 |
| IgG2_Bisecting | OccRiskBurden | 0,452935795 | 1,04E-01 | 1,00E+00 |
| IgG4_Agalactosylation | OccRiskBurden | -0,634120283 | 1,49E-02 | 1,00E+00 |
| IgG4_Digalactosylation | OccRiskBurden | 0,473461278 | 8,73E-02 | 1,00E+00 |
| IgG4_Sialylation | OccRiskBurden | 0,233319001 | 4,22E-01 | 1,00E+00 |
| IgG4_Bisecting | OccRiskBurden | -0,060478231 | 8,37E-01 | 1,00E+00 |
| IgG1_Agalactosylation | MeanPM25 | -0,352606175 | 2,16E-01 | 1,00E+00 |
| IgG1_Monogalactosylation | MeanPM25 | 0,441458504 | 1,14E-01 | 1,00E+00 |
| IgG1_Digalactosylation | MeanPM25 | 0,22900934 | 4,31E-01 | 1,00E+00 |
| IgG1_Sialylation | MeanPM25 | -0,012648481 | 9,66E-01 | 1,00E+00 |
| IgG1_Bisecting | MeanPM25 | -0,010973801 | 9,70E-01 | 1,00E+00 |
| IgG2_Agalactosylation | MeanPM25 | -0,151209958 | 6,06E-01 | 1,00E+00 |
| IgG2_Monogalactosylation | MeanPM25 | 0,386065459 | 1,73E-01 | 1,00E+00 |
| IgG2_Digalactosylation | MeanPM25 | 0,047932004 | 8,71E-01 | 1,00E+00 |
| IgG2_Sialylation | MeanPM25 | -0,227502754 | 4,34E-01 | 1,00E+00 |
| IgG2_Bisecting | MeanPM25 | -0,031764367 | 9,14E-01 | 1,00E+00 |
| IgG4_Agalactosylation | MeanPM25 | 0,154811764 | 5,97E-01 | 1,00E+00 |
| IgG4_Monogalactosylation | MeanPM25 | 0,175404906 | 5,49E-01 | 1,00E+00 |
| IgG4_Digalactosylation | MeanPM25 | -0,277974539 | 3,36E-01 | 1,00E+00 |
| IgG4_Sialylation | MeanPM25 | -0,21534634 | 4,60E-01 | 1,00E+00 |
| IgG4_Bisecting | MeanPM25 | -0,100472943 | 7,33E-01 | 1,00E+00 |
| IgG1_Agalactosylation | Violece | -0,500811625 | 6,81E-02 | 1,00E+00 |
| IgG1_Monogalactosylation | Violece | 0,632302075 | 1,53E-02 | 1,00E+00 |
| IgG1_Digalactosylation | Violece | 0,427965071 | 1,27E-01 | 1,00E+00 |
| IgG1_Sialylation | Violece | -0,467387133 | 9,20E-02 | 1,00E+00 |
| IgG1_Bisecting | Violece | 0,46685683 | 9,24E-02 | 1,00E+00 |
| IgG2_Agalactosylation | Violece | -0,35769412 | 2,09E-01 | 1,00E+00 |
| IgG2_Monogalactosylation | Violece | 0,483475635 | 7,99E-02 | 1,00E+00 |
| IgG2_Digalactosylation | Violece | 0,239811099 | 4,09E-01 | 1,00E+00 |
| IgG2_Sialylation | Violece | -0,0075278 | 9,80E-01 | 1,00E+00 |
| IgG2_Bisecting | Violece | 0,480446822 | 8,21E-02 | 1,00E+00 |
| IgG4_Agalactosylation | Violece | 0,05151224 | 8,61E-01 | 1,00E+00 |
| IgG4_Monogalactosylation | Violece | 0,359806799 | 2,06E-01 | 1,00E+00 |
| IgG4_Digalactosylation | Violece | -0,179675001 | 5,39E-01 | 1,00E+00 |
| IgG4_Sialylation | Violece | -0,335928661 | 2,40E-01 | 1,00E+00 |
| IgG4_Bisecting | Violece | 0,409614134 | 1,46E-01 | 1,00E+00 |
| IgG1_Agalactosylation | War | -0,059882122 | 8,39E-01 | 1,00E+00 |
| IgG1_Monogalactosylation | War | 0,065298588 | 8,24E-01 | 1,00E+00 |
| IgG1_Digalactosylation | War | 0,029698473 | 9,20E-01 | 1,00E+00 |
| IgG1_Sialylation | War | 0,050331837 | 8,64E-01 | 1,00E+00 |
| IgG1_Bisecting | War | -0,401681394 | 1,55E-01 | 1,00E+00 |
| IgG2_Agalactosylation | War | -0,055065677 | 8,52E-01 | 1,00E+00 |
| IgG2_Monogalactosylation | War | 0,098731137 | 7,37E-01 | 1,00E+00 |
| IgG2_Digalactosylation | War | 0,024849879 | 9,33E-01 | 1,00E+00 |
| IgG2_Sialylation | War | 0,022279441 | 9,40E-01 | 1,00E+00 |
| IgG2_Bisecting | War | -0,443485664 | 1,12E-01 | 1,00E+00 |
| IgG4_Agalactosylation | War | 0,059622545 | 8,40E-01 | 1,00E+00 |
| IgG4_Monogalactosylation | War | 0,07788774 | 7,91E-01 | 1,00E+00 |
| IgG4_Digalactosylation | War | -0,041798468 | 8,87E-01 | 1,00E+00 |
| IgG4_Sialylation | War | -0,194037949 | 5,06E-01 | 1,00E+00 |
| IgG4_Bisecting | War | -0,266306289 | 3,57E-01 | 1,00E+00 |
| IgG1_Sialylation | MDG | -0,559063328 | 3,77E-02 | 1,00E+00 |
| IgG1_Bisecting | MDG | 0,649044094 | 1,20E-02 | 1,00E+00 |
| IgG2_Agalactosylation | MDG | -0,752689382 | 1,89E-03 | 1,00E+00 |
| IgG2_Digalactosylation | MDG | 0,653583565 | 1,12E-02 | 1,00E+00 |
| IgG2_Sialylation | MDG | 0,09728731 | 7,41E-01 | 1,00E+00 |
| IgG2_Bisecting | MDG | 0,54147241 | 4,55E-02 | 1,00E+00 |
| IgG4_Agalactosylation | MDG | -0,38532966 | 1,74E-01 | 1,00E+00 |
| IgG4_Digalactosylation | MDG | 0,235367786 | 4,18E-01 | 1,00E+00 |
| IgG4_Sialylation | MDG | -0,074162216 | 8,01E-01 | 1,00E+00 |
| IgG4_Bisecting | MDG | 0,074469515 | 8,00E-01 | 1,00E+00 |
| IgG1_Digalactosylation | Non.MDG | 0,763586031 | 1,48E-03 | 1,00E+00 |
| IgG1_Sialylation | Non.MDG | -0,39564521 | 1,61E-01 | 1,00E+00 |
| IgG1_Bisecting | Non.MDG | 0,444662768 | 1,11E-01 | 1,00E+00 |
| IgG2_Agalactosylation | Non.MDG | -0,669538467 | 8,81E-03 | 1,00E+00 |
| IgG2_Digalactosylation | Non.MDG | 0,541265208 | 4,56E-02 | 1,00E+00 |
| IgG2_Sialylation | Non.MDG | 0,072354456 | 8,06E-01 | 1,00E+00 |
| IgG2_Bisecting | Non.MDG | 0,319796199 | 2,65E-01 | 1,00E+00 |
| IgG4_Agalactosylation | Non.MDG | -0,288542102 | 3,17E-01 | 1,00E+00 |
| IgG4_Monogalactosylation | Non.MDG | 0,684048278 | 6,98E-03 | 1,00E+00 |
| IgG4_Digalactosylation | Non.MDG | 0,139539727 | 6,34E-01 | 1,00E+00 |
| IgG4_Sialylation | Non.MDG | -0,109674342 | 7,09E-01 | 1,00E+00 |
| IgG4_Bisecting | Non.MDG | -0,063397227 | 8,30E-01 | 1,00E+00 |
| IgG1_Sialylation | LifeExp | -0,662663994 | 9,80E-03 | 1,00E+00 |
| IgG1_Bisecting | LifeExp | 0,614494663 | 1,94E-02 | 1,00E+00 |
| IgG2_Agalactosylation | LifeExp | -0,71684363 | 3,91E-03 | 1,00E+00 |
| IgG2_Digalactosylation | LifeExp | 0,655658929 | 1,09E-02 | 1,00E+00 |
| IgG2_Sialylation | LifeExp | 0,020279666 | 9,45E-01 | 1,00E+00 |
| IgG2_Bisecting | LifeExp | 0,560632817 | 3,70E-02 | 1,00E+00 |
| IgG4_Agalactosylation | LifeExp | -0,267980642 | 3,54E-01 | 1,00E+00 |
| IgG4_Monogalactosylation | LifeExp | 0,666783746 | 9,20E-03 | 1,00E+00 |
| IgG4_Digalactosylation | LifeExp | 0,15831053 | 5,89E-01 | 1,00E+00 |
| IgG4_Sialylation | LifeExp | -0,192767766 | 5,09E-01 | 1,00E+00 |
| IgG4_Bisecting | LifeExp | 0,076453001 | 7,95E-01 | 1,00E+00 |
| IgG1_Sialylation | LifeExp_F | -0,662438241 | 9,84E-03 | 1,00E+00 |
| IgG1_Bisecting | LifeExp_F | 0,66832596 | 8,98E-03 | 1,00E+00 |
| IgG2_Agalactosylation | LifeExp_F | -0,749277696 | 2,04E-03 | 1,00E+00 |
| IgG2_Digalactosylation | LifeExp_F | 0,695195443 | 5,78E-03 | 1,00E+00 |
| IgG2_Sialylation | LifeExp_F | 0,039962836 | 8,92E-01 | 1,00E+00 |
| IgG2_Bisecting | LifeExp_F | 0,607084012 | 2,13E-02 | 1,00E+00 |
| IgG4_Agalactosylation | LifeExp_F | -0,325427159 | 2,56E-01 | 1,00E+00 |
| IgG4_Monogalactosylation | LifeExp_F | 0,69788165 | 5,51E-03 | 1,00E+00 |
| IgG4_Digalactosylation | LifeExp_F | 0,214268566 | 4,62E-01 | 1,00E+00 |
| IgG4_Sialylation | LifeExp_F | -0,136057774 | 6,43E-01 | 1,00E+00 |
| IgG4_Bisecting | LifeExp_F | 0,070999032 | 8,09E-01 | 1,00E+00 |
| IgG1_Agalactosylation | LifeExp_M | -0,759156802 | 1,64E-03 | 1,00E+00 |
| IgG1_Digalactosylation | LifeExp_M | 0,75944531 | 1,63E-03 | 1,00E+00 |
| IgG1_Sialylation | LifeExp_M | -0,64849518 | 1,21E-02 | 1,00E+00 |
| IgG1_Bisecting | LifeExp_M | 0,553120978 | 4,02E-02 | 1,00E+00 |
| IgG2_Agalactosylation | LifeExp_M | -0,678035087 | 7,70E-03 | 1,00E+00 |
| IgG2_Digalactosylation | LifeExp_M | 0,608383469 | 2,10E-02 | 1,00E+00 |
| IgG2_Sialylation | LifeExp_M | 0,01312646 | 9,64E-01 | 1,00E+00 |
| IgG2_Bisecting | LifeExp_M | 0,505541209 | 6,52E-02 | 1,00E+00 |
| IgG4_Agalactosylation | LifeExp_M | -0,218670542 | 4,53E-01 | 1,00E+00 |
| IgG4_Monogalactosylation | LifeExp_M | 0,63055583 | 1,56E-02 | 1,00E+00 |
| IgG4_Digalactosylation | LifeExp_M | 0,112452061 | 7,02E-01 | 1,00E+00 |
| IgG4_Sialylation | LifeExp_M | -0,236036038 | 4,17E-01 | 1,00E+00 |
| IgG4_Bisecting | LifeExp_M | 0,083298089 | 7,77E-01 | 1,00E+00 |
| IgG1_Digalactosylation | GDP_2013 | 0,722327529 | 3,53E-03 | 1,00E+00 |
| IgG1_Sialylation | GDP_2013 | -0,370525677 | 1,92E-01 | 1,00E+00 |
| IgG1_Bisecting | GDP_2013 | 0,396319233 | 1,61E-01 | 1,00E+00 |
| IgG2_Agalactosylation | GDP_2013 | -0,660469461 | 1,01E-02 | 1,00E+00 |
| IgG2_Digalactosylation | GDP_2013 | 0,507510019 | 6,40E-02 | 1,00E+00 |
| IgG2_Sialylation | GDP_2013 | 0,041486819 | 8,88E-01 | 1,00E+00 |
| IgG2_Bisecting | GDP_2013 | 0,265866083 | 3,58E-01 | 1,00E+00 |
| IgG4_Agalactosylation | GDP_2013 | -0,294156887 | 3,07E-01 | 1,00E+00 |
| IgG4_Monogalactosylation | GDP_2013 | 0,726472538 | 3,25E-03 | 1,00E+00 |
| IgG4_Digalactosylation | GDP_2013 | 0,121055593 | 6,80E-01 | 1,00E+00 |
| IgG4_Sialylation | GDP_2013 | -0,136002315 | 6,43E-01 | 1,00E+00 |
| IgG4_Bisecting | GDP_2013 | -0,034072452 | 9,08E-01 | 1,00E+00 |
| IgG1_Sialylation | EduIndx_2014 | -0,383263188 | 1,76E-01 | 1,00E+00 |
| IgG1_Bisecting | EduIndx_2014 | 0,659262122 | 1,03E-02 | 1,00E+00 |
| IgG2_Agalactosylation | EduIndx_2014 | -0,695746356 | 5,72E-03 | 1,00E+00 |
| IgG2_Digalactosylation | EduIndx_2014 | 0,553948047 | 3,98E-02 | 1,00E+00 |
| IgG2_Sialylation | EduIndx_2014 | 0,206766445 | 4,78E-01 | 1,00E+00 |
| IgG2_Bisecting | EduIndx_2014 | 0,527698 | 5,25E-02 | 1,00E+00 |
| IgG4_Agalactosylation | EduIndx_2014 | -0,408763064 | 1,47E-01 | 1,00E+00 |
| IgG4_Digalactosylation | EduIndx_2014 | 0,225728967 | 4,38E-01 | 1,00E+00 |
| IgG4_Sialylation | EduIndx_2014 | 0,016480716 | 9,55E-01 | 1,00E+00 |
| IgG4_Bisecting | EduIndx_2014 | 0,096558068 | 7,43E-01 | 1,00E+00 |
| IgG1_Sialylation | HealthIndx_2014 | -0,662358154 | 9,85E-03 | 1,00E+00 |
| IgG1_Bisecting | HealthIndx_2014 | 0,615823045 | 1,90E-02 | 1,00E+00 |
| IgG2_Agalactosylation | HealthIndx_2014 | -0,716725057 | 3,92E-03 | 1,00E+00 |
| IgG2_Digalactosylation | HealthIndx_2014 | 0,655358606 | 1,10E-02 | 1,00E+00 |
| IgG2_Sialylation | HealthIndx_2014 | 0,020945002 | 9,43E-01 | 1,00E+00 |
| IgG2_Bisecting | HealthIndx_2014 | 0,562134208 | 3,64E-02 | 1,00E+00 |
| IgG4_Agalactosylation | HealthIndx_2014 | -0,268198259 | 3,54E-01 | 1,00E+00 |
| IgG4_Monogalactosylation | HealthIndx_2014 | 0,666583879 | 9,23E-03 | 1,00E+00 |
| IgG4_Digalactosylation | HealthIndx_2014 | 0,158356062 | 5,89E-01 | 1,00E+00 |
| IgG4_Sialylation | HealthIndx_2014 | -0,192002517 | 5,11E-01 | 1,00E+00 |
| IgG4_Bisecting | HealthIndx_2014 | 0,077687209 | 7,92E-01 | 1,00E+00 |
| IgG1_Sialylation | Water_UN | -0,379683953 | 1,81E-01 | 1,00E+00 |
| IgG2_Monogalactosylation | Water_UN | 0,751575387 | 1,94E-03 | 1,00E+00 |
| IgG2_Digalactosylation | Water_UN | 0,741306579 | 2,41E-03 | 1,00E+00 |
| IgG2_Sialylation | Water_UN | 0,556240275 | 3,89E-02 | 1,00E+00 |
| IgG2_Bisecting | Water_UN | 0,59416513 | 2,51E-02 | 1,00E+00 |
| IgG4_Digalactosylation | Water_UN | 0,704510245 | 4,91E-03 | 1,00E+00 |
| IgG4_Sialylation | Water_UN | 0,387514548 | 1,71E-01 | 1,00E+00 |
| IgG4_Bisecting | Water_UN | -0,043416056 | 8,83E-01 | 1,00E+00 |
| IgG1_Sialylation | Sanitation_UN | -0,507815664 | 6,38E-02 | 1,00E+00 |
| IgG1_Bisecting | Sanitation_UN | 0,728398612 | 3,13E-03 | 1,00E+00 |
| IgG2_Digalactosylation | Sanitation_UN | 0,713336362 | 4,18E-03 | 1,00E+00 |
| IgG2_Sialylation | Sanitation_UN | 0,238386729 | 4,12E-01 | 1,00E+00 |
| IgG2_Bisecting | Sanitation_UN | 0,59228991 | 2,56E-02 | 1,00E+00 |
| IgG4_Agalactosylation | Sanitation_UN | -0,520945372 | 5,61E-02 | 1,00E+00 |
| IgG4_Digalactosylation | Sanitation_UN | 0,409075318 | 1,46E-01 | 1,00E+00 |
| IgG4_Sialylation | Sanitation_UN | 0,101216901 | 7,31E-01 | 1,00E+00 |
| IgG4_Bisecting | Sanitation_UN | -0,057322838 | 8,46E-01 | 1,00E+00 |
