# Supplementary material for: Global variability of the human IgG glycome
Source: Aging (Albany NY). 2020 Aug 12;12(15):15222–59. doi: 10.18632/aging.103884 (PMC7467356; doi:10.18632/aging.103884)
Supplement: Supplementary Tables 11-13 [file aging-12-103884-s008..pdf]

SUPPLEMENTARY TABLES

Supplementary Table 11. Health-related Sustainable Development Goals (SDG) indicators used for assessment of country development level [65]. Development indicator and short descriptions are given.

Health-related SDG indicator	Definition
SDG	Overall health-related SDG index (health-related SDG indicators included)
MDG	Overall health-related MDX index (Health-related SDG indicators included in the Millennium Development Goals)
non-MDG	Health-related SDG indicators not included in the Millennium Development Goals
Adolescent birth rate	Birth rates for women aged 10–14 years and women aged 15–19 years, number of livebirths per 1000 women aged 10–14 years and women aged 15–19 years
Air pollution mortality	Age-standardised death rate attributable to household air pollution and ambient air pollution, per 100 000 population
Alcohol	Risk-weighted prevalence of alcohol consumption, as measured by the SEV for alcohol use, %
Disaster	Age-standardised death rate due to exposure to forces of nature, per 100 000 population
Family planning need met, modern contraception	Proportion of women of reproductive age (15–49 years) who have their need for family planning satisfied with modern methods, % women aged 15–49 years
Hepatitis B	Age-standardised rate of hepatitis B incidence, per 100 000 population
HIV	Age-standardised rate of new HIV infections, per 1000 population
Household air pollution	Risk-weighted prevalence of household air pollution, as measured by the SEV for household air pollution, %
Hygiene	Risk-weighted prevalence of populations with unsafe hygiene (no handwashing with soap), as measured by the SEV for unsafe hygiene, %
Intimate partner violence	Age-standardised prevalence of women aged 15 years and older who experienced intimate partner violence, %women aged 15 years and older
Malaria	Age-standardised rate of malaria cases, per 1000 population
Maternal mortality ratio	Maternal deaths per 100 000 livebirths
Mean PM 2.5	Population-weighted mean levels of PM2.5, $\mu g/m^3$
NCDs	Age-standardised death rate due to cardiovascular disease, cancer, diabetes, and chronic respiratory disease in populations aged 30 - 70 years, per 100 000 population
Neglected tropical diseases	Age-standardised prevalence of neglected tropical diseases, per 100 000 population
Neonatal mortality	Probability of dying during the first 28 days of life per 1000 livebirths
Occupational risk burden	Age-standardised all-cause DAILY rate attributable to occupational risks, per 100 000 population
Overweight	Prevalence of overweight in children aged 2 - 4 years, %
Poisons	Age-standardised death rate due to unintentional poisonings, per 100 000 population
Road injuries	Age-standardised death rate due to road traffic injuries, per 100 000 population
Sanitation	Risk-weighted prevalence of populations using unsafe or unimproved sanitation, as measured by the SEV for unsafe sanitation, %
Skilled birth attendance	Proportion of births attended by skilled health personnel (doctors, nurses, midwives, or country-specific medical staff [e.g., clinical officers]), %
Smoking	Age-standardised prevalence of daily smoking in populations aged 10 years and older, % population aged 10 years and older
Stunting	Prevalence of stunting in achieving, by 2025, the internationally agreed targets on children under age 5 years, %
Suicide	Age-standardised death rate due to self-harm, per 100 000 population
Tuberculosis	Age-standardised rate of new and relapsed tuberculosis cases, per 1000 population
Under-5 mortality	Probability of dying before age 5 years per 1000 livebirths
Universal health coverage tracer	Coverage of universal health coverage tracer interventions for prevention and treatment services, %
Violence	Age-standardised death rate due to interpersonal violence, per 100 000 population
War	Age-standardised death rate due to collective violence and legal intervention, per 100 000 population
WaSH mortality	Age-standardised death rate attributable to unsafe WaSH, per 100 000 population
Wasting	Prevalence of wasting in children under age 5 years, %
Water	Risk-weighted prevalence of populations using unsafe or unimproved water sources, as measured by the SEV for unsafe water, %

Supplementary Table 12. United Nation's Human Development Index (HDI) indicators used for assessment of country development level [66].

HDI indicator	Definition
HDI index	Composite measure of three life dimensions: life expectancy, per capita income and education
Health index	Life expectancy at birth in 2014; expressed as an index using a minimum value of 20 years and a maximum value of 85 years
Life expectancy at birth	Number of years a new-born infant could expect to live if prevailing patterns of age-specific mortality rates at the time of birth stay the same throughout the infant's life
Education index	Education index is an average of mean years of schooling (of adults) and expected years of schooling (of children) in 2014, both expressed as an index obtained by scaling with the corresponding maxima
GDP	Gross domestic product (GDP) per capita in 2013
Life expectancy - M	Life expectancy at birth, male (years)
Life expectancy - F	Life expectancy at birth, female (years)
Water (UN)	Accessibility of drinkable tap water
Sanitation (UN)	Accessibility of advanced sanitation

Development indicator and short descriptions are given.

Supplementary Table 13. Development level of 14 countries expressed through 45 indicators.

	China	Thailand	Sweden	Germany	England	Scotland	Croatia	Italy	Kosovo	Russia	Turkey	Uganda	Trinidad	Papua
**SDG index**	60	56	85	80	82	82	70	78	65	54	58	31	67	36
**Disaster**	39	29	100	100	100	100	100	61	40	57	33	53	100	32
**Stunting**	87	85	100	100	100	100	91	100	91	85	86	55	95	51
**Wasting**	91	84	100	100	100	100	88	100	87	90	96	82	87	86
**Overweight**	62	71	51	47	64	64	46	39	65	46	65	82	81	71
**MMR**	62	61	79	71	70	70	71	79	68	61	64	28	46	22
**SBA**	97	99	100	100	99	99	99	99	99	99	94	68	100	65
**Under-5 mort**	66	81	94	89	84	84	85	91	75	72	61	32	58	42
**NN mort**	69	80	94	91	85	85	84	90	76	75	59	34	51	47
**HIV**	46	34	65	57	51	51	69	54	57	32	64	15	35	35
**Tuberculosis**	45	45	82	86	74	74	69	87	70	41	69	29	62	48
**Malaria**	94	26	100	100	100	100	100	100	100	100	100	5	100	6
**Hepatitis B**	40	33	85	85	85	85	66	82	66	49	43	33	67	21
**NTDs**	89	87	100	100	100	100	100	100	100	99	97	85	98	54
**NCDs**	58	61	87	77	78	78	62	85	54	41	73	46	47	19
**Suicide**	59	42	51	55	64	64	47	71	44	21	81	43	46	42
**Alcohol**	74	74	57	54	57	57	54	68	59	7	90	58	65	87
**Road injuries**	49	36	94	88	94	94	72	75	69	53	66	35	55	37
**FP need met**	86	66	91	92	95	95	34	86	37	75	70	41	76	47
**Adol birth rate**	69	54	84	86	73	73	80	87	70	67	61	24	60	44
**UHC Tracer**	81	69	99	96	100	100	84	97	84	85	82	69	93	45
**Air poll mort**	48	61	96	83	83	83	68	83	58	62	71	33	74	28
**WaSH mort**	75	49	87	84	77	77	88	96	88	79	76	22	64	21
**Poisons**	50	69	71	94	75	75	84	81	73	47	80	37	71	44
**Smoking**	52	60	75	46	55	55	32	52	43	41	47	87	68	36
**IPV**	96	57	80	70	80	80	91	66	82	64	53	18	73	69
**Water**	500	34	100	100	100	100	81	100	74	86	29	26	47	14
**Sanitation**	66	98	100	100	100	100	97	100	85	79	87	20	90	20
**Hygiene**	38	46	95	89	93	93	43	86	43	69	28	3	53	16
**HH air poll**	79	88	100	100	100	100	94	100	78	98	98	2	100	55
**Occ risk burden**	43	54	81	71	74	74	63	72	60	67	57	31	73	9
**Mean PM 2.5**	25	46	81	62	65	65	51	53	51	58	37	24	62	62
**Violence**	69	34	74	81	86	86	72	77	55	25	58	39	19	36
**War**	100	100	100	100	100	100	100	100	100	31	19	100	100	100
**MDG index**	70	63	94	92	90	90	80	92	76	75	67	33	69	33
**Non-MDG index**	55	54	80	73	78	78	64	70	60	46	54	29	67	37
**HDI index**	73	73	91	92	91	91	82	87	77	80	76	48	77	51
**Life exp**	76	74	82	81	81	81	77	83	75	70	75	59	70	63
**Life exp F**	78	78	84	83	83	83	81	86	78	76	79	61	74	65
**Life exp M**	75	71	81	79	79	79	74	81	72	65	72	57	67	61
**GDP**	11525	13932	43741	43207	37017	37017	20063	34167	12893	23564	18660	1368	29469	2458

**Education index**	61	62	84	89	89	89	78	78	75	81	66	45	71	41
**Health Index**	86	84	96	94	93	93	88	97	85	77	85	59	78	66
**Water (UN)**	96	98	100	100	100	100	100	100	99	97	100	79	95	40
**Sanitation (UN)**	77	93	99	99	99	99	97	100	96	72	95	19	92	19

Higher indicator value suggests better conditions impacting on human well-being in a given country. Indicator descriptions are given in Supplementary Tables 11 and 12.

*MMR = maternal mortality ratio; SBA = skilled birth attendance; Under-5 mort = Under-5 mortality; NN mort = neonatal mortality; NTDs = neglected tropical diseases; NCDs = non-communicable diseases; FP need met = family planning need met, modern contraception; Adol birth rate = adolescent birth rate; UHC = universal health coverage; Air poll mort = air pollution mortality; WaSH Mort = water, sanitation, and hygiene mortality; IPV = intimate partner violence; HH air poll = household air pollution; MDG index = Millennium Development Goals Index; non-MDG index = health-related Sustainable Development Goals not included in MDG; HDI = Human Development Index; Life exp = Life expectancy at birth; Life exp F = Life expectancy – F; Life exp M = Life expectancy – M.
