# Supplementary material for: Global variability of the human IgG glycome
Source: Aging (Albany NY). 2020 Aug 12;12(15):15222–59. doi: 10.18632/aging.103884 (PMC7467356; doi:10.18632/aging.103884)
Supplement: Supplementary Table 14 [file aging-12-103884-s003..docx]

Supplementary Table 14. Correlation between used development indicators.

|  | HDI | SDG | Disaster | Stunting | Wasting | Overweight | MMR | SBA | Under5Mort | Nnmort | HIV | Tuberculosis | Malaria | HepatB | NTDs | NCDs | Suicide | Alcohol | RoadInjuries | FPneedMet | AdolBirthRate | UHCTracer | AirPollMort | WaSHMort | Poisons | Smoking | IPV | Water | Sanitation | Hygiene | HHAirPoll | OccRiskBurden | MeanPM25 | Violece | War | MDG | Non.MDG | LifeExp | LifeExp_F | LifeExp_M | GDP_2013 | EduIndx_2014 | HealthIndx_2014 | Water_UN | Sanitation_UN |
| --- | --- | --- | --- | --- | --- | --- | --- | --- | --- | --- | --- | --- | --- | --- | --- | --- | --- | --- | --- | --- | --- | --- | --- | --- | --- | --- | --- | --- | --- | --- | --- | --- | --- | --- | --- | --- | --- | --- | --- | --- | --- | --- | --- | --- | --- |
| HDI | 1,00 | 0,96 | 0,65 | 0,97 | 0,80 | -0,63 | 0,92 | 0,89 | 0,92 | 0,88 | 0,69 | 0,80 | 0,18 | 0,88 | 0,05 | 0,80 | 0,35 | -0,32 | 0,87 | 0,67 | 0,91 | 0,90 | 0,94 | 0,89 | 0,78 | -0,16 | 0,56 | 0,42 | 0,50 | 0,91 | 0,87 | 0,93 | 0,56 | 0,64 | 0,00 | 0,99 | 0,93 | 0,93 | 0,96 | 0,89 | 0,91 | 0,97 | 0,93 | 0,78 | 0,91 |
| SDG | 0,96 | 1,00 | 0,71 | 0,95 | 0,81 | -0,54 | 0,87 | 0,81 | 0,89 | 0,85 | 0,72 | 0,87 | 0,18 | 0,93 | 0,06 | 0,83 | 0,44 | -0,16 | 0,91 | 0,65 | 0,90 | 0,89 | 0,94 | 0,83 | 0,79 | -0,07 | 0,61 | 0,46 | 0,53 | 0,90 | 0,80 | 0,88 | 0,60 | 0,72 | 0,21 | 0,97 | 0,99 | 0,94 | 0,93 | 0,93 | 0,91 | 0,91 | 0,94 | 0,70 | 0,88 |
| Disaster | 0,65 | 0,71 | 1,00 | 0,60 | 0,52 | -0,27 | 0,40 | 0,42 | 0,49 | 0,45 | 0,35 | 0,57 | -0,13 | 0,81 | -0,21 | 0,48 | 0,10 | -0,37 | 0,73 | 0,42 | 0,52 | 0,72 | 0,69 | 0,42 | 0,50 | 0,15 | 0,35 | 0,36 | 0,49 | 0,68 | 0,42 | 0,68 | 0,62 | 0,46 | 0,32 | 0,62 | 0,73 | 0,48 | 0,47 | 0,48 | 0,78 | 0,71 | 0,48 | 0,39 | 0,45 |
| Stunting | 0,97 | 0,95 | 0,60 | 1,00 | 0,71 | -0,52 | 0,91 | 0,94 | 0,88 | 0,82 | 0,66 | 0,77 | 0,25 | 0,85 | 0,13 | 0,80 | 0,38 | -0,24 | 0,79 | 0,64 | 0,87 | 0,92 | 0,92 | 0,89 | 0,78 | -0,08 | 0,57 | 0,39 | 0,44 | 0,84 | 0,85 | 0,94 | 0,45 | 0,57 | 0,05 | 0,96 | 0,92 | 0,92 | 0,94 | 0,88 | 0,85 | 0,91 | 0,92 | 0,85 | 0,96 |
| Wasting | 0,80 | 0,81 | 0,52 | 0,71 | 1,00 | -0,58 | 0,71 | 0,50 | 0,68 | 0,69 | 0,63 | 0,81 | -0,06 | 0,77 | -0,18 | 0,81 | 0,64 | -0,06 | 0,88 | 0,80 | 0,75 | 0,75 | 0,81 | 0,66 | 0,60 | -0,07 | 0,35 | 0,50 | 0,54 | 0,83 | 0,64 | 0,65 | 0,50 | 0,79 | -0,06 | 0,80 | 0,78 | 0,82 | 0,78 | 0,83 | 0,85 | 0,75 | 0,82 | 0,45 | 0,60 |
| Overweight | -0,63 | -0,54 | -0,27 | -0,52 | -0,58 | 1,00 | -0,72 | -0,50 | -0,73 | -0,77 | -0,60 | -0,56 | -0,04 | -0,50 | 0,05 | -0,48 | -0,10 | 0,41 | -0,52 | -0,29 | -0,81 | -0,45 | -0,51 | -0,74 | -0,46 | 0,48 | -0,40 | 0,00 | 0,03 | -0,59 | -0,55 | -0,48 | -0,32 | -0,52 | 0,15 | -0,68 | -0,43 | -0,65 | -0,69 | -0,60 | -0,53 | -0,61 | -0,65 | -0,40 | -0,47 |
| MMR | 0,92 | 0,87 | 0,40 | 0,91 | 0,71 | -0,72 | 1,00 | 0,88 | 0,94 | 0,91 | 0,77 | 0,74 | 0,29 | 0,75 | 0,18 | 0,85 | 0,40 | -0,24 | 0,76 | 0,50 | 0,90 | 0,80 | 0,84 | 0,93 | 0,74 | -0,19 | 0,52 | 0,31 | 0,32 | 0,76 | 0,77 | 0,83 | 0,33 | 0,68 | -0,04 | 0,94 | 0,80 | 0,95 | 0,96 | 0,91 | 0,73 | 0,85 | 0,95 | 0,84 | 0,91 |
| SBA | 0,89 | 0,81 | 0,42 | 0,94 | 0,50 | -0,50 | 0,88 | 1,00 | 0,83 | 0,77 | 0,58 | 0,57 | 0,30 | 0,66 | 0,18 | 0,65 | 0,20 | -0,31 | 0,59 | 0,51 | 0,80 | 0,79 | 0,79 | 0,87 | 0,69 | -0,16 | 0,57 | 0,28 | 0,29 | 0,70 | 0,86 | 0,88 | 0,32 | 0,37 | -0,08 | 0,88 | 0,77 | 0,82 | 0,88 | 0,75 | 0,69 | 0,83 | 0,82 | 0,89 | 0,95 |
| Under5Mort | 0,92 | 0,89 | 0,49 | 0,88 | 0,68 | -0,73 | 0,94 | 0,83 | 1,00 | 0,99 | 0,70 | 0,75 | 0,19 | 0,77 | 0,09 | 0,77 | 0,24 | -0,27 | 0,76 | 0,52 | 0,91 | 0,72 | 0,84 | 0,83 | 0,75 | -0,23 | 0,57 | 0,29 | 0,36 | 0,85 | 0,79 | 0,80 | 0,54 | 0,64 | 0,14 | 0,93 | 0,85 | 0,94 | 0,95 | 0,90 | 0,78 | 0,86 | 0,94 | 0,70 | 0,86 |
| Nnmort | 0,88 | 0,85 | 0,45 | 0,82 | 0,69 | -0,77 | 0,91 | 0,77 | 0,99 | 1,00 | 0,68 | 0,71 | 0,18 | 0,73 | 0,09 | 0,74 | 0,21 | -0,30 | 0,75 | 0,51 | 0,90 | 0,67 | 0,78 | 0,80 | 0,68 | -0,27 | 0,58 | 0,33 | 0,37 | 0,84 | 0,74 | 0,73 | 0,53 | 0,68 | 0,15 | 0,90 | 0,80 | 0,91 | 0,92 | 0,88 | 0,75 | 0,84 | 0,92 | 0,63 | 0,79 |
| HIV | 0,69 | 0,72 | 0,35 | 0,66 | 0,63 | -0,60 | 0,77 | 0,58 | 0,70 | 0,68 | 1,00 | 0,82 | 0,32 | 0,59 | 0,21 | 0,66 | 0,54 | 0,14 | 0,74 | 0,20 | 0,81 | 0,51 | 0,65 | 0,78 | 0,78 | -0,43 | 0,62 | 0,14 | 0,17 | 0,45 | 0,66 | 0,52 | 0,35 | 0,70 | -0,05 | 0,71 | 0,69 | 0,79 | 0,78 | 0,79 | 0,53 | 0,61 | 0,79 | 0,48 | 0,73 |
| Tuberculosis | 0,80 | 0,87 | 0,57 | 0,77 | 0,81 | -0,56 | 0,74 | 0,57 | 0,75 | 0,71 | 0,82 | 1,00 | 0,26 | 0,86 | 0,15 | 0,76 | 0,58 | 0,08 | 0,88 | 0,45 | 0,83 | 0,71 | 0,83 | 0,74 | 0,88 | -0,20 | 0,46 | 0,18 | 0,34 | 0,73 | 0,68 | 0,69 | 0,59 | 0,71 | 0,16 | 0,81 | 0,86 | 0,84 | 0,82 | 0,85 | 0,80 | 0,74 | 0,84 | 0,46 | 0,74 |
| Malaria | 0,18 | 0,18 | -0,13 | 0,25 | -0,06 | -0,04 | 0,29 | 0,30 | 0,19 | 0,18 | 0,32 | 0,26 | 1,00 | 0,23 | 0,99 | 0,02 | -0,07 | -0,11 | 0,21 | -0,30 | 0,24 | 0,18 | 0,05 | 0,41 | 0,22 | -0,25 | 0,31 | -0,05 | -0,03 | 0,02 | 0,10 | 0,19 | 0,04 | 0,07 | 0,06 | 0,23 | 0,16 | 0,17 | 0,18 | 0,15 | -0,05 | 0,23 | 0,17 | 0,27 | 0,31 |
| HepatB | 0,88 | 0,93 | 0,81 | 0,85 | 0,77 | -0,50 | 0,75 | 0,66 | 0,77 | 0,73 | 0,59 | 0,86 | 0,23 | 1,00 | 0,13 | 0,76 | 0,35 | -0,31 | 0,93 | 0,55 | 0,80 | 0,92 | 0,88 | 0,76 | 0,72 | 0,04 | 0,44 | 0,40 | 0,53 | 0,88 | 0,62 | 0,86 | 0,62 | 0,68 | 0,26 | 0,90 | 0,91 | 0,80 | 0,79 | 0,80 | 0,91 | 0,91 | 0,80 | 0,63 | 0,73 |
| NTDs | 0,05 | 0,06 | -0,21 | 0,13 | -0,18 | 0,05 | 0,18 | 0,18 | 0,09 | 0,09 | 0,21 | 0,15 | 0,99 | 0,13 | 1,00 | -0,06 | -0,13 | -0,07 | 0,10 | -0,40 | 0,11 | 0,06 | -0,06 | 0,28 | 0,13 | -0,19 | 0,20 | -0,11 | -0,08 | -0,08 | -0,03 | 0,08 | -0,02 | 0,00 | 0,10 | 0,10 | 0,05 | 0,06 | 0,06 | 0,04 | -0,16 | 0,12 | 0,06 | 0,18 | 0,20 |
| NCDs | 0,80 | 0,83 | 0,48 | 0,80 | 0,81 | -0,48 | 0,85 | 0,65 | 0,77 | 0,74 | 0,66 | 0,76 | 0,02 | 0,76 | -0,06 | 1,00 | 0,67 | 0,02 | 0,80 | 0,62 | 0,70 | 0,81 | 0,86 | 0,70 | 0,73 | 0,19 | 0,22 | 0,37 | 0,43 | 0,72 | 0,55 | 0,76 | 0,25 | 0,79 | 0,08 | 0,82 | 0,80 | 0,87 | 0,84 | 0,90 | 0,77 | 0,72 | 0,87 | 0,75 | 0,77 |
| Suicide | 0,35 | 0,44 | 0,10 | 0,38 | 0,64 | -0,10 | 0,40 | 0,20 | 0,24 | 0,21 | 0,54 | 0,58 | -0,07 | 0,35 | -0,13 | 0,67 | 1,00 | 0,60 | 0,48 | 0,42 | 0,33 | 0,39 | 0,42 | 0,34 | 0,53 | 0,02 | 0,11 | 0,38 | 0,36 | 0,22 | 0,29 | 0,23 | -0,10 | 0,66 | -0,04 | 0,35 | 0,45 | 0,54 | 0,47 | 0,60 | 0,33 | 0,21 | 0,54 | 0,29 | 0,41 |
| Alcohol | -0,32 | -0,16 | -0,37 | -0,24 | -0,06 | 0,41 | -0,24 | -0,31 | -0,27 | -0,30 | 0,14 | 0,08 | -0,11 | -0,31 | -0,07 | 0,02 | 0,60 | 1,00 | -0,19 | -0,11 | -0,23 | -0,37 | -0,21 | -0,31 | 0,11 | 0,04 | -0,04 | -0,07 | -0,12 | -0,40 | -0,15 | -0,43 | -0,27 | 0,09 | 0,21 | -0,33 | -0,08 | -0,05 | -0,13 | 0,04 | -0,30 | -0,50 | -0,05 | -0,33 | -0,11 |
| RoadInjuries | 0,87 | 0,91 | 0,73 | 0,79 | 0,88 | -0,52 | 0,76 | 0,59 | 0,76 | 0,75 | 0,74 | 0,88 | 0,21 | 0,93 | 0,10 | 0,80 | 0,48 | -0,19 | 1,00 | 0,56 | 0,79 | 0,84 | 0,86 | 0,74 | 0,72 | -0,08 | 0,48 | 0,49 | 0,62 | 0,83 | 0,63 | 0,77 | 0,62 | 0,81 | 0,11 | 0,87 | 0,89 | 0,83 | 0,80 | 0,84 | 0,87 | 0,88 | 0,83 | 0,56 | 0,71 |
| FPneedMet | 0,67 | 0,65 | 0,42 | 0,64 | 0,80 | -0,29 | 0,50 | 0,51 | 0,52 | 0,51 | 0,20 | 0,45 | -0,30 | 0,55 | -0,40 | 0,62 | 0,42 | -0,11 | 0,56 | 1,00 | 0,52 | 0,66 | 0,68 | 0,42 | 0,31 | 0,20 | 0,27 | 0,57 | 0,50 | 0,78 | 0,59 | 0,58 | 0,39 | 0,45 | -0,03 | 0,64 | 0,65 | 0,63 | 0,61 | 0,64 | 0,77 | 0,59 | 0,63 | 0,42 | 0,49 |
| AdolBirthRate | 0,91 | 0,90 | 0,52 | 0,87 | 0,75 | -0,81 | 0,90 | 0,80 | 0,91 | 0,90 | 0,81 | 0,83 | 0,24 | 0,80 | 0,11 | 0,70 | 0,33 | -0,23 | 0,79 | 0,52 | 1,00 | 0,75 | 0,81 | 0,92 | 0,75 | -0,38 | 0,71 | 0,27 | 0,25 | 0,80 | 0,83 | 0,76 | 0,52 | 0,67 | 0,07 | 0,94 | 0,84 | 0,92 | 0,94 | 0,89 | 0,79 | 0,85 | 0,92 | 0,62 | 0,83 |
| UHCTracer | 0,90 | 0,89 | 0,72 | 0,92 | 0,75 | -0,45 | 0,80 | 0,79 | 0,72 | 0,67 | 0,51 | 0,71 | 0,18 | 0,92 | 0,06 | 0,81 | 0,39 | -0,37 | 0,84 | 0,66 | 0,75 | 1,00 | 0,90 | 0,81 | 0,64 | 0,16 | 0,37 | 0,45 | 0,50 | 0,83 | 0,65 | 0,94 | 0,41 | 0,59 | 0,03 | 0,91 | 0,86 | 0,79 | 0,80 | 0,77 | 0,88 | 0,91 | 0,79 | 0,83 | 0,80 |
| AirPollMort | 0,94 | 0,94 | 0,69 | 0,92 | 0,81 | -0,51 | 0,84 | 0,79 | 0,84 | 0,78 | 0,65 | 0,83 | 0,05 | 0,88 | -0,06 | 0,86 | 0,42 | -0,21 | 0,86 | 0,68 | 0,81 | 0,90 | 1,00 | 0,78 | 0,78 | 0,07 | 0,37 | 0,28 | 0,45 | 0,88 | 0,80 | 0,94 | 0,62 | 0,57 | -0,01 | 0,92 | 0,92 | 0,87 | 0,89 | 0,84 | 0,95 | 0,89 | 0,87 | 0,74 | 0,87 |
| WaSHMort | 0,89 | 0,83 | 0,42 | 0,89 | 0,66 | -0,74 | 0,93 | 0,87 | 0,83 | 0,80 | 0,78 | 0,74 | 0,41 | 0,76 | 0,28 | 0,70 | 0,34 | -0,31 | 0,74 | 0,42 | 0,92 | 0,81 | 0,78 | 1,00 | 0,71 | -0,32 | 0,61 | 0,24 | 0,22 | 0,69 | 0,79 | 0,82 | 0,32 | 0,58 | -0,13 | 0,92 | 0,76 | 0,86 | 0,90 | 0,81 | 0,69 | 0,85 | 0,87 | 0,80 | 0,87 |
| Poisons | 0,78 | 0,79 | 0,50 | 0,78 | 0,60 | -0,46 | 0,74 | 0,69 | 0,75 | 0,68 | 0,78 | 0,88 | 0,22 | 0,72 | 0,13 | 0,73 | 0,53 | 0,11 | 0,72 | 0,31 | 0,75 | 0,64 | 0,78 | 0,71 | 1,00 | -0,27 | 0,38 | 0,08 | 0,27 | 0,58 | 0,72 | 0,70 | 0,39 | 0,58 | 0,08 | 0,75 | 0,79 | 0,80 | 0,81 | 0,78 | 0,69 | 0,71 | 0,80 | 0,62 | 0,85 |
| Smoking | -0,16 | -0,07 | 0,15 | -0,08 | -0,07 | 0,48 | -0,19 | -0,16 | -0,23 | -0,27 | -0,43 | -0,20 | -0,25 | 0,04 | -0,19 | 0,19 | 0,02 | 0,04 | -0,08 | 0,20 | -0,38 | 0,16 | 0,07 | -0,32 | -0,27 | 1,00 | -0,52 | 0,01 | 0,02 | -0,01 | -0,40 | 0,09 | -0,10 | -0,13 | 0,26 | -0,15 | -0,02 | -0,20 | -0,24 | -0,13 | 0,07 | -0,15 | -0,20 | 0,10 | -0,17 |
| IPV | 0,56 | 0,61 | 0,35 | 0,57 | 0,35 | -0,40 | 0,52 | 0,57 | 0,57 | 0,58 | 0,62 | 0,46 | 0,31 | 0,44 | 0,20 | 0,22 | 0,11 | -0,04 | 0,48 | 0,27 | 0,71 | 0,37 | 0,37 | 0,61 | 0,38 | -0,52 | 1,00 | 0,45 | 0,27 | 0,45 | 0,65 | 0,35 | 0,40 | 0,45 | 0,26 | 0,58 | 0,62 | 0,61 | 0,59 | 0,60 | 0,37 | 0,50 | 0,61 | 0,26 | 0,55 |
| Water | 0,42 | 0,46 | 0,36 | 0,39 | 0,50 | 0,00 | 0,31 | 0,28 | 0,29 | 0,33 | 0,14 | 0,18 | -0,05 | 0,40 | -0,11 | 0,37 | 0,38 | -0,07 | 0,49 | 0,57 | 0,27 | 0,45 | 0,28 | 0,24 | 0,08 | 0,01 | 0,45 | 1,00 | 0,87 | 0,47 | 0,25 | 0,27 | 0,11 | 0,60 | 0,22 | 0,41 | 0,48 | 0,42 | 0,36 | 0,47 | 0,35 | 0,42 | 0,42 | 0,26 | 0,28 |
| Sanitation | 0,50 | 0,53 | 0,49 | 0,44 | 0,54 | 0,03 | 0,32 | 0,29 | 0,36 | 0,37 | 0,17 | 0,34 | -0,03 | 0,53 | -0,08 | 0,43 | 0,36 | -0,12 | 0,62 | 0,50 | 0,25 | 0,50 | 0,45 | 0,22 | 0,27 | 0,02 | 0,27 | 0,87 | 1,00 | 0,57 | 0,33 | 0,42 | 0,36 | 0,55 | 0,16 | 0,46 | 0,57 | 0,44 | 0,40 | 0,46 | 0,48 | 0,54 | 0,44 | 0,28 | 0,36 |
| Hygiene | 0,91 | 0,90 | 0,68 | 0,84 | 0,83 | -0,59 | 0,76 | 0,70 | 0,85 | 0,84 | 0,45 | 0,73 | 0,02 | 0,88 | -0,08 | 0,72 | 0,22 | -0,40 | 0,83 | 0,78 | 0,80 | 0,83 | 0,88 | 0,69 | 0,58 | -0,01 | 0,45 | 0,47 | 0,57 | 1,00 | 0,74 | 0,83 | 0,72 | 0,59 | 0,14 | 0,90 | 0,87 | 0,83 | 0,83 | 0,80 | 0,94 | 0,90 | 0,82 | 0,57 | 0,70 |
| HHAirPoll | 0,87 | 0,80 | 0,42 | 0,85 | 0,64 | -0,55 | 0,77 | 0,86 | 0,79 | 0,74 | 0,66 | 0,68 | 0,10 | 0,62 | -0,03 | 0,55 | 0,29 | -0,15 | 0,63 | 0,59 | 0,83 | 0,65 | 0,80 | 0,79 | 0,72 | -0,40 | 0,65 | 0,25 | 0,33 | 0,74 | 1,00 | 0,77 | 0,57 | 0,36 | -0,20 | 0,83 | 0,79 | 0,82 | 0,87 | 0,74 | 0,74 | 0,78 | 0,82 | 0,60 | 0,87 |
| OccRiskBurden | 0,93 | 0,88 | 0,68 | 0,94 | 0,65 | -0,48 | 0,83 | 0,88 | 0,80 | 0,73 | 0,52 | 0,69 | 0,19 | 0,86 | 0,08 | 0,76 | 0,23 | -0,43 | 0,77 | 0,58 | 0,76 | 0,94 | 0,94 | 0,82 | 0,70 | 0,09 | 0,35 | 0,27 | 0,42 | 0,83 | 0,77 | 1,00 | 0,52 | 0,42 | -0,05 | 0,91 | 0,85 | 0,79 | 0,83 | 0,73 | 0,88 | 0,92 | 0,79 | 0,86 | 0,88 |
| MeanPM25 | 0,56 | 0,60 | 0,62 | 0,45 | 0,50 | -0,32 | 0,33 | 0,32 | 0,54 | 0,53 | 0,35 | 0,59 | 0,04 | 0,62 | -0,02 | 0,25 | -0,10 | -0,27 | 0,62 | 0,39 | 0,52 | 0,41 | 0,62 | 0,32 | 0,39 | -0,10 | 0,40 | 0,11 | 0,36 | 0,72 | 0,57 | 0,52 | 1,00 | 0,21 | 0,17 | 0,53 | 0,64 | 0,44 | 0,45 | 0,41 | 0,71 | 0,59 | 0,44 | 0,05 | 0,37 |
| Violece | 0,64 | 0,72 | 0,46 | 0,57 | 0,79 | -0,52 | 0,68 | 0,37 | 0,64 | 0,68 | 0,70 | 0,71 | 0,07 | 0,68 | 0,00 | 0,79 | 0,66 | 0,09 | 0,81 | 0,45 | 0,67 | 0,59 | 0,57 | 0,58 | 0,58 | -0,13 | 0,45 | 0,60 | 0,55 | 0,59 | 0,36 | 0,42 | 0,21 | 1,00 | 0,27 | 0,68 | 0,69 | 0,77 | 0,69 | 0,83 | 0,57 | 0,60 | 0,77 | 0,41 | 0,51 |
| War | 0,00 | 0,21 | 0,32 | 0,05 | -0,06 | 0,15 | -0,04 | -0,08 | 0,14 | 0,15 | -0,05 | 0,16 | 0,06 | 0,26 | 0,10 | 0,08 | -0,04 | 0,21 | 0,11 | -0,03 | 0,07 | 0,03 | -0,01 | -0,13 | 0,08 | 0,26 | 0,26 | 0,22 | 0,16 | 0,14 | -0,20 | -0,05 | 0,17 | 0,27 | 1,00 | 0,05 | 0,27 | 0,11 | 0,02 | 0,20 | 0,08 | -0,02 | 0,11 | -0,14 | -0,03 |
| MDG | 0,99 | 0,97 | 0,62 | 0,96 | 0,80 | -0,68 | 0,94 | 0,88 | 0,93 | 0,90 | 0,71 | 0,81 | 0,23 | 0,90 | 0,10 | 0,82 | 0,35 | -0,33 | 0,87 | 0,64 | 0,94 | 0,91 | 0,92 | 0,92 | 0,75 | -0,15 | 0,58 | 0,41 | 0,46 | 0,90 | 0,83 | 0,91 | 0,53 | 0,68 | 0,05 | 1,00 | 0,92 | 0,95 | 0,96 | 0,91 | 0,89 | 0,96 | 0,95 | 0,78 | 0,90 |
| Non.MDG | 0,93 | 0,99 | 0,73 | 0,92 | 0,78 | -0,43 | 0,80 | 0,77 | 0,85 | 0,80 | 0,69 | 0,86 | 0,16 | 0,91 | 0,05 | 0,80 | 0,45 | -0,08 | 0,89 | 0,65 | 0,84 | 0,86 | 0,92 | 0,76 | 0,79 | -0,02 | 0,62 | 0,48 | 0,57 | 0,87 | 0,79 | 0,85 | 0,64 | 0,69 | 0,27 | 0,92 | 1,00 | 0,91 | 0,89 | 0,91 | 0,90 | 0,87 | 0,91 | 0,64 | 0,86 |
| LifeExp | 0,93 | 0,94 | 0,48 | 0,92 | 0,82 | -0,65 | 0,95 | 0,82 | 0,94 | 0,91 | 0,79 | 0,84 | 0,17 | 0,80 | 0,06 | 0,87 | 0,54 | -0,05 | 0,83 | 0,63 | 0,92 | 0,79 | 0,87 | 0,86 | 0,80 | -0,20 | 0,61 | 0,42 | 0,44 | 0,83 | 0,82 | 0,79 | 0,44 | 0,77 | 0,11 | 0,95 | 0,91 | 1,00 | 0,99 | 0,99 | 0,81 | 0,84 | 1,00 | 0,72 | 0,90 |
| LifeExp_F | 0,96 | 0,93 | 0,47 | 0,94 | 0,78 | -0,69 | 0,96 | 0,88 | 0,95 | 0,92 | 0,78 | 0,82 | 0,18 | 0,79 | 0,06 | 0,84 | 0,47 | -0,13 | 0,80 | 0,61 | 0,94 | 0,80 | 0,89 | 0,90 | 0,81 | -0,24 | 0,59 | 0,36 | 0,40 | 0,83 | 0,87 | 0,83 | 0,45 | 0,69 | 0,02 | 0,96 | 0,89 | 0,99 | 1,00 | 0,96 | 0,81 | 0,87 | 0,99 | 0,76 | 0,93 |
| LifeExp_M | 0,89 | 0,93 | 0,48 | 0,88 | 0,83 | -0,60 | 0,91 | 0,75 | 0,90 | 0,88 | 0,79 | 0,85 | 0,15 | 0,80 | 0,04 | 0,90 | 0,60 | 0,04 | 0,84 | 0,64 | 0,89 | 0,77 | 0,84 | 0,81 | 0,78 | -0,13 | 0,60 | 0,47 | 0,46 | 0,80 | 0,74 | 0,73 | 0,41 | 0,83 | 0,20 | 0,91 | 0,91 | 0,99 | 0,96 | 1,00 | 0,79 | 0,79 | 0,99 | 0,67 | 0,85 |
| GDP_2013 | 0,91 | 0,91 | 0,78 | 0,85 | 0,85 | -0,53 | 0,73 | 0,69 | 0,78 | 0,75 | 0,53 | 0,80 | -0,05 | 0,91 | -0,16 | 0,77 | 0,33 | -0,30 | 0,87 | 0,77 | 0,79 | 0,88 | 0,95 | 0,69 | 0,69 | 0,07 | 0,37 | 0,35 | 0,48 | 0,94 | 0,74 | 0,88 | 0,71 | 0,57 | 0,08 | 0,89 | 0,90 | 0,81 | 0,81 | 0,79 | 1,00 | 0,89 | 0,81 | 0,59 | 0,73 |
| EduIndx_2014 | 0,97 | 0,91 | 0,71 | 0,91 | 0,75 | -0,61 | 0,85 | 0,83 | 0,86 | 0,84 | 0,61 | 0,74 | 0,23 | 0,91 | 0,12 | 0,72 | 0,21 | -0,50 | 0,88 | 0,59 | 0,85 | 0,91 | 0,89 | 0,85 | 0,71 | -0,15 | 0,50 | 0,42 | 0,54 | 0,90 | 0,78 | 0,92 | 0,59 | 0,60 | -0,02 | 0,96 | 0,87 | 0,84 | 0,87 | 0,79 | 0,89 | 1,00 | 0,84 | 0,76 | 0,84 |
| HealthIndx_2014 | 0,93 | 0,94 | 0,48 | 0,92 | 0,82 | -0,65 | 0,95 | 0,82 | 0,94 | 0,92 | 0,79 | 0,84 | 0,17 | 0,80 | 0,06 | 0,87 | 0,54 | -0,05 | 0,83 | 0,63 | 0,92 | 0,79 | 0,87 | 0,87 | 0,80 | -0,20 | 0,61 | 0,42 | 0,44 | 0,82 | 0,82 | 0,79 | 0,44 | 0,77 | 0,11 | 0,95 | 0,91 | 1,00 | 0,99 | 0,99 | 0,81 | 0,84 | 1,00 | 0,72 | 0,90 |
| Water_UN | 0,78 | 0,70 | 0,39 | 0,85 | 0,45 | -0,40 | 0,84 | 0,89 | 0,70 | 0,63 | 0,48 | 0,46 | 0,27 | 0,63 | 0,18 | 0,75 | 0,29 | -0,33 | 0,56 | 0,42 | 0,62 | 0,83 | 0,74 | 0,80 | 0,62 | 0,10 | 0,26 | 0,26 | 0,28 | 0,57 | 0,60 | 0,86 | 0,05 | 0,41 | -0,14 | 0,78 | 0,64 | 0,72 | 0,76 | 0,67 | 0,59 | 0,76 | 0,72 | 1,00 | 0,86 |
| Sanitation_UN | 0,91 | 0,88 | 0,45 | 0,96 | 0,60 | -0,47 | 0,91 | 0,95 | 0,86 | 0,79 | 0,73 | 0,74 | 0,31 | 0,73 | 0,20 | 0,77 | 0,41 | -0,11 | 0,71 | 0,49 | 0,83 | 0,80 | 0,87 | 0,87 | 0,85 | -0,17 | 0,55 | 0,28 | 0,36 | 0,70 | 0,87 | 0,88 | 0,37 | 0,51 | -0,03 | 0,90 | 0,86 | 0,90 | 0,93 | 0,85 | 0,73 | 0,84 | 0,90 | 0,86 | 1,00 |
