# Supplementary material for: Global variability of the human IgG glycome
Source: Aging (Albany NY). 2020 Aug 12;12(15):15222–59. doi: 10.18632/aging.103884 (PMC7467356; doi:10.18632/aging.103884)
Supplement: Supplementary Tables 15-18 [file aging-12-103884-s006..pdf]

SUPPLEMENTARY TABLES

Supplementary Table 15. Description of directly measured subclass-specific Fc IgG glycan traits measured by LC-MS with mass list.

Glycan trait[1]		IgG1 glycopeptide m/z[3]		IgG2&3 glycopeptide m/z[4]		IgG4glycopeptide m/z[5]		Glycan trait description	Glycan trait calculation[6]
		$[M+2H]^{2+}$	$[M+3H]^{3+}$	$[M+2H]^{2+}$	$[M+3H]^{3+}$	$[M+2H]^{2+}$	$[M+3H]^{3+}$		
G0F	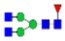	1317,527	878,687	1301,532	868,024	1309,529	873,356	Fraction of FA2 glycan in total subclass Fc glycans	*G0F/total subclass Fc glycans*100*
G1F	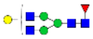	1398,553	932,705	1382,558	922,042	1390,556	927,373	Fraction of FA2G1 glycan in total subclass Fc glycans	*G1F/total subclass Fc glycans*100*
G2F	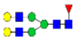	1479,58	986,722	1463,585	976,059	1471,582	981,391	Fraction of FA2G2 glycan in total subclass Fc glycans	*G2F/total subclass Fc glycans*100*
G0FN	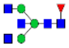	1419,067	946,38	1403,072	935,717	1411,069	941,049	Fraction of FA2B glycan in total subclass Fc glycans	*G0FN/total subclass Fc glycans*100*
G1FN	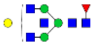	1500,093	1000,398	1484,098	989,735	1492,096	995,066	Fraction of FA2BG1 glycan in total subclass Fc glycans	*G1FN/total subclass Fc glycans*100*
G2FN	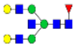	1581,119	1054,416	1565,125	1043,752	1573,122	1049,084	Fraction of FA2BG2 glycan in total subclass Fc glycans	*G2FN/total subclass Fc glycans*100*
G1FS1	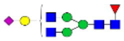	1544,101	1029,737	1528,106	1019,073	1536,104	1024,405	Fraction of FA2G1S1 glycan in total subclass Fc glycans	*G1FS1/total subclass Fc glycans*100*
G2FS1	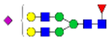	1625,127	1083,754	1609,133	1073,091	1617,13	1078,423	Fraction of FA2G2S1 glycan in total subclass Fc glycans	*G2FS1/total subclass Fc glycans*100*
G1FNS1	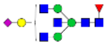	1645,641	1097,430	1629,646	1086,767	1637,643	1092,098	Fraction of FA2BG1S1 glycan in total subclass Fc glycans	*G1FNS1/total subclass Fc glycans*100*
G2FNS1	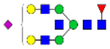	1726,667	1151,447	1710,672	1140,784	1718,67	1146,116	Fraction of FA2BG2S1 glycan in total subclass Fc glycans	*G2FNS1/total subclass Fc glycans*100*
G0	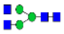	1244,498	830,001	1228,503	819,338	1236,501	824,67	Fraction of A2 glycan in total subclass Fc glycans	*G0/total subclass Fc glycans*100*
G1	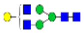	1325,524	884,019	1309,529	873,356	1317,527	878,687	Fraction of A2G1 glycan in total subclass Fc glycans	*G1/total subclass Fc glycans*100*
G2	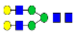	1406,551	938,036	1390,556	927,373	1398,553	932,705	Fraction of A2G2 glycan in total subclass Fc glycans	*G2/total subclass Fc glycans*100*
G0N	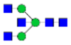	1346,038	897,694	1330,043	887,031	1338,04	892,363	Fraction of A2B glycan in total subclass Fc glycans	*G0N/total subclass Fc glycans*100*
G1N	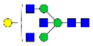	1427,064	951,712	1411,069	941,049	1419,067	946,38	Fraction of A2BG1 glycan in total subclass Fc glycans	*G1N/total subclass Fc glycans*100*
G2N	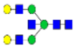	1508,090	1005,730	1492,096	995,066	1500,093	1000,398	Fraction of A2BG2 glycan in total subclass Fc glycans	*G2N/total subclass Fc glycans*100*
G1S1	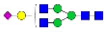	1471,072	981,051	1455,077	970,387	1463,075	975,719	Fraction of A2G1S1 glycan in total subclass Fc glycans	*G1S1/total subclass Fc glycans*100*
G2S1	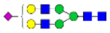	1552,098	1035,068	1536,104	1024,405	1544,101	1029,737	Fraction of A2G2S1 glycan in total subclass Fc glycans	*G2S1/total subclass Fc glycans*100*
G1NS1	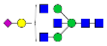	1572,612	1048,744	1556,617	1038,081	1564,614	1043,412	Fraction of A2BG1S1 glycan in total subclass Fc glycans	*G1NS1/total subclass Fc glycans*100*
G2NS1	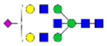	1653,638	1102,761	1637,643	1092,098	1645,641	1097,43	Fraction of A2BG2S1 glycan in total subclass Fc glycans	*G2NS1/total subclass Fc glycans*100*

[1]Glycan composition: N (N-acetylglucosamine), F (fucose), G (galactose) and S (N-acetylneuraminic acid) followed by a number representing the number and type of monosaccharides attached to A2 glycan.
[2]Glycan structures are drawn in GlycoWorkbench version 2. blue square = N-acetylglucosamine, red triangle = fucose, green circle = mannose, yellow circle = galactose, purple diamond = N-acetylneuraminic acid.
[3]IgG1 tryptic peptide sequence carrying glycan: E293EQYNSTYR301
[4]IgG4 tryptic peptide sequence carrying glycan: E293EQFNSTFR301
[5]IgG2&3 tryptic peptide sequence carrying glycan: E293EQFNSTYR301
[6]total subclass Fc glycans = sum of all 20 glycopeptides in one IgG subclass

Supplementary Table 16. Description of derived subclass-specific Fc IgG glycan traits measured by LC-MS with mass list.

Derived glycan trait	Derived trait description	Derived trait calculation
Core fucosylation	Fraction of structures containing core fucose in subclass specific Fc glycans	G0F+G1F+G2F+G0FN+G1FN+G2FN+G1FS1+G2FS1+G1FNS1+G2FNS1
Bisecting GlcNAc	Fraction of structures containing bisecting GlcNAc in subclass specific Fc glycans	G0FN+G1FN+G2FN+G1FNS1+G2FNS1+G0N+G1N+G2N+G1NS1+G2NS1
Agalactosylation	Fraction of agalactosylated structures in subclass specific Fc glycans	G0+G0F+G0N+G0FN
Monogalactosylation	Fraction of structures containing one galactose in subclass specific Fc glycans	G1F+G1FN+G1FS1+G1FNS1+G1+G1N+G1S1+G1NS1
Digalactosylation	Fraction of structures containing two galactoses in subclass specific Fc glycans	G2F+G2FN+G2FS1+G2FNS1+G2+G2N+G2S1+G2NS1
Sialylation	Fraction of structures containing sialic acid in subclass specific Fc glycans	G1FS1+G2FS1+G1FNS1+G2FNS1+G1S1+G2S1+G1NS1+G2NS1

Supplementary Table 17. Correlations between derived glycan traits measured by HILIC-UPLC.

	Agalactosylation	Monogalactosylation	Digalactosylation	Bisecting GlcNAc	Sialylation	Core fucosylation
Agalactosylation	1,00	-0,23	-0,89	0,38	-0,70	0,17
Monogalactosylation	-0,23	1,00	0,21	-0,07	-0,41	0,56
Digalactosylation	-0,89	0,21	1,00	-0,39	0,43	0,04
Bisecting GlcNAc	0,38	-0,07	-0,39	1,00	-0,23	-0,09
Sialylation	-0,70	-0,41	0,43	-0,23	1,00	-0,63
Core fucosylation	0,17	0,56	0,04	-0,09	-0,63	1,00

Supplementary Table 18. Correlations between derived subclass-specific Fc IgG glycan traits measured by LC-MS.

	IgG1_Agalactosylation	IgG1_Monogalactosylation	IgG1_Digalactosylation	IgG1_Sialylation	IgG1_Bisecting	IgG2_Agalactosylation	IgG2_Monogalactosylation	IgG2_Digalactosylation	IgG2_Sialylation	IgG2_Bisecting	IgG4_Agalactosylation	IgG4_Monogalactosylation	IgG4_Digalactosylation	IgG4_Sialylation	IgG4_Bisecting
IgG1_Agalactosylation	1,00	-0,64	-0,90	-0,62	0,26	0,81	-0,63	-0,76	-0,57	0,21	0,67	-0,39	-0,62	-0,47	0,22
IgG1_Monogalactosylation	-0,64	1,00	0,47	-0,09	0,00	-0,42	0,65	0,29	0,08	-0,02	-0,33	0,50	0,22	0,02	-0,06
IgG1_Digalactosylation	-0,90	0,47	1,00	0,46	-0,30	-0,81	0,56	0,87	0,53	-0,26	-0,62	0,31	0,67	0,40	-0,23
IgG1_Sialylation	-0,62	-0,09	0,46	1,00	-0,26	-0,51	0,15	0,47	0,62	-0,17	-0,50	0,04	0,44	0,60	-0,19
IgG1_Bisecting	0,26	0,00	-0,30	-0,26	1,00	0,28	-0,15	-0,31	-0,23	0,84	0,28	-0,12	-0,31	-0,19	0,60
IgG2_Agalactosylation	0,81	-0,42	-0,81	-0,51	0,28	1,00	-0,72	-0,93	-0,77	0,28	0,61	-0,30	-0,60	-0,46	0,24
IgG2_Monogalactosylation	-0,63	0,65	0,56	0,15	-0,15	-0,72	1,00	0,56	0,20	-0,17	-0,44	0,41	0,39	0,18	-0,12
IgG2_Digalactosylation	-0,76	0,29	0,87	0,47	-0,31	-0,93	0,56	1,00	0,68	-0,28	-0,58	0,23	0,64	0,43	-0,23
IgG2_Sialylation	-0,57	0,08	0,53	0,62	-0,23	-0,77	0,20	0,68	1,00	-0,22	-0,46	0,10	0,42	0,50	-0,23
IgG2_Bisecting	0,21	-0,02	-0,26	-0,17	0,84	0,28	-0,17	-0,28	-0,22	1,00	0,23	-0,11	-0,25	-0,15	0,58
IgG4_Agalactosylation	0,67	-0,33	-0,62	-0,50	0,28	0,61	-0,44	-0,58	-0,46	0,23	1,00	-0,56	-0,91	-0,75	0,25
IgG4_Monogalactosylation	-0,39	0,50	0,31	0,04	-0,12	-0,30	0,41	0,23	0,10	-0,11	-0,56	1,00	0,34	-0,06	-0,39
IgG4_Digalactosylation	-0,62	0,22	0,67	0,44	-0,31	-0,60	0,39	0,64	0,42	-0,25	-0,91	0,34	1,00	0,69	-0,19
IgG4_Sialylation	-0,47	0,02	0,40	0,60	-0,19	-0,46	0,18	0,43	0,50	-0,15	-0,75	-0,06	0,69	1,00	0,00
IgG4_Bisecting	0,22	-0,06	-0,23	-0,19	0,60	0,24	-0,12	-0,23	-0,23	0,58	0,25	-0,39	-0,19	0,00	1,00
